# Supplementary material for: Computational Study of Selected Amine and Lactam N-Oxides Including Comparisons of N-O Bond Dissociation Enthalpies with Those of Pyridine N-Oxides
Source: Molecules. 2020 Aug 14;25(16):3703. doi: 10.3390/molecules25163703 (PMC7463812; doi:10.3390/molecules25163703)
Supplement: Supplementary file 1 [file molecules-25-03703-s001.pdf]

Supporting Information

Computational Study of Selected Amine and Lactam N-Oxides Including Comparisons of N-O Bond Dissociation Enthalpies with Those of Pyridine N-Oxides

Arthur Greenberg[a], Alexa R. Green[a], Joel F. Liebman[b]**

a. Department of Chemistry, University of New Hampshire, Durham, NH 03824 USA
b. Department of Chemistry and Biochemistry, University of Maryland, Baltimore County, Baltimore, MD 21250 USA

*E-mail: art.greenberg@unh.edu, jliebman@umbc.edu

Cartesian Coordinates (Å) and energies (Hartrees) for all structures.

Quinuclidine
The number of imaginary frequencies: 0
Total Energy (B3LYP/6-31G*)= -329.31071302
ZPE= -329.114741
Enthalpy= -329.107259

N	0	0	1.29386
C	1.20023	0.69295	0.79983
H	1.19626	1.70683	1.2161
H	2.07629	0.18258	1.2161
C	1.25408	0.72404	-0.76174
H	1.27808	1.75683	-1.13422
H	2.1605	0.22844	-1.13422
C	0	0	-1.29494
H	0	0	-2.39115
C	0	-1.3859	0.79983
H	-0.88003	-1.88941	1.2161
H	0.88003	-1.88941	1.2161
C	0.	-1.44808	-0.76174
H	0.88241	-1.98527	-1.13422
H	-0.88241	-1.98527	-1.13422
C	-1.20023	0.69295	0.79983
C	-1.25408	0.72404	-0.76174
H	-1.27808	1.75683	-1.13422
H	-2.1605	0.22844	-1.13422
H	-1.19626	1.70683	1.2161
H	-2.07629	0.18258	1.2161

Quinuclidine
The number of imaginary frequencies: 1 (-100.16 cm^{-1})
Total Energy (M06/6-311G+(d,p))= -329.13823295
ZPE=-328.945118
Enthalpy=-328.938334

N	0.	0.	1.29386
C	1.20023	0.69295	0.79983
H	1.19626	1.70683	1.2161
H	2.07629	0.18258	1.2161
C	1.25408	0.72404	-0.76174
H	1.27808	1.75683	-1.13422
H	2.1605	0.22844	-1.13422
C	0.	0.	-1.29494
H	0.	0.	-2.39115
C	0.	-1.3859	0.79983
H	-0.88003	-1.88941	1.2161
H	0.88003	-1.88941	1.2161
C	0.	-1.44808	-0.76174
H	0.88241	-1.98527	-1.13422

H -0.88241 -1.98527 -
1.13422

C -1.20023 0.69295
0.79983

C -1.25408 0.72404 -
0.76174

H -1.27808 1.75683 -
1.13422

H -2.1605 0.22844 -
1.13422

H -1.19626 1.70683
1.2161

H -2.07629 0.18258
1.2161

Quinuclidinium
The number of imaginary frequencies: 0
Total Energy (B3LYP/6-31G*)= -
329.70138418
ZPE= -329.489673
Enthalpy= -329.481994

N 0. 0.
1.23295

C -0.68429 1.27048
0.75301

H -1.67443 1.28443
1.2142

H -0.10465 2.10415
1.15603

C -0.73219 1.25406 -
0.79426

H -1.77146 1.25419 -
1.13569

H -0.26305 2.16015 -
1.18807

C 0. 0. -
1.31307

H 0. 0. -
2.40598

C 1.44241 -0.04263
0.75301

H 1.87457 -0.96144
1.15603

H 1.94956 0.80788
1.2142

C 1.45214 0.00706 -
0.79426

H 1.97189 0.90703 -
1.13569

H 2.00227 -0.85227 -
1.18807

C -0.75812 -1.22785
0.75301

C -0.71995 -1.26112 -
0.79426

H -1.73922 -1.30788 -
1.18807

H -0.20043 -2.16122 -
1.13569

H -1.76992 -1.1427
1.15603

H -0.27514 -2.09231
1.2142

H 0. 0.
2.25686

Quinuclidinium

The number of imaginary frequencies: 0

Total Energy (M06/6-311G+(d,p))= -
329.52003854
ZPE= -329.31107
Enthalpy= -329.303622

N 0. 0.
1.25297

C -1.36295 -0.14344
0.72939

H -1.94822 -0.86846
1.25542

H -1.82127 0.81898
0.82214

C -1.26368 -0.61635 -
0.76063

H -1.1531 -1.67669 -
0.85204

H -2.15885 -0.31144 -
1.26123

C 0. 0. -
1.32633

H 0. 0. -
2.39633

C 0.55726 1.25207
0.72939

H 1.61989 1.16778
0.82214

H 0.222 2.12144
1.25542

C 0.09807 1.40255 -
0.76063

H -0.8755 1.83695 -
0.85204

H 0.80971 2.02534 -
1.26123

C 0.8057 -1.10863
0.72939

C 1.16561 -0.7862 -
0.76063

H 1.34914 -1.7139 -
1.26123

H 2.0286 -0.16027 -
0.85204

H 0.20138 -1.98675
0.82214

H 1.72622 -1.25298
1.25542

H 0. 0.
2.25297

2-Quinuclidone

The number of imaginary frequencies: 0
Total Energy (B3LYP/6-31G*)= -
403.32618144
ZPE= -403.150004
Enthalpy= -403.141759

N 0.48691 -1.06745 -
0.16205

C -0.47643 -0.92982 -
1.2825

H 0.10416 -0.82594 -
2.20483

H -1.01409 -1.88145 -
1.35346

C -1.45882 0.26756 -
1.09075

H -1.45729 0.91808 -
1.9738

H -2.4884 -0.08465 -
0.95483

C -1.00067 1.04991
0.15388

H -1.60802 1.95146
0.28997

C -0.26567 -1.15544
1.11669

H 0.4551 -1.33621
1.91978

H -0.89881 -2.04654
1.04599

C -1.11731 0.12815
1.38572

H -2.16771 -0.1301
1.56529

H -0.76027 0.65226
2.2809

C 1.30668 0.132 -
0.09005

C 0.48355 1.42132 -
0.03363

H 0.64315 1.99334 -
0.95814

H 0.86813 2.03991
0.78643

O 2.51097 0.09248 -
0.03314

2-Quinuclidone

The number of imaginary frequencies: 0
Total Energy (M06/6-311G+(d,p))= -403.15381144
ZPE= -402.97944
Enthalpy= -402.971321

N 0.48691 -1.06745 -0.16205

C -0.47643 -0.92982 -1.2825

H 0.10416 -0.82594 -2.20483

H -1.01409 -1.88145 -1.35346

C -1.45882 0.26756 -1.09075

H -1.45729 0.91808 -1.9738

H -2.4884 -0.08465 -0.95483

C -1.00067 1.04991 0.15388

H -1.60802 1.95146 0.28997

C -0.26567 -1.15544 1.11669

H 0.4551 -1.33621 1.91978

H -0.89881 -2.04654 1.04599

C -1.11731 0.12815 1.38572

H -2.16771 -0.1301 1.56529

H -0.76027 0.65226 2.2809

C 1.30668 0.132 -0.09005

C 0.48355 1.42132 -0.03363

H 0.64315 1.99334 -0.95814

H 0.86813 2.03991 0.78643

O 2.51097 0.09248 -0.03314

Quinuclidine N-oxide
The number of imaginary frequencies: 0
Total Energy (B3LYP/6-31G*)= -404.45851715
ZPE= -404.258001
Enthalpy= -404.249666

N 0. 0. -1.01494

C 0. 1.41894 -0.47914

H 0.88088 1.8794 -0.92876

H -0.88088 1.8794 -0.92876

C 0. 1.44852 1.06897

H 0.87717 1.98524 1.44451

H -0.87717 1.98524 1.44451

C 0. 0. 1.59707

H 0. 0. 2.69282

C -1.22884 -0.70947 -0.47914

H -1.18717 -1.70256 -0.92876

H -2.06805 -0.17684 -0.92876

C -1.25445 -0.72426 1.06897

H -2.15786 -0.23297 1.44451

H -1.28069 -1.75228 1.44451

C 1.22884 -0.70947 -0.47914

C 1.25445 -0.72426 1.06897

H 2.15786 -0.23297 1.44451

H 1.28069 -1.75228 1.44451

H 2.06805 -0.17684 -0.92876

H 1.18717 -1.70256 -0.92876

O 0. 0. -2.36102

Quinuclidine N-oxide
The number of imaginary frequencies: 1 (-111.32 cm^{-1})
Total Energy (M06/6-311G+(d,p))= -404.28384052
ZPE= -404.085782
Enthalpy= -404.078242

N 0. 0. 1.01487

C 0. 1.41873 0.47911

H -0.88115 1.87886 0.92935

H 0.88115 1.87886 0.92935

C 0. 1.44783 -1.06941

H -0.88029 1.98289 -1.44447

H 0.88029 1.98289 -1.44447

C 0. 0. -1.59726

H 0. 0. -2.693

C 1.22866 -0.70937 0.47911

H 1.18657 -1.70253 0.92935

H 2.06772 -0.17633 0.92935

C 1.25386 -0.72392 -1.06941

H 2.15737 -0.22909 -1.44447

H 1.27709 -1.7538 -1.44447

C -1.22866 -0.70937 0.47911

C -1.25386 -0.72392 -1.06941

H -2.15737 -0.22909 -1.44447

H -1.27709 -1.7538 -1.44447

H -2.06772 -0.17633 0.92935

H -1.18657 -1.70253 0.92935

O 0. 0. 2.36108

3-Quinuclidone
The number of imaginary frequencies: 0
Total Energy (B3LYP/6-31G*)= -403.32323303
ZPE=-403.146709
Enthalpy=-403.138549

N 1.30151 0.00268 0.07525

C 0.85968 -0.73053 -1.15515

H 1.13165 -0.15168 -2.01297

H 1.31582 -1.69604 -1.22326

C -0.65184 -0.89571 -1.06607

C -1.27006 -0.03722 0.00742

H -2.33603 -0.00599 -0.07998

C 0.77916 -0.64701 1.32271

H 1.11197 -0.11233 2.18773

H 1.14509 -1.64872 1.4097

C -0.788 -0.66465 1.31416

H -1.12774 -1.66884 1.45939

H -1.15377 -0.17529 2.19259

C 0.78319 1.34823 -0.19065

C -0.72455 1.3881 0.07461

H -1.15776 2.06744 -0.62947

H -0.9451 1.71401 1.06963

H 0.98445 1.50441 -1.22988

H 1.24379 2.12251 0.38662

O -1.3034 -1.71942 -1.75928

2-Quinuclidone N-oxide
The number of imaginary frequencies: 0
Total Energy (B3LYP/6-31G*)= -478.46497585
ZPE= -478.285174
Enthalpy= -478.275977

N 0.48691 -1.06745 -0.16205

C -0.47643 -0.92982 -1.2825

H 0.10416 -0.82594 -2.20483

H -1.01409 -1.88145 -1.35346

C -1.45882 0.26756 -1.09075

H -1.45729 0.91808 -1.9738

H -2.4884 -0.08465 -0.95483

C -1.00067 1.04991 0.15388

H -1.60802 1.95146 0.28997

C -0.26567 -1.15544 1.11669

H 0.4551 -1.33621 1.91978

H -0.89881 -2.04654 1.04599

C -1.11731 0.12815 1.38572

H -2.16771 -0.1301 1.56529

H -0.76027 0.65226 2.2809

C 1.30668 0.132 -0.09005

C 0.48355 1.42132 -0.03363

H 0.64315 1.99334 -0.95814

H 0.86813 2.03991 0.78643

O 2.51097 0.09248 -0.03314

O 1.25655 -2.1726 -0.35145

2-Quinuclidone N-oxide
The number of imaginary frequencies: 0
Total Energy (M06/6-311G+(d,p))= -478.28851616
ZPE= -478.110506
Enthalpy= -478.10135

N	-0.67054	-0.6227	-0.00782
C	-0.15289	-0.66018	1.36405
H	-0.82027	-0.01968	1.90189
H	-0.12816	-1.6228	1.83058
C	1.29808	-0.06695	1.34959
H	1.42597	0.53406	2.22556
H	2.02114	-0.85565	1.34356
C	1.55511	0.66218	-0.00696
H	2.49225	1.17852	0.00083
C	0.19732	-1.11255	-1.09119
H	-0.24096	-0.70818	-1.97961
H	0.25144	-2.1784	-1.16835
C	1.61915	-0.5732	-0.90293
H	2.22711	-1.26633	-0.35993
H	2.0442	-0.40605	-1.87055
C	-0.82698	0.83476	-0.16982
C	0.44957	1.62091	-0.43374
H	0.44611	2.51858	0.14856
H	0.56724	1.88245	-1.46459
O	-1.94144	1.40318	-0.03392
O	-1.86211	-1.26911	-0.11698

Manxine

The number of imaginary frequencies: 0
Total Energy (B3LYP/6-31G*)= -447.2362167
ZPE= -446.952794
Enthalpy= -446.942197

N	0.	0.	-1.50549
C	1.25569	-0.70402	-1.34554
H	1.93774	-0.43415	-2.17034
C	2.01641	-0.466	-0.02819
H	2.35145	0.5795	-0.00373
H	2.93968	-1.06269	-0.06589
C	1.29336	-0.78848	1.29027
H	2.02423	-0.60433	2.08954
H	1.0809	-1.86591	1.33775
C	0.	0.	1.66239
H	0.	0.	2.7612
C	0.03616	1.51433	1.29027
C	-0.01814	1.43947	-1.34554
H	-0.59289	1.89521	-2.17034
H	1.00902	1.80214	-1.46403
H	1.05619	-1.77491	-1.46403
H	-0.48875	2.0552	2.08954
H	1.07548	1.86904	1.33775
C	-1.23755	-0.73545	-1.34554
H	-1.34485	-1.46106	-2.17034
H	-2.06521	-0.02723	-1.46403
C	-1.41177	-1.51326	-0.02819
H	-2.39016	-2.01449	-0.06589
H	-0.67386	-2.32616	-0.00373
C	-1.32953	-0.72585	1.29027
H	-1.53548	-1.45087	2.08954
H	-2.15637	-0.00313	1.33775

C -0.60464 1.97926 -0.02819

H -0.54952 3.07719 -0.06589

H -1.67759 1.74666 -0.00373

Manxine
The number of imaginary frequencies: 0
Total Energy (M06/6-311G+(d,p))= -
447.00374344
ZPE= -446.724038
Enthalpy= -446.713442

N 0. 0. 1.50482

C -0.4002 1.3839
1.34423

H 0.02092 1.98758
2.16679

C 0. 2.06906
0.02447

H 1.09425 2.15665 -
0.00405

H -0.37103 3.10387
0.06145

C -0.48207 1.43455 -
1.29097

H -0.14496 2.10557 -
2.09283

H -1.58005 1.46555 -
1.33254

C 0. 0. -
1.66213

H 0. 0. -
2.76084

C 1.48339 -0.29979 -
1.29097

C 1.39859 -0.34537
1.34423

H 1.71084 -1.01191
2.16679

H 1.98365 0.57467
1.46811

H -1.4895 1.43056
1.46811

H 1.89596 -0.92725 -
2.09283

H 2.05923 0.63559 -
1.33254

C -0.99839 -1.03853
1.34423

H -1.73176 -0.97567
2.16679

H -0.49415 -2.00522
1.46811

C -1.79186 -1.03453
0.02447

H -2.50252 -1.87326
0.06145

H -2.41484 -0.13068 -
0.00405

C -1.00133 -1.13476 -
1.29097

H -1.751 -1.17832 -
2.09283

H -0.47917 -2.10114 -
1.33254

C 1.79186 -1.03453
0.02447

H 2.87355 -1.23061
0.06145

H 1.3206 -2.02597 -
0.00405

Manxinium
The number of imaginary frequencies: 0
Total Energy (B3LYP/6-31G*)= -
447.62559456
ZPE= -447.326046
Enthalpy= -447.315473

N 0. 0. -
1.65093

C -1.47473 -0.26177 -
1.30701

H -1.83856 -0.89169 -
2.12412

C -1.77459 -0.96248
0.01522

H -1.31743 -1.95812
0.00671

H -2.85495 -1.15186 -
0.02916

C -1.47847 -0.26269
1.3514

H -1.90479 -0.92023
2.11786

H -2.05466 0.66906
1.41891

C 0. 0.
1.73636

H 0. 0.
2.8323

C 0.96673 -1.14905
1.3514

C 0.96407 -1.14627 -
1.30701

H 1.69151 -1.14639 -
2.12412

H 0.37656 -2.0671 -
1.38564

H -1.97844 0.70744 -
1.38564

H 1.74934 -1.18948
2.11786

H 0.44791 -2.11392
1.41891

C 0.51066 1.40804 -
1.30701

H 0.14705 2.03808 -
2.12412

H 1.60188 1.35966 -
1.38564

C 0.05377 2.01808
0.01522

H 0.42993 3.04839 -
0.02916

H -1.03707 2.11998
0.00671

C 0.51174 1.41174 1.3514

H 0.15545 2.10971
2.11786

H 1.60675 1.44486
1.41891

C 1.72082 -1.0556
0.01522

H 2.42502 -1.89653 -
0.02916

H 2.35449 -0.16187
0.00671

H 0. 0. -
2.67293

Manxinium
The number of imaginary frequencies: 0
Total Energy (M06/6-311G+(d,p))= -
447.38206771
ZPE= -447.08768
Enthalpy= -447.076867

N 0. 0.
1.65086

C -1.29674 0.74867
1.3086

H -1.42222 1.46482
2.12604

C -1.34158 1.51034 -
0.01308

H -0.57486 2.29273 -
0.00362

H -2.29413 2.05408
0.03174

C -1.29978 0.75429 -
1.35076

H -1.47507 1.51965 -
2.11567

H -2.15928 0.0753 -
1.42147

C 0. 0. -
1.73623

H 0. 0. -
2.83218

C 1.30312 0.7485 -
1.35076

C 1.29674 0.74867 1.3086

H 1.97968 0.49926
2.12604

H 1.06111 1.81232
1.38802

H -2.10007 0.01279
1.38802

H 2.05359 0.51762 -
2.11567

H 1.14485 1.83234 -
1.42147

C 0. -1.49734
1.3086

H -0.55747 -1.96409
2.12604

H 1.03896 -1.82511
1.38802

C -0.6372 -1.91702 -
0.01308

H -0.63182 -3.01381
0.03174

H -1.69813 -1.64421 -
0.00362

C -0.00334 -1.50278 -
1.35076

H -0.57852 -2.03727 -
2.11567

H 1.01443 -1.90764 -
1.42147

C 1.97878 0.40668 -
0.01308

H 2.92595 0.95973
0.03174

H 2.27299 -0.64852 -
0.00362

H 0. 0.
2.67284

2-Manxinone

The number of imaginary frequencies: 0
Total Energy (B3LYP/6-31G*)= -
521.27190837
ZPE= -521.006569
Enthalpy= -520.995563

N 0.80394 -1.29291
0.76415

C 0.89996 -0.16991
1.69203

H 1.8352 -0.27835
2.20042

C 1.00103 1.21541
0.98796

H 1.79145 1.11764
0.27342

H 1.25941 1.9053
1.76397

C -0.18804 1.90255
0.24047

H 0.23473 2.82474 -
0.09973

H -0.98355 2.07734
0.93438

C -0.82696 1.24119 -
1.02075

H -1.31757 2.02932 -
1.55278

C 0.18879 0.63804 -
2.03208

C 1.47923 -0.89513 -
0.44837

H 0.09331 -0.2046
2.39418

H -0.32274 0.54399 -
2.96716

H 1.01237 1.31503 -
2.12321

C -0.57773 -1.77244
0.56656

H -0.78678 -2.38318
1.41991

H -0.62918 -2.35256 -
0.33106

C -1.68719 -0.70787
0.5735

H -2.60424 -1.25118
0.66695

H -1.51059 -0.09666
1.43381

C -1.92502 0.23826 -
0.62781

H -2.76453 0.82579 -
0.31972

H -2.16073 -0.33476 -
1.50015

C 0.69288 -0.76784 -
1.72002

H 1.32991 -1.0675 -
2.52582

H -0.15299 -1.41986 -
1.65453

O 2.7229 -0.70318 -
0.44991

2-Manxinone
The number of imaginary frequencies: 0
Total Energy (M06/6-311G+(d,p))= -
521.03877972
ZPE= -520.776381
Enthalpy= -520.765493

N 0.74085 -0.74859 -
0.81454

C 0.89791 -1.73576
0.25992

H 1.91683 -2.1362
0.25716

C 0.60456 -1.09685
1.62999

H 1.42802 -0.41175
1.85282

H 0.65544 -1.88546
2.39353

C -0.73939 -0.35062
1.82445

H -0.69949 0.03538
2.85163

H -1.56002 -1.08259
1.82982

C -1.19989 0.84575
0.91861

H -1.86629 1.40855
1.5871

C -0.13642 1.91422
0.51782

C 1.44716 0.42717 -
0.63374

H 0.2164 -2.56662
0.05129

H -0.65989 2.87473
0.4237

H 0.58075 2.0371
1.33898

C -0.40468 -0.88734 -
1.70976

H -0.29501 -1.84493 -
2.23844

H -0.33479 -0.11343 -
2.4787

C -1.78115 -0.85433 -
1.03562

H -2.53913 -1.00911 -
1.81615

H -1.86929 -1.71978 -
0.36664

C -2.12954 0.42297 -
0.2565

H -3.13662 0.27149
0.15405

H -2.22359 1.26574 -
0.95605

C 0.6687 1.71579 -
0.79817

H 1.37723 2.54008 -
0.91446

H -0.00837 1.72584 -
1.65602

O 2.55853 0.43064 -
0.11424

Manxine N-oxide
The number of imaginary frequencies: 0
Total Energy (B3LYP/6-31G*)= -
522.37309036
ZPE= -522.084864
Enthalpy= -522.073635

N 0. 0.
1.49873

C 1.20619 -0.83645
1.07701

H 1.23698 -1.56295
1.88963

C 1.21855 -1.58537 -
0.25855

H 0.39192 -2.30539 -
0.26814

H 2.12555 -2.20497 -
0.21502

C 1.24463 -0.83686 -
1.60064

H 1.37697 -1.6098 -
2.36945

H 2.14476 -0.21025 -1.662

C 0. 0. -
1.99638

H 0. 0. -
3.09454

C -1.34706 -0.65945 -
1.60064

C -1.32748 -0.62636
1.07701

H -1.97204 -0.28978
1.88963

H -1.19287 -1.70548
1.19502

H 2.07343 -0.18031
1.19502

H -2.08261 -0.38759 -
2.36945

H -1.25446 -1.75229 -1.662

C 0.1213 1.46281
1.07701

H 0.73506 1.85273
1.88963

H -0.88056 1.8858
1.19502

C 0.7637 1.84798 -
0.25855

H 0.84679 2.94326 -
0.21502

H 1.80057 1.4921 -
0.26814

C 0.10243 1.49631 -
1.60064

H 0.70565 1.99739 -
2.36945

H -0.8903 1.96254 -1.662

C -1.98224 -0.26261 -
0.25855

H -2.97234 -0.73829 -
0.21502

H -2.19248 0.81328 -
0.26814

O 0. 0.
2.87272

Manxine N-oxide
The number of imaginary frequencies: 0
Total Energy (M06/6-311G+(d,p))= -
522.13655167
ZPE= -521.850767

Enthalpy= -521.839841

N 0. 0.
1.49875

C 0.9879 1.08547 1.0765

H 1.7121 1.02149
1.88921

C 1.73182 0.99987 -
0.25914

H 2.33933 0.08721 -0.269

H 2.46379 1.81892 -
0.21541

C 0.99256 1.12389 -
1.6009

H 1.77581 1.15458 -
2.37006

H 0.48846 2.09794 -
1.66155

C 0. 0. -
1.99633

H 0. 0. -
3.09449

C 0.47703 -1.42152 -
1.6009

C 0.44609 -1.39828
1.0765

H 0.02859 -1.99347
1.88921

H 1.53255 -1.40871
1.19477

H 0.4537 2.03158
1.19477

H 0.11199 -2.11518 -
2.37006

H 1.57264 -1.47199 -
1.66155

C -1.43399 0.31281
1.0765

H -1.74069 0.97198
1.88921

H -1.98625 -0.62287
1.19477

C -1.73182 0.99987 -
0.25914

H -2.80713 1.22424 -
0.21541

H -1.24519 1.98232 -0.269

C -1.46959 0.29764 -
1.6009

H -1.8878 0.96061 -
2.37006

H -2.0611 -0.62595 -
1.66155

C 0. -1.99974 -
0.25914

H 0.34334 -3.04316 -
0.21541

H -1.09414 -2.06953 -0.269

O 0. 0.
2.87264

2-Manxinone N-oxide
The number of imaginary frequencies: 0
Total Energy (B3LYP/6-31G*)= -
596.38238012
ZPE= -596.113899
Enthalpy= -596.101871

N 0.74085 -0.74859 -
0.81454

C 0.89791 -1.73576
0.25992

H 1.91683 -2.1362
0.25716

C 0.60456 -1.09685
1.62999

H 1.42802 -0.41175
1.85282

H 0.65544 -1.88546
2.39353

C -0.73939 -0.35062
1.82445

H -0.69949 0.03538
2.85163

H -1.56002 -1.08259
1.82982

C -1.19989 0.84575
0.91861

H -1.86629 1.40855
1.5871

C -0.13642 1.91422
0.51782

C 1.44716 0.42717 -
0.63374

H 0.2164 -2.56662
0.05129

H -0.65989 2.87473
0.4237

H 0.58075 2.0371
1.33898

C -0.40468 -0.88734 -
1.70976

H -0.29501 -1.84493 -
2.23844

H -0.33479 -0.11343 -
2.4787

C -1.78115 -0.85433 -
1.03562

H -2.53913 -1.00911 -
1.81615

H -1.86929 -1.71978 -
0.36664

C -2.12954 0.42297 -
0.2565

H -3.13662 0.27149
0.15405

H -2.22359 1.26574 -
0.95605

C 0.6687 1.71579 -
0.79817

H 1.37723 2.54008 -
0.91446

H -0.00837 1.72584 -
1.65602

O 2.55853 0.43064 -
0.11424

O 1.60399 -1.26199 -
1.73161

2-Manxinone N-oxide
The number of imaginary frequencies: 0
Total Energy (M06/6-311G+(d,p))= -
596.1437104
ZPE= -595.878736
Enthalpy= -595.866701

N -1.24978 0.5571 -
0.06119

C -1.0225 0.60808
1.44275

H -1.99614 0.32254
1.84006

C 0.05112 -0.3461
1.99545

H -0.32017 -1.3668
1.87142

H 0.05636 -0.17325
3.08038

C 1.53125 -0.29909
1.54263

H 2.01883 -1.0936
2.12246

H 1.99359 0.63328
1.89433

C 1.95887 -0.50255
0.05467

H 2.98751 -0.87854 0.134

C 1.22857 -1.61459 -
0.74767

C -1.13324 -0.89156 -
0.51665

H -0.84036 1.65658
1.69348

H 1.93006 -1.98733 -
1.50431

H 1.00543 -2.46359 -
0.0901

C -0.44238 1.62103 -
0.79406

H -1.04081 2.50436 -
0.56459

H -0.59598 1.41599 -
1.85404

C 1.01861 1.89669 -
0.44508

H 1.27842 2.79434 -
1.02325

H 1.08852 2.20756
0.60385

C 2.0816 0.82927 -
0.72831

H 3.04964 1.28093 -
0.47586

H 2.12683 0.62149 -
1.80638

C -0.07032 -1.24365 -
1.51772

H -0.41742 -2.12841 -
2.05893

H 0.12513 -0.45563 -
2.24556

O -1.80659 -1.71618
0.04237

O -2.55721 0.82433 -
0.40545

3-Manxinone
The number of imaginary frequencies: 0
Total Energy (B3LYP/6-31G*)= -
521.24887005
ZPE=-520.985183
Enthalpy=-520.973649

N 0.34309 -0.8798 -0.4157

C 1.73533 -1.10397 -
0.24132

H 1.89773 -1.90784
0.44594

H 2.24126 -1.33272 -
1.15599

C 2.19856 0.22534
0.21856

C 1.60255 1.27663 -
0.73356

H 2.3743 1.99549 -
0.91392

H 1.34593 0.75851 -
1.63389

C 0.3535 2.10284 -
0.29984

H 0.16683 2.73441 -
1.14315

H 0.65213 2.66865
0.55782

C -1.08113 1.49678
0.08272

H -1.70771 2.36316 0.124

C -0.25066 -1.30017
0.83751

H 0.21688 -2.2176
1.12842

H -1.30631 -1.45255
0.75212

C -0.02431 -0.2272
1.82819

H -0.21424 -0.58294
2.81929

H 0.99038 0.10556
1.76054

C -1.03224 0.90507
1.54016

H -0.77882 1.7011
2.20875

H -2.01535 0.53682
1.74701

C -1.8688 0.51282 -1.0041

H -2.90544 0.61422 -
0.75916

H -1.65387 0.9151 -
1.97202

C -1.68767 -1.0849 -
1.07333

H -1.94986 -1.47322 -
0.11137

H -2.34727 -1.47227 -
1.82151

C -0.3001 -1.55212 -
1.39156

H -0.19954 -2.61501 -
1.32036

H 0.03026 -1.25867 -
2.36606

O 2.99053 0.44946
1.17046

4-Manxinone
The number of imaginary frequencies: 0
Total Energy (B3LYP/6-31G*)= -
521.25239348
ZPE=-520.988551
Enthalpy=-520.977154

N 0.13307 0.84165
0.50607

C 1.36254 1.48787
0.15671

H 1.25326 2.5441
0.02506

H 2.0895 1.31269
0.92205

C 1.79716 0.75911 -
1.05743

H 1.17199 0.90246 -
1.91388

H 2.7932 1.04175 -
1.32746

C 1.69686 -0.65932 -
0.51302

C 0.63667 -1.70843 -
0.91479

H 1.01328 -2.62704 -
0.51578

H 0.60213 -1.73605 -
1.98388

C -0.89826 -1.59071 -
0.47035

H -1.30093 -2.5581 -
0.68694

C -0.95111 1.47758 -
0.19979

H -0.7682 2.53162 -
0.22076

H -1.89441 1.29298
0.27033

C -1.00267 0.87996 -
1.55531

H -1.64869 1.45303 -
2.1871

H -0.01717 0.86828 -
1.97191

C -1.60079 -0.55925 -
1.44664

H -1.54726 -0.96554 -
2.43505

H -2.61817 -0.48052 -
1.12472

C -1.30617 -1.36232
1.11913

H -2.30158 -1.7457
1.20327

H -0.6146 -1.9441
1.69199

C -1.43609 0.08983
1.79697

H -2.15519 0.64393
1.23061

H -1.77434 -0.03232
2.80473

C -0.16503 0.87779
1.82821

H -0.30211 1.87002 2.20449

H 0.59122 0.40416 2.41868

O 2.55263 -1.00715 0.34152

1-azabicyclo[3.3.2]decane
The number of imaginary frequencies: 0
Total Energy (B3LYP/6-31G*)= -407.93010375
ZPE= -407.675878
Enthalpy= -407.666305

N -0.05842 -1.37398 0.34609

C -0.3559 -0.68727 1.60074

H -1.3261 -1.05222 1.96471

H 0.38935 -1.01717 2.33487

C -0.37665 0.88213 1.55362

H -1.38404 1.24663 1.80098

H 0.28604 1.27426 2.33829

C -1.10299 -1.34353 -0.68607

H -1.85267 -2.12557 -0.48037

H -0.63588 -1.60455 -1.64365

C -1.85207 0.00369 -0.81651

H -2.52528 -0.05781 -1.68409

H -2.50623 0.1239 0.05808

C -0.96944 1.26364 -0.94962

H -1.64785 2.12633 -1.00527

H -0.43759 1.24219 -1.91101

C 0.05511 1.51573 0.20652

H 0.04755 2.60142 0.39143

C 1.52958 1.19307 -0.18258

H 1.88833 1.99868 -0.84006

H 2.14566 1.24727 0.7286

C 1.7907 -0.14637 -0.89417

H 2.86552 -0.23568 -1.113

H 1.28597 -0.14617 -1.87016

C 1.3388 -1.38555 -0.0966

H 1.96294 -1.49142 0.80121

H 1.51049 -2.28541 -0.70523

1-azabicyclo[3.3.2]decane
The number of imaginary frequencies: 0
Total Energy (M06/6-311G+(d,p))= -407.7174344
ZPE= -407.466218
Enthalpy= -407.456724

N -0.52936 -1.46194 0.32938

C -0.4999 -0.61958 1.55951

H -1.39445 -0.78368 2.12321

H 0.35748 -0.97772 2.09012

C -0.37276 0.94423 1.43441

H -1.32872 1.38712
1.24763

H 0.00706 1.25658
2.38471

C -1.39231 -0.97358 -
0.75987

H -2.38026 -1.34752 -
0.58949

H -0.99921 -1.36158 -
1.67629

C -1.57078 0.55579 -
0.84377

H -2.17852 0.7321 -
1.70659

H -2.08256 0.83049
0.05486

C -0.37379 1.54878 -
0.93761

H -0.85206 2.50592 -
0.93125

H 0.21792 1.44221 -
1.82272

C 0.53225 1.46668
0.30102

H 0.87689 2.45012
0.54382

C 1.80044 0.67089
0.00631

H 2.42413 1.30108 -
0.59266

H 2.30039 0.42148
0.91886

C 1.54487 -0.56127 -
0.84054

H 2.49826 -0.94095 -
1.14351

H 0.9731 -0.26693 -
1.69573

C 0.8403 -1.69016 -
0.14503

H 1.42899 -1.97921
0.70043

H 0.78724 -2.48654 -
0.85767

1-azabicyclo[3.3.2]decanium
The number of imaginary frequencies: 0
Total Energy (B3LYP/6-31G*)= -
408.32021719
ZPE= -408.049901
Enthalpy= -408.040259

N -0.13461 -1.46116
0.25103

C -0.45336 -0.75998
1.5659

H -1.48185 -1.04842
1.79589

H 0.19091 -1.24937
2.30072

C -0.27534 0.77838
1.60733

H -1.21734 1.20183
1.97221

H 0.47155 1.00497
2.3749

C -1.13751 -1.17567 -
0.88011

H -1.8914 -1.96441 -
0.81055

H -0.59359 -1.31636 -
1.81601

C -1.81961 0.18839 -
0.80598

H -2.49203 0.22285 -
1.67202

H -2.47889 0.20793
0.06955

C -0.92954 1.44154 -
0.81253

H -1.60744 2.29785 -
0.72944

H -0.4409 1.54941 -
1.78855

C 0.13278 1.52877
0.31927

H 0.17684 2.57926
0.62952

C 1.56726 1.19241 -
0.14847

H 1.91299 1.99894 -
0.80538

H 2.23716 1.20336
0.72201

C 1.74233 -0.1234 -
0.91218

H 2.80232 -0.25826 -
1.1601

H 1.22535 -0.07998 -
1.87719

C 1.33692 -1.38574 -
0.16662

H 1.90929 -1.51004
0.7576

H 1.50335 -2.26801 -
0.79192

H -0.2677 -2.45168
0.47184

1-azabicyclo[3.3.2]decanium
The number of imaginary frequencies: 0
Total Energy (M06/6-311G+(d,p))= -
408.09697078
ZPE= -407.830573
Enthalpy= -407.820844

N -0.07348 -1.5252 0.2807

C -0.72639 -0.75417
1.37326

H -1.76988 -0.79575
1.14025

H -0.52932 -1.22082
2.31576

C -0.27496 0.74301
1.58603

H -1.07641 1.20627
2.12266

H 0.62385 0.74792
2.16656

C -0.86461 -1.25443 -
0.94671

H -1.65843 -1.97118 -
0.9787

H -0.23727 -1.36177 -
1.80684

C -1.6354 0.10511 -
0.88284

H -2.24253 0.15385 -
1.76256

H -2.2332 0.02185
0.00068

C -0.95169 1.48489 -
0.77639

H -1.75491 2.16005 -
0.56684

H -0.46429 1.76065 -
1.68815

C 0.08232 1.58778
0.34036

H 0.16837 2.61287
0.6348

C 1.47794 1.22474 -
0.1597

H 1.74693 1.95704 -
0.89202

H 2.1402 1.27065
0.67946

C 1.6508 -0.10168 -0.8213

H 2.67967 -0.15937 -
1.10942

H 1.01956 -0.14949 -
1.68394

C 1.35389 -1.30913
0.04248

H 1.88252 -1.26662 0.9718

H 1.68862 -2.1465 -0.53342

H -0.14051 -2.4999 0.49394

1-azabicyclo[3.3.2]decan-9-one
The number of imaginary frequencies: 0
Total Energy (B3LYP/6-31G*)= -481.95525515
ZPE= -481.719565
Enthalpy= -481.709548

N -0.64665 -0.90237 -0.70347

C -1.25312 0.36241 -0.78479

C -0.29365 1.53804 -0.82058

H 0.28174 1.60644 -1.75096

H -0.89531 2.44653 -0.73537

C 0.66184 -1.1245 -1.32772

C 1.91883 -0.76492 -0.51327

H 2.00177 -1.46061 0.32708

H 2.79206 -0.97122 -1.14713

C 1.99631 0.69356 -0.01215

H 2.43733 1.31653 -0.80264

H 2.69952 0.73973 0.83051

C 0.66862 1.36927 0.40102

H 0.94065 2.38957 0.70331

C -0.03103 0.74623 1.65421

H 0.54075 1.06507 2.5354

H -1.01933 1.21329 1.75497

C -0.18143 -0.7973 1.77078

H -0.7356 -1.00458 2.69614

H 0.81079 -1.23395 1.92366

C -0.90707 -1.52493 0.6034

H -1.98829 -1.49568 0.75107

H -0.60778 -2.57875 0.56292

H 0.71204 -2.18982 -1.58887

H 0.66025 -0.58328 -2.28011

O -2.44749 0.50876 -0.58513

1-azabicyclo[3.3.2]decan-2-one
The number of imaginary frequencies: 0
Total Energy (B3LYP/6-31G*)= -481.96668910
ZPE= -481.730531
Enthalpy= -481.720529

N 0.75111 0.30232 0.97785

C -0.37335 -0.1788 1.78213

H -0.03898 -1.06612 2.32769

H -0.55822 0.58449 2.54416

C -1.68016 -0.48331 0.99357

H -2.02513 -1.49614 1.2406

H -2.47706 0.19596
1.32084

C 1.40207 -0.55281
0.09792

C 0.6543 -1.82965 -
0.24261

H 1.3266 -2.48366 -
0.80306

H 0.30967 -2.37534
0.64158

C -0.56104 -1.42747 -
1.12743

H -1.13091 -2.34228 -
1.33273

H -0.18075 -1.07842 -
2.09565

C -1.5359 -0.3608 -0.5367

H -2.52639 -0.61541 -
0.93877

C -1.3267 1.11496 -
0.99936

H -1.60046 1.16847 -
2.0619

H -2.08156 1.71604 -
0.47013

C 0.04918 1.79951 -
0.85661

H -0.05811 2.85729 -
1.13317

H 0.75069 1.36739 -
1.57807

C 0.7002 1.70442
0.53332

H 1.71484 2.10969
0.49977

H 0.13635 2.26997
1.28309

O 2.40126 -0.20557 -
0.51931

1-azabicyclo[3.3.2]decane N-oxide

The number of imaginary frequencies: 0
Total Energy (B3LYP/6-31G*)= -
483.07072469
ZPE= -482.811552
Enthalpy= -482.801294

N 1.31918 0.03295
0.05803

C 0.74654 -0.33449
1.41918

H 1.21536 -1.29829
1.62283

H 1.23556 0.38861
2.07258

C -0.78765 -0.37766
1.65538

H -1.04452 -1.37771
2.02746

H -1.02292 0.3117
2.47575

C 0.94869 -0.99324 -
1.01594

H 1.81735 -1.65073 -
0.99387

H 0.95832 -0.46099 -
1.97097

C -0.32944 -1.81242 -
0.81966

H -0.39466 -2.48664 -
1.68466

H -0.18921 -2.47136
0.04626

C -1.67247 -1.07911 -
0.66782

H -2.4342 -1.85084 -
0.49923

H -1.94853 -0.59577 -
1.61411

C -1.74556 -0.04528
0.49069

H -2.75123 -0.12558
0.92491

C -1.62585 1.41793
0.01189

H -2.54997 1.68447 -
0.51872

H -1.57955 2.07481
0.89244

C -0.44675 1.72029 -
0.91491

H -0.46769 2.78648 -
1.17885

H -0.57478 1.18676 -
1.86479

C 0.94565 1.4495 -
0.35461

H 1.15461 2.05543
0.53114

H 1.71384 1.67808 -
1.09519

O 2.67861 0.03098
0.19946

1-azabicyclo[3.3.2]decane N-oxide
The number of imaginary frequencies: 0
Total Energy (M06/6-311G+(d,p))= -
482.85412341
ZPE= -482.598276
Enthalpy= -482.587988

N 1.30842 0.004
0.08824

C 0.69696 -0.68948
1.25429

H 0.76374 -1.72897
1.00956

H 1.25534 -0.47187
2.14071

C -0.78766 -0.31392
1.63571

H -1.1482 -1.13891
2.21389

H -0.77222 0.58198
2.22055

C 0.94203 -0.79639 -
1.10794

H 1.68902 -1.55336 -
1.22584

H 0.92187 -0.16219 -
1.9695

C -0.36228 -1.63425 -
0.89979

H -0.47866 -2.24072 -
1.7736

H -0.1518 -2.2295 -
0.03592

C -1.75366 -1.02055 -
0.63516

H -2.36086 -1.85702 -
0.35851

H -2.1529 -0.54511 -
1.50663

C -1.78244 0.00406
0.49454

H -2.77136 0.03805
0.9017

C -1.5466 1.41738 -
0.03129

H -2.36824 1.65144 -
0.67552

H -1.53139 2.07429
0.81318

C -0.31223 1.658 -
0.83467

H -0.33778 2.68922 -
1.11897

H -0.32968 1.03222 -
1.70243

C 0.99705 1.41943 -
0.11279

H 1.03225 1.94282
0.8198

H 1.74757 1.79707 -
0.77537

O 2.66092 -0.02165
0.22849

1-azabicyclo[3.3.2]decan-3-one

The number of imaginary frequencies: 0
Total Energy (B3LYP/6-31G*)= -481.94228995
ZPE=-481.707444
Enthalpy=-481.697043

N 0.24784 -1.47809 -0.31408

C 1.17167 -0.86629 -1.26571

H 2.11432 -1.42711 -1.23859

H 0.73918 -1.06019 -2.25151

C 1.48243 0.65839 -1.14241

H 2.45905 0.79972 -0.66385

H 1.59402 1.05998 -2.15674

C 0.56511 -1.35699 1.11992

H 1.22911 -2.18769 1.39502

H -0.36706 -1.5106 1.66795

C 1.23323 -0.05509 1.59846

H 1.25111 -0.09693 2.69428

H 2.28811 -0.07247 1.29337

C 0.62108 1.29167 1.17111

H 1.29209 2.07446 1.54082

H -0.33953 1.44781 1.67114

C 0.45468 1.49011 -0.36009

H 0.66203 2.54228 -0.58139

C -1.01375 1.27641 -0.84073

H -1.60925 2.15207 -0.57353

H -1.01842 1.17355 -1.93246

C -1.64919 0.07406 -0.17194

C -1.1787 -1.29218 -0.63867

H -1.29072 -1.40978 -1.72078

H -1.76825 -2.06403 -0.13861

O -2.38626 0.20063 0.79672

1-azabicyclo[3.3.2]decan-4-one
The number of imaginary frequencies: 0
Total Energy (B3LYP/6-31G*)= -481.94313071
ZPE=-481.708072
Enthalpy=-481.697750

N -0.0826 -1.58292 0.37087

C -0.29758 -0.71919 1.60982

H -1.10194 -1.13067 2.18305

H 0.63033 -0.80009 2.13646

C -0.64134 0.84077 1.47387

H -1.67873 1.0048 1.26937

H -0.38122 1.26562 2.42084

C -1.08122 -1.3715 -0.68321

H -1.9297 -1.98672 -0.46761

H -0.63077 -1.66776 -
1.60745

C -1.6537 0.05566 -
0.78895

H -2.29464 0.04806 -
1.64572

H -2.2113 0.21472
0.11031

C -0.74324 1.31174 -
0.91985

H -1.43988 2.12226 -
0.97144

H -0.12279 1.30884 -
1.7916

C 0.11968 1.51996
0.34057

H 0.23456 2.57344
0.48848

C 1.4771 0.93282
0.21238

C 1.5912 -0.11669 -
0.83308

H 2.58411 -0.12069 -
1.23183

H 0.89392 0.07704 -
1.62122

C 1.29119 -1.44075 -
0.20806

H 1.99911 -1.60948
0.57634

H 1.39826 -2.18361 -
0.97069

O 2.45266 1.30699
0.9137

1-azabicyclo[3.3.2]decan-10-one
The number of imaginary frequencies: 0
Total Energy (B3LYP/6-31G*)= -
481.94671758
ZPE=-481.711904
Enthalpy=-481.701618

N 0.91829 0.62851 -
1.05199

C -0.43599 0.42223 -
1.51024

H -0.87374 1.37496 -
1.83875

H -0.42279 -0.2177 -
2.40303

C -1.43948 -0.21465 -
0.52415

C 1.08582 1.66587 -
0.03367

H 1.17969 2.64655 -
0.52689

H 2.04107 1.48595
0.47312

C -0.04653 1.78292
1.0095

H 0.25644 2.53191
1.75346

H -0.93502 2.20465
0.51906

C -0.45921 0.49092
1.74019

H -1.28743 0.75039
2.40968

H 0.35756 0.15303
2.39076

C -0.92299 -0.69416
0.83545

H -1.79953 -1.15637
1.30094

C 0.13885 -1.82149
0.67977

H 0.14882 -2.38479
1.62213

H -0.2094 -2.52876 -
0.08682

C 1.57648 -1.38693
0.36854

H 2.21065 -2.28182
0.29874

H 1.9744 -0.80884
1.21283

C 1.74347 -0.56698 -
0.92313

H 1.5258 -1.20351 -
1.79129

H 2.79798 -0.27118 -
1.01586

O -2.60439 -0.34506 -
0.85096

1-azabicyclo[4.3.3]dodecane
The number of imaginary frequencies: 0
Total Energy (B3LYP/6-31G*)= -
486.54115606
ZPE= -486.229503
Enthalpy= -486.217542

N 0.40679 -1.27086 -
0.05346

C 1.83246 -1.13866 -
0.25423

H 2.39188 -1.84521
0.38658

H 2.06027 -1.4182 -
1.29071

C 2.35452 0.29 -0.01702

H 2.35545 0.52346
1.05516

H 3.40984 0.31516 -
0.32642

C 1.57118 1.36919 -
0.79145

H 2.27703 2.17634 -
1.03055

H 1.26307 0.96079 -
1.76375

C 0.36878 2.07362 -0.116

H 0.21612 2.99005 -
0.70364

H 0.68691 2.42642
0.8765

C -1.05047 1.43197
0.02848

H -1.69801 2.32043
0.05674

C -0.06841 -1.48695
1.29817

H 0.66282 -2.0887
1.86652

H -0.98842 -2.08664
1.25747

C -0.34699 -0.18671
2.06502

H -0.72789 -0.44805
3.06388

H 0.59997 0.33816
2.23842

C -1.34869 0.78041
1.41252

H -1.47394 1.61011
2.12229

H -2.33552 0.29767
1.36407

C -1.56258 0.66008 -
1.23628

H -2.54507 1.07838 -
1.49246

H -0.91708 0.90467 -
2.09112

C -1.75255 -0.86685 -
1.18859

H -2.38858 -1.13411 -
0.33472

H -2.31736 -1.17643 -
2.07995

C -0.45636 -1.68445 -
1.13898

H -0.71388 -2.76253 -
1.09212

H 0.08954 -1.53569 -
2.07775

1-azabicyclo[4.3.3]dodecane
The number of imaginary frequencies: 0
Total Energy (M06/6-311G+(d,p))= -
486.28835181
ZPE= -485.980448
Enthalpy= -485.96858

N 0.40679 -1.27086 -
0.05346

C 1.83246 -1.13866 -
0.25423

H 2.39188 -1.84521
0.38658

H 2.06027 -1.4182 -
1.29071

C 2.35452 0.29 -0.01702

H 2.35545 0.52346
1.05516

H 3.40984 0.31516 -
0.32642

C 1.57118 1.36919 -
0.79145

H 2.27703 2.17634 -
1.03055

H 1.26307 0.96079 -
1.76375

C 0.36878 2.07362 -0.116

H 0.21612 2.99005 -
0.70364

H 0.68691 2.42642
0.8765

C -1.05047 1.43197
0.02848

H -1.69801 2.32043
0.05674

C -0.06841 -1.48695
1.29817

H 0.66282 -2.0887
1.86652

H -0.98842 -2.08664
1.25747

C -0.34699 -0.18671
2.06502

H -0.72789 -0.44805
3.06388

H 0.59997 0.33816
2.23842

C -1.34869 0.78041
1.41252

H -1.47394 1.61011
2.12229

H -2.33552 0.29767
1.36407

C -1.56258 0.66008 -
1.23628

H -2.54507 1.07838 -
1.49246

H -0.91708 0.90467 -
2.09112

C -1.75255 -0.86685 -
1.18859

H -2.38858 -1.13411 -
0.33472

H -2.31736 -1.17643 -
2.07995

C -0.45636 -1.68445 -
1.13898

H -0.71388 -2.76253 -
1.09212

H 0.08954 -1.53569 -
2.07775

1-azabicyclo[4.3.3]dodecanium
The number of imaginary frequencies: 0
Total Energy (B3LYP/6-31G*)= -
486.92192592
ZPE= -486.593438
Enthalpy= -486.581482

C 1.84633 -1.23573 -
0.18455

H 2.23727 -1.97713
0.51481

H 2.03179 -1.59057 -
1.20095

C 2.43169 0.16783
0.01312

H 2.44927 0.4341
1.07658

H 3.47914 0.12471 -
0.30393

C 1.69783 1.26324 -
0.81073

H 2.43494 2.05025 -
0.99869

H 1.45599 0.86859 -
1.80932

C 0.46654 2.03096 -
0.22076

H 0.36053 2.89184 -
0.88939

H 0.76181 2.45263
0.74898

C -1.00405 1.46474 -
0.05734

H -1.59013 2.38751 -
0.09879

C -0.18017 -1.40018
1.39387

H 0.55001 -2.02474
1.91228

H -1.10598 -1.97059
1.29648

C -0.41634 -0.06605
2.09908

H -0.84458 -0.2879
3.08311

H 0.54807 0.41665
2.29747

C -1.35834 0.91937
1.36972

H -1.43125 1.79324
2.02619

H -2.37004 0.49504
1.33759

C -1.57703 0.63827 -
1.28187

H -2.5632 1.05683 -
1.50446

H -0.96831 0.85005 -
2.17136

C -1.80441 -0.89396 -
1.17825

H -2.45852 -1.12107 -
0.32882

H -2.35307 -1.22034 -
2.06849

C -0.52499 -1.73123 -
1.10231

H -0.70022 -2.78709 -0.881

H 0.04258 -1.65634 -
2.03326

N 0.34179 -1.14063 -
0.01385

H 0.48543 -2.12234
0.11112

1-azabicyclo[4.3.3]dodecanium
The number of imaginary frequencies: 0
Total Energy (M06/6-311G+(d,p))= -
486.65734236
ZPE= -486.333333
Enthalpy= -486.321383

C 1.78464 1.05327 -
0.42185

H 2.58849 1.37237
0.20814

H 1.99279 1.23089 -
1.45627

C 0.64772 2.03817 -
0.07261

H 0.37255 2.00791
0.96096

H 1.09012 2.98158 -
0.31585

C -0.60595 1.92852 -
0.9109

H -0.96681 2.92533 -
1.05601

H -0.3363 1.48455 -
1.84635

C -1.82119 1.24406 -
0.26227

H -2.57472 1.3084 -
1.01921

H -2.06182 1.84388
0.59049

C -1.9164 -0.2029
0.25507

H -2.95983 -0.31354
0.46461

C 1.56765 -0.73906
1.12268

H 2.42599 -0.36278
1.63899

H 1.49359 -1.79984
1.24163

C 0.34629 -0.02957
1.81751

H 0.50713 -0.19674
2.86207

H 0.41805 1.00954
1.57256

C -1.15811 -0.45378
1.60263

H -1.69019 0.13306
2.32193

H -1.23925 -1.49861
1.81862

C -1.6368 -1.25653 -
0.78055

H -2.27403 -2.07691 -
0.52403

H -1.89911 -0.85136 -
1.7355

C -0.28628 -1.91709 -
0.83241

H -0.12022 -2.32706
0.14189

H -0.42554 -2.68241 -
1.56712

C 1.03339 -1.27958 -
1.22358

H 1.65295 -2.14687 -
1.31774

H 0.94039 -0.7651 -
2.15715

N 1.79987 -0.4185 -
0.29204

H 2.73681 -0.65992 -
0.54477

1-azabicyclo[4.3.3]dodecan-2-one
The number of imaginary frequencies: 0
Total Energy (B3LYP/6-31G*)= -
560.58172753
ZPE= -560.287041
Enthalpy= -560.274841

N 0.58545 0.75154
0.86174

C 1.71539 0.32954
0.20676

C 2.0561 -1.15378 0.2463

H 2.98249 -1.23669 -
0.32614

H 2.27506 -1.51172
1.25936

C 0.93787 -2.02143 -
0.40669

H 1.43826 -2.8479 -
0.92418

H 0.33681 -2.49844
0.37254

C 0.01111 -1.30791 -
1.43311

H -0.19837 -2.04371 -2.21798

H 0.58687 -0.52061 -1.93232

C -1.39014 -0.73444 -0.98023

H -2.10244 -1.24003 -1.64569

C -0.00797 2.00809 0.39372

C -0.5696 1.86102 -1.02942

H -1.00334 2.82777 -1.3215

H 0.28457 1.70555 -1.69468

C -1.63889 0.77813 -1.29626

H -1.85544 0.8486 -2.37095

H -2.57735 1.06529 -0.79887

C -1.90333 -1.15059 0.42704

H -2.99982 -1.17723 0.36486

H -1.60875 -2.18752 0.63085

C -1.57375 -0.27368 1.6451

H -2.11206 0.67786 1.55733

H -1.98584 -0.75677 2.54191

C -0.09545 0.01835 1.925

H -0.02803 0.60553 2.85426

H 0.44476 -0.91148 2.11003

H 0.75997 2.78747 0.39464

H -0.78422 2.30498 1.10545

O 2.35009 1.09107 -0.52255

1-azabicyclo[4.3.3]dodecan-9-one
The number of imaginary frequencies: 0
Total Energy (B3LYP/6-31G*)= -560.56902632
ZPE= -560.275236
Enthalpy= -560.262784

N -0.59816 -1.12485 -0.31735

C 0.49517 -2.08473 -0.16122

H 0.19976 -2.8482 0.56938

H 0.56687 -2.60949 -1.1208

C 1.89485 -1.55044 0.20635

H 1.98959 -1.43017 1.2928

H 2.60505 -2.34588 -0.0588

C 2.31371 -0.24138 -0.48085

H 3.40539 -0.26492 -0.59565

H 1.91763 -0.20702 -1.5042

C 1.99816 1.07716 0.25586

H 2.6395 1.83519 -0.21491

H 2.36504 0.9928 1.28998

C 0.56934 1.69873 0.28875

H 0.79031 2.75564 0.49801

C -1.38146 -0.69074
0.74029

C -0.61081 -0.13903
1.91493

H -1.24241 -0.14499
2.80819

H 0.30685 -0.68919
2.13365

C -0.29003 1.33798
1.54107

H 0.22621 1.77482
2.40658

H -1.24908 1.86549
1.46226

C -0.10106 1.78295 -
1.1341

H -0.51147 2.79747 -
1.22309

H 0.71716 1.74692 -
1.86749

C -1.24403 0.86316 -
1.62913

H -2.14646 1.05644 -
1.04396

H -1.48139 1.17057 -
2.65721

C -0.99994 -0.65298 -
1.64389

H -1.90566 -1.16382 -
1.99998

H -0.18838 -0.9029 -
2.33528

O -2.58508 -0.48986
0.62421

1-azabicyclo[4.3.3]dodecan-3-one
The number of imaginary frequencies: 0
Total Energy (B3LYP/6-31G*)= -
560.55686645
ZPE=-560.264199
Enthalpy=-560.251578

N 0.34309 -0.8798 -0.4157

C 1.73533 -1.10397 -
0.24132

H 1.89773 -1.90784
0.44594

H 2.24126 -1.33272 -
1.15599

C 2.19856 0.22534
0.21856

C 1.60255 1.27663 -
0.73356

H 2.3743 1.99549 -
0.91392

H 1.34593 0.75851 -
1.63389

C 0.3535 2.10284 -
0.29984

H 0.16683 2.73441 -
1.14315

H 0.65213 2.66865
0.55782

C -1.08113 1.49678
0.08272

H -1.70771 2.36316 0.124

C -0.25066 -1.30017
0.83751

H 0.21688 -2.2176
1.12842

H -1.30631 -1.45255
0.75212

C -0.02431 -0.2272
1.82819

H -0.21424 -0.58294
2.81929

H 0.99038 0.10556
1.76054

C -1.03224 0.90507
1.54016

H -0.77882 1.7011
2.20875

H -2.01535 0.53682
1.74701

C -1.8688 0.51282 -1.0041

H -2.90544 0.61422 -
0.75916

H -1.65387 0.9151 -
1.97202

C -1.68767 -1.0849 -
1.07333

H -1.94986 -1.47322 -
0.11137

H -2.34727 -1.47227 -
1.82151

C -0.3001 -1.55212 -
1.39156

H -0.19954 -2.61501 -
1.32036

H 0.03026 -1.25867 -
2.36606

O 2.99053 0.44946
1.17046

1-azabicyclo[4.3.3]dodecan-4-one
The number of imaginary frequencies: 0
Total Energy (B3LYP/6-31G*)= -
560.55977731
ZPE=-560.266952
Enthalpy=-560.254458

N 0.13307 0.84165
0.50607

C 1.36254 1.48787
0.15671

H 1.25326 2.5441
0.02506

H 2.0895 1.31269
0.92205

C 1.79716 0.75911 -
1.05743

H 1.17199 0.90246 -
1.91388

H 2.7932 1.04175 -
1.32746

C 1.69686 -0.65932 -
0.51302

C 0.63667 -1.70843 -
0.91479

H 1.01328 -2.62704 -
0.51578

H 0.60213 -1.73605 -
1.98388

C -0.89826 -1.59071 -
0.47035

H -1.30093 -2.5581 -
0.68694

C -0.95111 1.47758 -
0.19979

H -0.7682 2.53162 -
0.22076

H -1.89441 1.29298
0.27033

C -1.00267 0.87996 -
1.55531

H -1.64869 1.45303 -
2.1871

H -0.01717 0.86828 -
1.97191

C -1.60079 -0.55925 -
1.44664

H -1.54726 -0.96554 -
2.43505

H -2.61817 -0.48052 -
1.12472

C -1.30617 -1.36232
1.11913

H -2.30158 -1.7457
1.20327

H -0.6146 -1.9441
1.69199

C -1.43609 0.08983
1.79697

H -2.15519 0.64393
1.23061

H -1.77434 -0.03232
2.80473

C -0.16503 0.87779 1.82821

H -0.30211 1.87002 2.20449

H 0.59122 0.40416 2.41868

O 2.55263 -1.00715 0.34152

1-azabicyclo[4.3.3]dodecan-5-one
The number of imaginary frequencies: 0
Total Energy (B3LYP/6-31G*)= -560.56282261
ZPE=-560.269812
Enthalpy=-560.257410

N -0.01198 -1.25805 0.4156

C 1.36702 -1.73336 0.42617

H 1.65876 -2.02261 1.44535

H 1.43343 -2.64799 -0.17885

C 2.3722 -0.69004 -0.12563

H 2.75784 -0.05497 0.67964

H 3.23677 -1.20847 -0.5599

C 1.72796 0.24115 -1.17688

H 2.52596 0.78952 -1.69202

H 1.17885 -0.33393 -1.92661

C 0.8708 1.29835 -0.47507

C -0.65876 1.42543 -0.59741

H -0.77258 2.43644 -1.01831

C -0.65584 -0.89722 1.67668

H -0.14289 -1.39167 2.51626

H -1.68145 -1.29309 1.67578

C -0.6842 0.61736 1.94059

H -1.24849 0.7942 2.86749

H 0.33727 0.96032 2.1363

C -1.28683 1.51033 0.83599

H -1.18007 2.54295 1.182

H -2.36632 1.32838 0.74887

C -1.41634 0.49669 -1.58299

H -2.28305 1.06541 -1.94583

H -0.79493 0.32416 -2.47208

C -1.95723 -0.84738 -1.06783

H -2.67703 -0.66372 -0.26036

H -2.53501 -1.32594 -1.87052

C -0.89221 -1.83659 -0.59135

H -1.38834 -2.76011 -0.22984

H -0.27628 -2.13072 -1.44828

O 1.44775 2.11122 0.23351

1-azabicyclo[4.3.3]dodecan-7-one
The number of imaginary frequencies: 0
Total Energy (B3LYP/6-31G*)= -560.56098819

ZPE=-560.268366
Enthalpy=-560.255873

N 0.93902 -0.85182 -
0.63098

C 1.78811 0.14136 -
1.25907

H 2.83967 -0.20132 -
1.29853

H 1.46013 0.25618 -
2.29911

C 1.74153 1.52776 -
0.58819

H 2.22479 1.49224
0.39648

H 2.37067 2.19409 -
1.19641

C 0.3343 2.15102 -
0.47219

H 0.46298 3.24184 -
0.48678

H -0.246 1.91577 -
1.37219

C -0.54011 1.8727
0.77495

H -1.35561 2.60175
0.72153

H 0.02998 2.12957
1.67878

C -1.2088 0.47717
1.01678

H -2.15714 0.71693
1.52553

C 1.4184 -1.47699
0.58844

H 2.51655 -1.58497
0.55461

H 1.01303 -2.49744
0.63839

C 1.02714 -0.71992
1.86702

H 1.36533 -1.30554
2.73439

H 1.57909 0.22509
1.91393

C -0.47445 -0.43878
2.03283

H -0.59971 0.03818
3.01443

H -1.01588 -1.39178
2.11109

C -1.72159 -0.13168 -
0.29907

C -1.41709 -1.57593 -
0.68749

C -0.04738 -1.58307 -
1.40355

H 0.25592 -2.62602 -
1.61133

H -0.16665 -1.08285 -
2.36918

H -1.38873 -2.23593
0.18561

H -2.20248 -1.92894 -
1.36341

O -2.36992 0.56802 -
1.06047

1-azabicyclo[4.3.3]dodecan-8-one
The number of imaginary frequencies: 0
Total Energy (B3LYP/6-31G*)= -
560.554706
ZPE=-560.262255
Enthalpy=-560.249530
N 0.11068 -1.32893
0.0375

C 1.43274 -1.70918 -
0.41232

H 1.80948 -2.5781
0.15865

H 1.35548 -2.03434 -
1.45729

C 2.46525 -0.57144 -
0.33673

H 2.71297 -0.34968
0.70871

H 3.39611 -0.94693 -
0.78611

C 2.0431 0.71928 -
1.06576

H 2.96196 1.20949 -
1.41454

H 1.50147 0.45094 -
1.98404

C 1.26943 1.82383 -
0.30314

H 1.37461 2.72065 -
0.92942

H 1.81972 2.05822
0.61971

C -0.24974 1.73443
0.05127

H -0.55869 2.78788
0.06383

C -0.14086 -1.28978
1.46919

H 0.45044 -2.07235
1.97468

H -1.1964 -1.54091
1.63979

C 0.1571 0.06906
2.11723

H -0.12195 0.00635
3.17913

H 1.23738 0.25451
2.10511

C -0.5751 1.27392
1.50428

H -0.35586 2.13055
2.15593

H -1.65993 1.11761
1.58332

C -1.14911 1.11647 -
1.08008

H -1.94391 1.82788 -
1.32295

H -0.5481 0.98021 -
1.98803

C -1.87766 -0.19247 -
0.78832

C -1.04706 -1.47945 -
0.80985

H -1.70951 -2.31333 -
0.52235

H -0.71749 -1.65398 -
1.8422

O -3.06889 -0.21502 -
0.53488

1-azabicyclo[4.3.3]dodecane N-oxide
The number of imaginary frequencies: 0
Total Energy (B3LYP/6-31G*)= -
561.66828587
ZPE= -561.35115
Enthalpy= -561.338551

N 0.40679 -1.27086 -
0.05346

C 1.83246 -1.13866 -
0.25423

H 2.39188 -1.84521
0.38658

H 2.06027 -1.4182 -
1.29071

C 2.35452 0.29 -0.01702

H 2.35545 0.52346
1.05516

H 3.40984 0.31516 -
0.32642

C 1.57118 1.36919 -
0.79145

H 2.27703 2.17634 -
1.03055

H 1.26307 0.96079 -
1.76375

C 0.36878 2.07362 -0.116

H 0.21612 2.99005 -
0.70364

H 0.68691 2.42642
0.8765

C -1.05047 1.43197
0.02848

H -1.69801 2.32043
0.05674

C -0.06841 -1.48695
1.29817

H 0.66282 -2.0887
1.86652

H -0.98842 -2.08664
1.25747

C -0.34699 -0.18671
2.06502

H -0.72789 -0.44805
3.06388

H 0.59997 0.33816
2.23842

C -1.34869 0.78041
1.41252

H -1.47394 1.61011
2.12229

H -2.33552 0.29767
1.36407

C -1.56258 0.66008 -
1.23628

H -2.54507 1.07838 -
1.49246

H -0.91708 0.90467 -
2.09112

C -1.75255 -0.86685 -
1.18859

H -2.38858 -1.13411 -
0.33472

H -2.31736 -1.17643 -
2.07995

C -0.45636 -1.68445 -
1.13898

H -0.71388 -2.76253 -
1.09212

H 0.08954 -1.53569 -
2.07775

O 0.6837 -2.59907
0.04026

1-azabicyclo[4.3.3]dodecane N-oxide
The number of imaginary frequencies: 0
Total Energy (M06/6-311G+(d,p))= -
561.41002052
ZPE= -561.096465
Enthalpy= -561.083943

N 1.64025 -0.094 -
0.10182

C 1.38832 1.35137 -
0.27683

H 2.06804 1.81274
0.40875

H 1.6539 1.54597 -
1.29491

C 0.07516 2.13291 -
0.05455

H -0.28101 2.07203
0.9526

H 0.37167 3.13415 -0.288

C -1.06313 1.79787 -
0.99182

H -1.57284 2.71605 -
1.19686

H -0.64097 1.3921 -
1.88739

C -2.19779 0.92599 -
0.42756

H -2.88164 0.84903 -
1.24691

H -2.61047 1.48898
0.38338

C -2.09264 -0.50782
0.12336

H -3.11678 -0.79179
0.24746

C 1.34163 -0.42766
1.29732

H 2.07521 0.09758
1.87254

H 1.43749 -1.48355
1.44158

C -0.03841 0.07345
1.86501

H 0.05557 -0.04769
2.92396

H -0.12176 1.10564
1.59571

C -1.42472 -0.60472 1.537

H -2.10957 -0.10635
2.19085

H -1.34707 -1.6446
1.77683

C -1.54899 -1.51405 -
0.85273

H -2.0587 -2.42716 -
0.62617

H -1.79067 -1.1744 -
1.83818

C -0.10711 -1.93499 -
0.77061

H 0.03919 -2.29551
0.22615

H -0.04985 -2.72408 -1.491

C 1.11529 -1.08758 -
1.06823

H 1.87821 -1.83766 -
1.08361

H 1.01949 -0.61099 -
2.02142

O 2.97689 -0.20514 -
0.32685

Trimethylamine
The number of imaginary frequencies: 0
Total Energy (B3LYP/6-31G*)= -
174.47441505
ZPE= -174.353287
Enthalpy= -174.346933

N -0.23715 0.11869 -
0.00005

C 0.25311 0.81147 -
1.20029

H -0.10517 0.30823 -
2.07395

H -0.10173 1.82092 -
1.19941

H 1.32311 0.80955 -
1.20117

C 0.25035 -1.26812 -
0.00127

H -0.10652 -1.7724
0.87238

H -0.10794 -1.77137 -
0.87492

H 1.32034 -1.27005 -
0.00214

C 0.25508 0.81006 1.2002

H -0.09977 1.81951
1.20109

H -0.10179 0.30579
2.07385

H 1.32507 0.80813
1.19933

Trimethylamine
The number of imaginary frequencies: 0
Total Energy (B3LYP/6-31G**)= -
174.359219
ZPE= -174.365595
Enthalpy= -174.359219
N 0. 0.
0.37142

C 0. 1.38908 -
0.06099

H -0.88689 1.89889
0.33175

H 0.88689 1.89889
0.33175

H 0. 1.5053 -
1.16421

C -1.20298 -0.69454 -
0.06099

H -1.20104 -1.71751
0.33175

H -2.08793 -0.18138
0.33175

H -1.30363 -0.75265 -
1.16421

C 1.20298 -0.69454 -
0.06099

H 2.08793 -0.18138
0.33175

H 1.20104 -1.71751
0.33175

H 1.30363 -0.75265 -
1.16421

Trimethylamine
The number of imaginary frequencies: 0
Total Energy (M06/6-311G+(d,p))= -
174.38202143
ZPE= -174.262877
Enthalpy= -174.256485

N 0. 0. 0.37142

C 0. 1.38908 -0.06099

H -0.88689 1.89889
0.33175

H 0.88689 1.89889
0.33175

H 0. 1.5053 -1.16421

C -1.20298 -0.69454 -
0.06099

H -1.20104 -1.71751
0.33175

H -2.08793 -0.18138
0.33175

H -1.30363 -0.75265 -
1.16421

C 1.20298 -0.69454 -
0.06099

H 2.08793 -0.18138
0.33175

H 1.20104 -1.71751
0.33175

H 1.30363 -0.75265 -
1.16421

Trimethylamine
The number of imaginary frequencies: 0
Total Energy (B3PW91/6-31G*)= -
174.41045066
ZPE= -174.289118
Enthalpy= -174.282761

N -0.23715 0.11869 -
0.00005

C 0.25311 0.81147 -
1.20029

H -0.10517 0.30823 -
2.07395

H -0.10173 1.82092 -
1.19941

H 1.32311 0.80955 -
1.20117

C 0.25035 -1.26812 -
0.00127

H -0.10652 -1.7724
0.87238

H -0.10794 -1.77137 -
0.87492

H 1.32034 -1.27005 -
0.00214

C 0.25508 0.81006 1.2002

H -0.09977 1.81951
1.20109

H -0.10179 0.30579
2.07385

H 1.32507 0.80813
1.19933

Trimethylamine
The number of imaginary frequencies: 0
Total Energy (B3PW91/6-31G**)= -174.42143905
ZPE= -174.300694
Enthalpy= -174.294319

N -0.23715 0.11869 -0.00005

C 0.25311 0.81147 -1.20029

H -0.10517 0.30823 -2.07395

H -0.10173 1.82092 -1.19941

H 1.32311 0.80955 -1.20117

C 0.25035 -1.26812 -0.00127

H -0.10652 -1.7724 0.87238

H -0.10794 -1.77137 -0.87492

H 1.32034 -1.27005 -0.00214

C 0.25508 0.81006 1.2002

H -0.09977 1.81951 1.20109

H -0.10179 0.30579 2.07385

H 1.32507 0.80813 1.19933

Trimethylamine
The number of imaginary frequencies: 0
Total Energy (B3PW91/6-311G+(d,p))= -174.45968937
ZPE= -174.339866
Enthalpy= -174.333441

N -0.23715 0.11869 -0.00005

C 0.25311 0.81147 -1.20029

H -0.10517 0.30823 -2.07395

H -0.10173 1.82092 -1.19941

H 1.32311 0.80955 -1.20117

C 0.25035 -1.26812 -0.00127

H -0.10652 -1.7724 0.87238

H -0.10794 -1.77137 -0.87492

H 1.32034 -1.27005 -0.00214

C 0.25508 0.81006 1.2002

H -0.09977 1.81951 1.20109

H -0.10179 0.30579 2.07385

H 1.32507 0.80813 1.19933

Trimethylamine
The number of imaginary frequencies: 0
Total Energy (Hartree Fock 6-31G*)= -173.26929918
ZPE= -173.139801
Enthalpy= -173.13364

N -0.55556 2.19935 -0.11514

C -0.0653 2.89239 1.08495

H -0.42357 3.90063 1.08595

H -0.42016 2.38691 1.95872

H 1.0047 2.8941 1.08374

C -0.06334 0.8142 -0.11652

H -0.41821 0.30872 0.75724

H -0.4202 0.30974 -
0.99006

H 1.00665 0.81591 -
0.11773

C -0.06803 2.8938 -
1.31554

H -0.42489 2.38934 -
2.18908

H -0.42631 3.90203 -
1.31454

H 1.00197 2.89551 -
1.31676

Trimethylamine N-oxide
The number of imaginary frequencies: 0
Total Energy (B3LYP/6-31G*)= -
249.61561382
ZPE= -249.489573
Enthalpy= -249.482621

N 0. 0.
0.08377

C 0. 1.41754 -
0.41816

H -0.88839 1.89255 -
0.00215

H 0.88839 1.89255 -
0.00215

H 0. 1.4678 -
1.51318

C -1.22763 -0.70877 -
0.41816

H -1.1948 -1.71564 -
0.00215

H -2.08319 -0.1769 -
0.00215

H -1.27115 -0.7339 -
1.51318

C 1.22763 -0.70877 -
0.41816

H 2.08319 -0.1769 -
0.00215

H 1.1948 -1.71564 -
0.00215

H 1.27115 -0.7339 -
1.51318

O 0. 0.
1.44003

Trimethylamine N-oxide
The number of imaginary frequencies: 0
Total Energy (B3LYP/6-31G**)= -
249.6275473
ZPE= -249.502133
Enthalpy= -249.495143

N 0. 0.

0.08312

C 0. 1.41754 -

0.41881

H -0.88839 1.89255 -0.0028

H 0.88839 1.89255 -0.0028

H 0. 1.4678 -

1.51383

C -1.22763 -0.70877 -

0.41881

H -1.1948 -1.71564 -0.0028

H -2.08319 -0.1769 -0.0028

H -1.27115 -0.7339 -

1.51383

C 1.22763 -0.70877 -

0.41881

H 2.08319 -0.1769 -0.0028

H 1.1948 -1.71564 -0.0028

H 1.27115 -0.7339 -
1.51383

O 0. 0.
1.43938

Trimethylamine N-oxide
The number of imaginary frequencies: 0
Total Energy (M06/6-311G+(d,p))= -
249.52070728
ZPE= -249.396733
Enthalpy= -249.389724

N 0. 0.
0.08312

C 0. 1.41754 -
0.41881

H -0.88839 1.89255 -
0.0028

H 0.88839 1.89255 -
0.0028

H 0. 1.4678 -
1.51383

C -1.22763 -0.70877 -
0.41881

H -1.1948 -1.71564 -
0.0028

H -2.08319 -0.1769 -
0.0028

H -1.27115 -0.7339 -
1.51383

C 1.22763 -0.70877 -
0.41881

H 2.08319 -0.1769 -0.0028

H 1.1948 -1.71564 -0.0028

H 1.27115 -0.7339 -
1.51383

O 0. 0.
1.43938

Trimethylamine N-oxide
The number of imaginary frequencies: 0
Total Energy (B3PW91/6-31G*)= -
249.52323117
ZPE= -249.396878
Enthalpy= -249.389954

N 0. 0. 0.08377

C 0. 1.41754 -0.41816

H -0.88839 1.89255 -
0.00215

H 0.88839 1.89255 -
0.00215

H 0. 1.4678 -
1.51318

C -1.22763 -0.70877 -
0.41816

H -1.1948 -1.71564 -
0.00215

H -2.08319 -0.1769 -
0.00215

H -1.27115 -0.7339 -
1.51318

C 1.22763 -0.70877 -
0.41816

H 2.08319 -0.1769 -
0.00215

H 1.1948 -1.71564 -
0.00215

H 1.27115 -0.7339 -
1.51318

O 0. 0.
1.44003

Trimethylamine N-oxide
The number of imaginary frequencies: 0
Total Energy (B3PW91/6-31G**)= -
249.53436118
ZPE= -249.408677
Enthalpy= -249.401712

N 0. 0. 0.08377

C 0. 1.41754 -0.41816

H -0.88839 1.89255 -
0.00215

H 0.88839 1.89255 -
0.00215

H 0. 1.4678 -
1.51318

C -1.22763 -0.70877 -
0.41816

H -1.1948 -1.71564 -
0.00215

H -2.08319 -0.1769 -
0.00215

H -1.27115 -0.7339 -
1.51318

C 1.22763 -0.70877 -
0.41816

H 2.08319 -0.1769 -
0.00215

H 1.1948 -1.71564 -
0.00215

H 1.27115 -0.7339 -
1.51318

O 0. 0.
1.44003

Trimethylamine N-oxide
The number of imaginary frequencies: 0
Total Energy (B3PW91/6-311G+(d,p))= -
249.60267295
ZPE= -249.477639
Enthalpy= -249.470642

N 0. 0.
0.08377

C 0. 1.41754 -
0.41816

H -0.88839 1.89255 -
0.00215

H 0.88839 1.89255 -
0.00215

H 0. 1.4678 -
1.51318

C -1.22763 -0.70877 -
0.41816

H -1.1948 -1.71564 -
0.00215

H -2.08319 -0.1769 -
0.00215

H -1.27115 -0.7339 -
1.51318

C 1.22763 -0.70877 -
0.41816

H 2.08319 -0.1769 -
0.00215

H 1.1948 -1.71564 -
0.00215

H 1.27115 -0.7339 -
1.51318

O 0. 0.
1.44003

Trimethylamine N-oxide
The number of imaginary frequencies: 0
Total Energy (Hartree Fock 6-31G*)= -
248.04640634
ZPE= -247.910609
Enthalpy= -247.904082

N -0.55556 2.19935 -
0.11514

C -0.0653 2.89239
1.08495

H -0.42357 3.90063
1.08595

H -0.42016 2.38691
1.95872

H 1.0047 2.8941
1.08374

C -0.06334 0.8142 -
0.11652

H -0.41821 0.30872
0.75724

H -0.4202 0.30974 -
0.99006

H 1.00665 0.81591 -
0.11773

C -0.06803 2.8938 -1.31554

H -0.42489 2.38934 -2.18908

H -0.42631 3.90203 -1.31454

H 1.00197 2.89551 -1.31676

O -1.91555 2.19718 -0.11359

Trimethylaminium
The number of imaginary frequencies: 0
Total Energy (B3LYP/6-31G*)= -174.85106876
ZPE= -174.714195
Enthalpy= -174.707684

N 0. 0.00006 0.42913

C 0. 1.38592 -0.06109

H -0.87365 1.89038 0.29551

H 0.87366 1.89037 0.2955

H 0. 1.38576 -1.13109

C -1.20025 -0.69297 -0.06077

H -1.20025 -1.70172 0.29605

H -2.0739 -0.18851 0.29582

H -1.20025 -0.69313 -1.13077

C 1.20025 -0.69298 -0.06077

H 2.0739 -0.18852 0.29582

H 1.20025 -1.70173 0.29605

H 1.20025 -0.69314 -1.13077

H 0. 0.00022 1.42913

Trimethylaminium
The number of imaginary frequencies: 0
Total Energy (M06/6-311G+(d,p))= -174.74831058
ZPE= -174.614346
Enthalpy= -174.607656

N 0. 0. -0.38

C 0. 1.38593 0.11

H 0.87365 1.89033 -0.24667

H -0.87365 1.89033 -0.24667

H 0. 1.38593 1.18

C 1.20025 -0.69296 0.11

H 1.20025 -1.70177 -0.24667

H 2.0739 -0.18856 -0.24667

H 1.20025 -0.69296 1.18

C -1.20025 -0.69296 0.11

H -2.0739 -0.18856 -0.24667

H -1.20025 -1.70177 -0.24667

H -1.20025 -0.69296 1.18

H 0. 0. -1.38

Methanamine
The number of imaginary frequencies: 0
Total Energy (B3LYP/6-31G*)= -249.63811327
ZPE= -249.512681
Enthalpy= -249.505072

C 1.93077 -0.00053 -0.19893

H 2.78142 -0.00087 0.45015

H 1.95289 -0.87417 -0.81633

H 1.95352 0.87314 -0.81625

C -1.13364 -1.22098 -0.01552

H -1.97665 -1.22922 -0.67443

H -0.52317 -2.07871 -0.20664

H -1.47351 -1.24395 0.9988

O 0.73398 -0.00014 0.58374

N -0.34722 0.00029 -0.24125

C -1.13276 1.2221 -0.01541

H -0.52168 2.07941 -0.20645

H -1.97577 1.23101 -0.67432

H -1.47262 1.24522 0.99891

Methanamine
The number of imaginary frequencies: 0
Total Energy (M06/6-311G+(d,p))= -249.53609711
ZPE= -249.412608
Enthalpy= -249.405058

C 1.9266 0. -0.19527

H 2.73029 0. 0.54658

H 2.00833 -0.89282 -0.82931

H 2.00833 0.89282 -0.82931

C -1.13036 -1.20925 -0.02138

H -1.99892 -1.23176 -0.687

H -0.50884 -2.08505 -0.22874

H -1.47537 -1.2607 1.02592

O 0.71379 0. 0.54702

N -0.35518 0. -0.29376

C -1.13036 1.20925 -0.02138

H -0.50884 2.08505 -0.22874

H -1.99892 1.23176 -0.687

H -1.47537 1.2607 1.02592

Ethanamine
The number of imaginary frequencies: 0
Total Energy (B3LYP/6-31G*)= -249.68300495
ZPE= -249.55625
Enthalpy= -249.548583

C -0.90757 0.75395 -0.13564

H -1.2254 1.71597 -0.47974

H -1.46972 -0.00717 -0.63522

H -1.06855 0.68155 0.9197

C 0.90838 -0.70347 0.04137

C 2.40657 -0.88841 -0.26326

H 0.7474 -0.77587 1.09671

H 0.34622 -1.46459 -0.45821

H 2.56755 -0.81601 -1.3186

H 2.96873 -0.12729
0.23632

O 0.48361 0.58222 -
0.41851

N 2.84322 -2.21006
0.20948

H 3.81607 -2.33015
0.01167

H 2.69278 -2.27772
1.19579

Ethanamine
The number of imaginary frequencies: 0
Total Energy (M06/6-311G+(d,p))= -
249.458677
ZPE= -249.466382
Enthalpy= -249.458677

C 2.39094 -0.19573
0.01077

H 3.19214 0.54169 -
0.08895

H 2.50576 -0.71973
0.97376

H 2.48809 -0.93913 -
0.79784

C 0.0434 -0.34752
0.05415

C -1.21436 0.51131 -
0.01284

H 0.03787 -1.09489 -0.761

H 0.06151 -0.90441
1.00469

H -1.21426 1.19365
0.84436

H -1.16269 1.13654 -
0.9206

O 1.16821 0.50193 -
0.06356

N -2.40165 -0.34534
0.07822

H -3.23306 0.23069
0.19166

H -2.52928 -0.85084 -
0.79763

Dimethylaminomethanol
The number of imaginary frequencies: 0
Total Energy (B3LYP/6-31G*)= -
249.68172356
ZPE= -249.555788
Enthalpy= -249.548324

C -0.54804 1.39222 0.0741

H 0.35805 1.92515 -
0.22934

H -1.39713 1.85627 -
0.43704

H -0.69544 1.53361
1.16556

C -1.6404 -0.75027 -
0.02821

H -1.54883 -1.76248 -
0.43514

H -1.86998 -0.83816
1.05329

H -2.4971 -0.26403 -
0.50581

N -0.43238 0.00604 -
0.32168

C 0.737 -0.64005
0.25148

H 0.73019 -1.68859 -
0.03969

H 0.73132 -0.59029
1.35913

O 1.96162 -0.10356 -
0.22974

H 2.23116 0.60334
0.37443

Dimethylaminomethanol
The number of imaginary frequencies: 0
Total Energy (M06/6-311G+(d,p))= -
249.58819455
ZPE= -249.464461

Enthalpy= -249.457371

C -0.54804 1.39222
0.0741

H 0.35805 1.92515 -
0.22934

H -1.39713 1.85627 -
0.43704

H -0.69544 1.53361
1.16556

C -1.6404 -0.75027 -
0.02821

H -1.54883 -1.76248 -
0.43514

H -1.86998 -0.83816
1.05329

H -2.4971 -0.26403 -
0.50581

N -0.43238 0.00604 -
0.32168

C 0.737 -0.64005
0.25148

H 0.73019 -1.68859 -
0.03969

H 0.73132 -0.59029
1.35913

O 1.96162 -0.10356 -
0.22974

H 2.23116 0.60334
0.37443

Trimethylaminomethanol
The number of imaginary frequencies: 0
Total Energy (B3LYP/6-31G*)= -
250.00872901
ZPE= -249.869457
Enthalpy= -249.861997

N 0. 0.
0.08312

C 0. 1.41754 -
0.41881

H -0.88839 1.89255 -
0.0028

H 0.88839 1.89255 -
0.0028

H 0. 1.4678 -
1.51383

C -1.22763 -0.70877 -
0.41881

H -1.1948 -1.71564 -
0.0028

H -2.08319 -0.1769 -
0.0028

H -1.27115 -0.7339 -
1.51383

C 1.22763 -0.70877 -
0.41881

H 2.08319 -0.1769 -0.0028

H 1.1948 -1.71564 -0.0028

H 1.27115 -0.7339 -
1.51383

O 0. 0.
1.43938

H 0.9051 0.
1.75938

Trimethylaminomethanol
The number of imaginary frequencies: 0
Total Energy (M06/6-311G+(d,p))= -
249.90268818
ZPE= -249.765408
Enthalpy= -249.758011

N 0.00359 0.00003 -
0.00407

C 0.67148 1.25816 -
0.48684

H 0.14803 2.1059 -
0.04552

H 1.71605 1.2419 -
0.16952

H 0.614 1.28728 -
1.57638

C -1.45474 -0.01962 -
0.37823

H -1.89576 -0.92111
0.04589

H -1.92025 0.86836
0.04817

H -1.52719 -0.0194 -
1.46624

C 0.70582 -1.23898 -
0.48779

H 1.74934 -1.19445 -
0.16968

H 0.20563 -2.10114 -
0.04753

H 0.65008 -1.26848 -
1.5774

O -0.02599 -0.00107
1.42316

H 0.90752 0.01216
1.71855

Nitromethane
The number of imaginary frequencies: 0
Total Energy (B3LYP/6-31G*)= -
245.00932726
ZPE= -244.959092
Enthalpy= -244.953835

N 2.33004 0.25514 0.

O 3.56716 0.28984
0.00158

O 1.68144 1.30916 -
0.00157

C 1.63103 -1.03803 0.

H 1.46273 -1.35249 -
1.00881

H 2.22932 -1.76688
0.50568

H 0.69222 -0.936
0.50311

Nitromethane
The number of imaginary frequencies: 1 (-
29.36 cm^{-1})
Total Energy (M06/6-311G+(d,p))= -
244.94960552
ZPE= -244.900022
Enthalpy= -244.895614

N 0.15385 0.00001 -
0.00178

O 0.7726 1.07182 -
0.00802

O 0.77264 -1.07177 -
0.00774

C -1.31608 -0.00001
0.01272

H -1.66278 0.00011
1.02499

H -1.67772 0.87357 -
0.48826

H -1.67769 -0.87373 -
0.48803

Methylnitrite
The number of imaginary frequencies: 0
Total Energy (B3LYP/6-31G*)= -
245.00795817
ZPE= -244.959108
Enthalpy= -244.953786

C -0.87908 -0.57341 0.011

H -0.22485 -1.41968 -
0.01543

H -0.69587 0.04584 -
0.84215

H -1.89542 -0.90773 -
0.0026

O -0.64001 0.17681
1.2047

N -0.82548 1.40039
1.21414

O -1.21785 1.9747
0.19049

Methylnitrite
The number of imaginary frequencies: 0
Total Energy (M06/6-311G+(d,p))= -
244.94401884
ZPE= -244.895496
Enthalpy= -244.890279

C -1.29687 0.42144 0.

H -2.31666 0.0373 -
0.00035

H -1.10955 1.02791 -
0.89114

H -1.11006 1.02756
0.89149

O -0.46824 -0.75474 -
0.00001

N 0.90934 -0.51993
0.00001

O 1.21225 0.63201 -
0.00001

Pyrrolidine
The number of imaginary frequencies: 0
Total Energy (B3LYP/6-31G*)= -
212.58201691
ZPE= -212.451628
Enthalpy= -212.445712

C -0.35518 1.14266
0.75224

C 1.66599 0.92763 -
0.11519

C 1.21815 2.35076 -
0.54335

C -0.27836 2.31052 -
0.19821

H -1.3606 0.83838
0.95585

H 0.12138 1.34396
1.68886

H 2.00407 0.96391
0.89935

H 2.45269 0.54167 -
0.72922

H 1.82578 3.03812
0.00731

H 1.2855 2.59188 -
1.58365

H -0.66829 3.25028
0.13301

H -0.82826 1.99974 -
1.06188

N 0.4318 0.15228 -
0.06269

H 0.02441 -0.0232 -
0.95893

Pyrrolidine
The number of imaginary frequencies: 0
Total Energy (M06/6-311G+(d,p))= -
212.47328852
ZPE= -212.344771
Enthalpy= -212.33877

C -1.15663 -0.45327
0.19297

C 1.15639 -0.45362
0.19329

C 0.77798 1.0303 -
0.07053

C -0.77756 1.03066 -
0.07012

H -2.05637 -0.7771 -
0.33946

H -1.33544 -0.61146
1.26475

H 1.33453 -0.61147
1.26524

H 2.05631 -0.77799 -
0.33851

H 1.19788 1.70083
0.6869

H 1.1653 1.36132 -
1.04085

H -1.19669 1.70085
0.68805

H -1.16528 1.36262 -
1.03996

N -0.00017 -1.27389 -
0.20026

H -0.00003 -1.35474 -
1.21799

Pyrrolidine N-oxide
The number of imaginary frequencies: 0
Total Energy (B3LYP/6-31G*)= -
287.7234805
ZPE= -287.588302
Enthalpy= -287.581788

C -0.00033 1.14666
0.49444

C 0.22374 -1.05371
0.76688

C 0.96197 -0.79536 -
0.52621

C 1.08575 0.74368 -
0.53982

H -0.47516 2.07704
0.26244

H 0.45616 1.22377
1.45911

H 0.81878 -0.84826
1.63211

H -0.13466 -2.05837
0.85127

H 1.89161 -1.30274 -
0.67863

H 0.2804 -1.09447 -
1.29491

H 2.00673 1.16062 -
0.18931

H 0.93355 1.05243 -
1.55294

N -0.85181 -0.02712
0.58429

O -1.56281 -0.24663 -
0.55408

H -1.50044 0.02115
1.34386

Pyrrolidine N-oxide
The number of imaginary frequencies: 0
Total Energy (M06/6-311G+(d,p))= -
287.61263562
ZPE= -287.478669
Enthalpy= -287.472253

C 0.01206 -1.18031
0.52425

C 0.01184 1.18049
0.52397

C -1.21089 0.77991 -
0.31338

C -1.21052 -0.78023 -
0.31346

H 0.54995 -2.05773
0.1677

H -0.22877 -1.28622
1.5907

H -0.22873 1.2867
1.59044

H 0.54951 2.05786 0.167

H -2.12812 1.19622
0.11317

H -1.10535 1.15647 -
1.33143

H -2.12776 -1.19704
0.11264

H -1.1044 -1.15665 -
1.3315

N 0.94195 0.00013
0.34004

O 1.49591 0.00001 -
0.88636

H 1.64784 0.00032
1.10359

Pyrroline
The number of imaginary frequencies: 0

Total Energy (B3LYP/6-31G*)= -211.37224827
ZPE= -211.266433
Enthalpy= -211.260945

C -0.49599 1.00851 0.46461

C 1.66072 0.9327 -0.09942

C 1.21845 2.35201 -0.52028

C -0.28937 2.31892 -0.27064

H -1.27831 0.80132 1.16457

H 2.05448 0.96858 0.89485

H 2.40803 0.54681 -0.76087

H 1.80627 3.01176 0.08312

H 1.3273 2.62831 -1.54824

H -0.64141 3.22178 0.18302

H -0.80971 2.17064 -1.19377

N 0.43286 0.14649 0.05661

Pyrroline
The number of imaginary frequencies: 0
Total Energy (M06/6-311G+(d,p))= -211.26189917
ZPE= -211.157429
Enthalpy= -211.151965

C 0.79886 -0.91542 -0.04069

C -1.23392 0.0008 0.07707

C -0.2629 1.20077 -0.0989

C 1.13211 0.55755 0.07986

H 1.56696 -1.68896 -0.09765

H -2.03854 -0.01318 -0.66631

H -1.71638 -0.00663 1.06376

H -0.35942 1.62428 -1.10418

H -0.45414 2.00652 0.61532

H 1.86709 0.88284 -0.66596

H 1.57371 0.7638 1.06555

N -0.43489 -1.23298 -0.04494

Methyl Pyrrolidine
The number of imaginary frequencies: 0
Total Energy (B3LYP/6-31G*)= -251.88956837
ZPE= -251.731361
Enthalpy= -251.724164

C -1.15663 -0.45327 0.19297

C 1.15639 -0.45362 0.19329

C 0.77798 1.0303 -0.07053

C -0.77756 1.03066 -0.07012

H -2.05637 -0.7771 -0.33946

H -1.33544 -0.61146 1.26475

H 1.33453 -0.61147 1.26524

H 2.05631 -0.77799 -
0.33851

H 1.19788 1.70083
0.6869

H 1.1653 1.36132 -
1.04085

H -1.19669 1.70085
0.68805

H -1.16528 1.36262 -
1.03996

N -0.00017 -1.27389 -
0.20026

C 0.00003 -1.3903 -
1.66564

H 0.87482 -1.91947 -
1.98127

H -0.87248 -1.92325 -
1.98122

H -0.0021 -0.41291 -
2.10107

H -0.43417 -1.30864 -
1.55831

H 0.45527 -2.0595 -
0.22783

H -2.20509 -1.19792
0.02894

H -1.11008 -1.16732
1.4062

H -2.20542 1.19716
0.02825

H -1.11112 1.16732
1.40608

N 0.84465 0.00011 -
0.54566

C 1.76509 0. 0.59063

H 2.40898 -0.88422
0.53277

H 2.40905 0.88419
0.53288

H 1.27997 -0.00004
1.58496

Methyl Pyrrolidine
The number of imaginary frequencies: 0
Total Energy (M06/6-311G+(d,p))= -
251.7568498
ZPE= -251.601035
Enthalpy= -251.593775

C -0.07138 1.15044 -
0.53372

C -0.07129 -1.15029 -
0.53401

C -1.2628 -0.77695 0.3945

C -1.26312 0.77669
0.39438

H 0.45507 2.05955 -
0.22703

H -0.43402 1.30923 -
1.55803

Methyl Pyrrolidine N-oxide
The number of imaginary frequencies: 0
Total Energy (B3LYP/6-31G*)= -
327.03549275
ZPE= -326.872485
Enthalpy= -326.864715

C -1.15663 -0.45327
0.19297

C 1.15639 -0.45362
0.19329

C 0.77798 1.0303 -
0.07053

C -0.77756 1.03066 -
0.07012

H -2.05637 -0.7771 -
0.33946

H -1.33544 -0.61146
1.26475

H 1.33453 -0.61147
1.26524

H 2.05631 -0.77799 -
0.33851

H 1.19788 1.70083
0.6869

H 1.1653 1.36132 -
1.04085

H -1.19669 1.70085
0.68805

H -1.16528 1.36262 -
1.03996

N -0.00017 -1.27389 -
0.20026

C 0.00003 -1.3903 -
1.66564

H 0.87482 -1.91947 -
1.98127

H -0.87248 -1.92325 -
1.98122

H -0.0021 -0.41291 -
2.10107

O -0.00044 -2.50637
0.37472

Methyl Pyrrolidine N-oxide
The number of imaginary frequencies: 0
Total Energy (M06/6-311G+(d,p))= -
326.90051885
ZPE= -326.739215
Enthalpy= -326.731606

C -0.20642 1.18006 -
0.39819

C -0.20641 -1.17988 -
0.39867

C -1.60313 -0.78221
0.08929

C -1.60323 0.78213
0.08935

H 0.22053 2.074
0.05958

H -0.14015 1.27502 -
1.4834

H -0.14032 -1.27427 -
1.48394

H 0.22064 -2.07405
0.05855

H -2.37717 -1.19037 -
0.56706

H -1.80187 -1.16923
1.09398

H -2.37711 1.1902 -
0.56724

H -1.80238 1.16905 1.094

N 0.71326 0.00002 -
0.10279

C 1.10744 -0.00026
1.34796

H 1.72001 -0.88912
1.49744

H 1.72009 0.8885
1.49774

H 0.24363 -0.00033
2.02335

O 1.82397 0.00017 -
0.86024

Methyl Pyrrolidinone
The number of imaginary frequencies: 0
Total Energy (B3LYP/6-31G*)= -
325.94471629
ZPE= -325.804999
Enthalpy= -325.797138

C -1.15663 -0.45327
0.19297

C 1.15639 -0.45362
0.19329

C 0.77798 1.0303 -
0.07053

C -0.77756 1.03066 -
0.07012

H -2.05637 -0.7771 -
0.33946

H -1.33544 -0.61146
1.26475

H 1.19788 1.70083
0.6869

H 1.1653 1.36132 -
1.04085

H -1.19669 1.70085
0.68805

H -1.16528 1.36262 -
1.03996

N -0.00017 -1.27389 -
0.20026

C 0.00003 -1.3903 -
1.66564

H 0.87482 -1.91947 -
1.98127

H -0.87248 -1.92325 -
1.98122

H -0.0021 -0.41291 -
2.10107

O 2.33218 -0.87742 -
0.50154

Methyl Pyrrolidinone
The number of imaginary frequencies: 0
Total Energy (M06/6-311G+(d,p))= -
325.8093828
ZPE= -325.67156
Enthalpy= -325.663666

C -0.50075 -1.34309
0.05295

C 0.13557 0.87519 -
0.11685

C -1.33952 0.80633
0.3062

C -1.73765 -0.52461 -
0.31782

H -0.49936 -2.3329 -
0.35345

H -0.41913 -1.41815
1.11719

H -1.40259 0.75096
1.37291

H -1.93719 1.6317 -
0.02004

H -2.66302 -0.91016
0.05628

H -1.81925 -0.4489 -
1.38201

N 0.57997 -0.4906 -
0.50495

C 1.91786 -0.84833 -0.012

H 2.64179 -0.17871 -
0.42724

H 1.93585 -0.77582
1.05539

H 2.14976 -1.85087 -
0.30534

O 0.84772 1.91254 -
0.13428

Methyl Pyrrolidinone N-oxide
The number of imaginary frequencies: 0
Total Energy (B3LYP/6-31G*)= -
401.06253971
ZPE= -400.91912
Enthalpy= -400.910648

C -1.15663 -0.45327
0.19297

C 1.15639 -0.45362
0.19329

C 0.77798 1.0303 -
0.07053

C -0.77756 1.03066 -
0.07012

H -2.05637 -0.7771 -
0.33946

H -1.33544 -0.61146
1.26475

H 1.19788 1.70083
0.6869

H 1.1653 1.36132 -
1.04085

H -1.19669 1.70085
0.68805

H -1.16528 1.36262 -
1.03996

N -0.00017 -1.27389 -
0.20026

C 0.00003 -1.3903 -
1.66564

H 0.87482 -1.91947 -
1.98127

H -0.87248 -1.92325 -
1.98122

H -0.0021 -0.41291 -
2.10107

O 2.33218 -0.87742 -
0.50154

O -0.00044 -2.50637
0.37472

Methyl Pyrrolidinone N-oxide
The number of imaginary frequencies: 0
Total Energy (M06/6-311G+(d,p))= -
400.9215923
ZPE= -400.780264
Enthalpy= -400.771751

C -0.48471 -1.28217 -0.5346

C -0.0026 0.99053 -
0.06214

C -1.49677 0.84499 -
0.05282

C -1.78687 -0.65491 -
0.0592

H -0.37844 -1.24163 -
1.62724

H -0.30977 -2.29782 -
0.1736

H -1.91474 1.37381
0.80611

H -1.86375 1.35238 -
0.95306

H -2.01179 -1.00562
0.94849

H -2.61807 -0.91988 -
0.7162

N 0.55348 -0.40619
0.06882

C 1.90545 -0.59017 -
0.47633

H 2.54161 0.16753 -
0.02093

H 2.23699 -1.58619 -
0.17945

H 1.89512 -0.48166 -
1.56483

O 0.69431 1.94486 -
0.16503

O 0.52337 -0.49076
1.42871

Pyridine
The number of imaginary frequencies: 0
Total Energy (B3LYP/6-31G*)= -
248.28497285
ZPE= -248.195939
Enthalpy= -248.190725

C -2.54788 -2.09617 0.

C -1.141 -2.09617 0.

C -0.47431 -0.86957 0.

C -1.22838 0.30522 -0.00024

C -2.63158 0.20242 -0.00038

N -3.29111 -0.97253 -0.0002

H 0.62424 -0.82979 -0.00095

H -3.11342 -3.04499 0.00012

H -0.58627 -3.04307 0.00026

H -0.74435 1.29015 -0.00034

H -3.26493 1.10748 -0.00046

Pyridine
The number of imaginary frequencies: 0
Total Energy (B3LYP/6-31G**)= -248.2925931
ZPE= -248.203715
Enthalpy= -248.198499

C -1.14249 -0.72173 -0.00022

C -1.19869 0.67323 -0.00012

C 0.0002 1.38545 0.0001

C 1.19888 0.67291 0.00022

C 1.14229 -0.72204 0.00013

N -0.0002 -1.42093 -0.0001

H 0.00032 2.47233 0.00016

H -2.05988 -1.30864 -0.00037

H -2.15775 1.18284 -0.00023

H 2.1581 1.18221 0.00036

H 2.05951 -1.3092 0.00019

Pyridine
The number of imaginary frequencies: 0
Total Energy (M06/6-311G+(d,p))= -248.15337889
ZPE= -248.06551
Enthalpy= -248.060238

C -1.14254 -0.72168 -0.00022

C -1.19865 0.67327 -0.00012

C 0.00025 1.38544 0.0001

C 1.19889 0.67286 0.00022

C 1.14228 -0.72207 0.00012

N -0.00025 -1.42094 -0.0001

H 0.00041 2.47232 0.00016

H -2.05996 -1.30855 -0.00037

H -2.15768 1.18294 -0.00023

H 2.15812 1.18216 0.00036

H 2.05949 -1.30926 0.00019

Pyridine
The number of imaginary frequencies: 0
Total Energy (B3PW91/6-31G*)= -248.18841608
ZPE= -248.099146
Enthalpy= -248.093923

C -2.54788 -2.09617 0.

C -1.141 -2.09617 0.

C -0.47431 -0.86957 0.

C -1.22838 0.30522 -0.00024

C -2.63158 0.20242 -0.00038

N -3.29111 -0.97253 -0.0002

H 0.62424 -0.82979 -0.00095

H -3.11342 -3.04499 0.00012

H -0.58627 -3.04307 0.00026

H -0.74435 1.29015 -0.00034

H -3.26493 1.10748 -0.00046

Pyridine
The number of imaginary frequencies: 0
Total Energy (B3PW91/6-31G**)= -248.19559822
ZPE= -248.106489
Enthalpy= -248.101263

C -2.54788 -2.09617 0.

C -1.141 -2.09617 0.

C -0.47431 -0.86957 0.

C -1.22838 0.30522 -0.00024

C -2.63158 0.20242 -0.00038

N -3.29111 -0.97253 -0.0002

H 0.62424 -0.82979 -0.00095

H -3.11342 -3.04499 0.00012

H -0.58627 -3.04307 0.00026

H -0.74435 1.29015 -0.00034

H -3.26493 1.10748 -0.00046

Pyridine
The number of imaginary frequencies: 0
Total Energy (B3PW91/6-311G+(d,p))= -248.24903458
ZPE= -248.160401
Enthalpy= -248.155163

C -2.54788 -2.09617 0.

C -1.141 -2.09617 0.

C -0.47431 -0.86957 0.

C -1.22838 0.30522 -0.00024

C -2.63158 0.20242 -0.00038

N -3.29111 -0.97253 -0.0002

H 0.62424 -0.82979 -0.00095

H -3.11342 -3.04499 0.00012

H -0.58627 -3.04307 0.00026

H -0.74435 1.29015 -0.00034

H -3.26493 1.10748 -0.00046

Pyridine
The number of imaginary frequencies: 0
Total Energy (Hartree Fock 6-31G*)= -246.69581967
ZPE= -246.60038
Enthalpy= -246.595443

C -2.09349 -0.73061 0.00597

C -0.68661 -0.73061 0.00597

C -0.01992 0.49599 0.00597

C -0.77399 1.67078 0.00573

C -2.17719 1.56797 0.00559

N -2.83672 0.39302 0.00578

H 1.07862 0.53577 0.00502

H -2.65904 -1.67944 0.00609

H -0.13188 -1.67752 0.00624

H -0.28996 2.6557 0.00563

H -2.81054 2.47304 0.00551

Pyridine N-oxide
The number of imaginary frequencies: 0
Total Energy (B3LYP/6-31G*)= -323.44719083
ZPE= -323.353921
Enthalpy= -323.347923

C -1.14254 -0.72168 -0.00022

C -1.19865 0.67327 -0.00012

C 0.00025 1.38544 0.0001

C 1.19889 0.67286 0.00022

C 1.14228 -0.72207 0.00012

N -0.00025 -1.42094 -0.0001

H 0.00041 2.47232 0.00016

H -2.05996 -1.30855 -0.00037

H -2.15768 1.18294 -0.00023

H 2.15812 1.18216 0.00036

H 2.05949 -1.30926 0.00019

O -0.00048 -2.78094 -0.0002

Pyridine N-oxide
The number of imaginary frequencies: 0
Total Energy (B3LYP/6-31G**)= -323.4550059
ZPE= -323.361915
Enthalpy= -323.355913

C 0.28383 -1.18039 -0.00018

C -1.09931 -1.19458 -0.00009

C -1.82222 0. 0.00012

C -1.09931 1.19458 0.00024

C 0.28383 1.18039 0.00014

N 0.98713 0. -0.00007

H -2.90654 0.
0.0002

H 0.91433 -2.05913 -
0.00034

H -1.60303 -2.15629 -
0.00019

H -1.60303 2.15629
0.00041

H 0.91433 2.05913
0.00022

O 2.26163 0. -
0.00015

Pyridine N-oxide
The number of imaginary frequencies: 0
Total Energy (M06/6-311G+(d,p))= -
323.30740415
ZPE= -323.215418
Enthalpy= -323.209346

C 0.28383 -1.18039 -
0.00018

C -1.0993 -1.19457 -
0.00009

C -1.82223 0. 0.00012

C -1.0993 1.19457
0.00024

C 0.28383 1.18039
0.00014

N 0.98715 0. -0.00007

H -2.90656 0. 0.0002

H 0.91434 -2.05915 -
0.00034

H -1.60306 -2.15628 -
0.00019

H -1.60306 2.15628
0.00041

H 0.91434 2.05915
0.00022

O 2.26164 0. -0.00015

Pyridine N-oxide
The number of imaginary frequencies: 0
Total Energy (B3PW91/6-31G*)= -
323.32403049
ZPE= -323.23042
Enthalpy= -323.224425

C -1.14254 -0.72168 -
0.00022

C -1.19865 0.67327 -
0.00012

C 0.00025 1.38544 0.0001

C 1.19889 0.67286
0.00022

C 1.14228 -0.72207
0.00012

N -0.00025 -1.42094 -
0.0001

H 0.00041 2.47232
0.00016

H -2.05996 -1.30855 -
0.00037

H -2.15768 1.18294 -
0.00023

H 2.15812 1.18216
0.00036

H 2.05949 -1.30926
0.00019

O -0.00048 -2.78094 -
0.0002

Pyridine N-oxide
The number of imaginary frequencies: 0
Total Energy (B3PW91/6-31G**)= -
323.33143154
ZPE= -323.237998
Enthalpy= -323.231997

C -1.14254 -0.72168 -
0.00022

C -1.19865 0.67327 -
0.00012

C 0.00025 1.38544 0.0001

C 1.19889 0.67286
0.00022

C 1.14228 -0.72207
0.00012

N -0.00025 -1.42094 -
0.0001

H 0.00041 2.47232
0.00016

H -2.05996 -1.30855 -
0.00037

H -2.15768 1.18294 -
0.00023

H 2.15812 1.18216
0.00036

H 2.05949 -1.30926
0.00019

O -0.00048 -2.78094 -
0.0002

Pyridine N-oxide
The number of imaginary frequencies: 0
Total Energy (B3PW91/6-311G+(d,p))= -
323.40973925
ZPE= -323.316995
Enthalpy= -323.310954

C -1.14254 -0.72168 -
0.00022

C -1.19865 0.67327 -
0.00012

C 0.00025 1.38544 0.0001

C 1.19889 0.67286
0.00022

C 1.14228 -0.72207
0.00012

N -0.00025 -1.42094 -
0.0001

H 0.00041 2.47232
0.00016

H -2.05996 -1.30855 -
0.00037

H -2.15768 1.18294 -
0.00023

H 2.15812 1.18216
0.00036

H 2.05949 -1.30926
0.00019

O -0.00048 -2.78094 -
0.0002

Pyridine N-oxide
The number of imaginary frequencies: 0
Total Energy (Hartree Fock 6-31G*)= -
321.47376757
ZPE= -321.373766
Enthalpy= -321.36815

C -2.16262 0.83539 -
0.00001

C -0.76123 0.85701 -
0.0001

C -0.09527 2.08267 -
0.0003

C -0.84878 3.25654 -
0.0004

C -2.24802 3.17597 -
0.0003

N -2.86495 1.98161 -
0.00011

H 0.97402 2.12168 -
0.00036

H -2.67825 -0.10217
0.00014

H -0.2072 -0.05839 -
0.00002

H -0.36294 4.20988 -
0.00054

H -2.83061 4.07346 -
0.00038

O -4.22404 1.93202 -0.00002

1-aza-1,5-cyclohexadiene
The number of imaginary frequencies: 0
Total Energy (B3LYP/6-31G*)= -249.45870093
ZPE= -249.347657
Enthalpy= -249.341681

C 0.37908 1.18845 -0.18375

C -0.93979 1.31014 -0.04061

C -1.79539 0.11421 0.2909

C -1.13588 -1.1706 -0.23762

C 0.3445 -1.18968 0.01706

N 1.05209 -0.08239 -0.00258

H -1.92356 0.06011 1.38332

H 1.08605 1.9818 -0.38487

H -1.39333 2.29399 -0.12646

H -1.59346 -2.05336 0.22159

H 0.91659 -2.09979 0.14684

H -2.79905 0.22722 -0.13304

H -1.32822 -1.25515 -1.32117

1-aza-1,5-cyclohexadiene
The number of imaginary frequencies: 0
Total Energy (M06/6-311G+(d,p))= -249.32863153
ZPE= -249.218936
Enthalpy= -249.212941

C -1.30685 0.51792 0.11675

C -0.27511 1.37255 0.04709

C 1.11229 0.84984 -0.23605

C 1.22763 -0.59801 0.26603

C -0.02081 -1.37997 -0.08674

N -1.19517 -0.8713 -0.13781

H 1.31265 0.89812 -1.31938

H -2.32199 0.85394 0.31067

H -0.43782 2.44245 0.15038

H 2.11889 -1.09531 -0.13119

H 0.07163 -2.44909 -0.2945

H 1.87305 1.47698 0.24204

H 1.32686 -0.60202 1.36419

1-aza-1,5-cyclohexadiene N-oxide
The number of imaginary frequencies: 0
Total Energy (B3LYP/6-31G*)= -324.62216936
ZPE= -324.506535
Enthalpy= -324.499747

C -0.19281 -0.39727 0.27347

C 1.1456 -0.49899 0.07719

C 1.99666 0.77312 0.13746

C 1.21655 1.94232 -
0.47833

C -0.18847 2.02146
0.14144

N -0.83095 0.92792
0.43008

H 2.21505 0.98053
1.16419

H -0.78986 -1.28358
0.32721

H 1.60067 -1.4521 -
0.09428

H 1.75709 2.84856 -
0.30104

H -0.64579 2.97332
0.31379

H 2.91098 0.64255 -
0.40279

H 1.10658 1.79106 -
1.53186

O -2.11604 1.00378
0.86871

1-aza-1,5-cyclohexadiene N-oxide
The number of imaginary frequencies: 0
Total Energy (M06/6-311G+(d,p))= -
324.48401976
ZPE= -324.369799
Enthalpy= -324.362988

C 0.37908 1.18845 -
0.18375

C -0.93979 1.31014 -
0.04061

C -1.79539 0.11421
0.2909

C -1.13588 -1.1706 -
0.23762

C 0.3445 -1.18968
0.01706

N 1.05209 -0.08239 -
0.00258

H -1.92356 0.06011
1.38332

H 1.08605 1.9818 -
0.38487

H -1.39333 2.29399 -
0.12646

H -1.59346 -2.05336
0.22159

H 0.91659 -2.09979
0.14684

H -2.79905 0.22722 -
0.13304

H -1.32822 -1.25515 -
1.32117

O 2.3194 -0.01165
0.14449

1-aza-1,3-cyclohexadiene
The number of imaginary frequencies: 0
Total Energy (B3LYP/6-31G*)= -
249.45863067
ZPE= -249.347311
Enthalpy= -249.341338

C 0.12247 1.41622
0.05956

C 1.28988 0.50759 -
0.23369

C 0.98654 -0.92084
0.24342

C -1.28937 -0.54996 -
0.11523

C -1.112 0.89422
0.11551

H 1.72373 -1.62288 -
0.15981

H 1.48855 0.51894 -
1.31824

H 2.20622 0.8724
0.24574

H 0.29014 2.48606
0.16604

H -2.30108 -0.91108 -
0.32081

H -1.99297 1.50892
0.28168

H 1.07631 -0.96859
1.34059

N -0.35371 -1.42388 -
0.09324

1-aza-1,3-cyclohexadiene
The number of imaginary frequencies: 0
Total Energy (M06/6-311G+(d,p))= -
249.32790699
ZPE= -249.217931
Enthalpy= -249.211943

C 0.05418 1.40668
0.05834

C 1.24473 0.55694 -
0.25474

C 1.02053 -0.85133
0.26701

C -1.24639 -0.61173 -
0.12628

C -1.14633 0.82804
0.13011

H 1.8113 -1.52571 -
0.07494

H 1.39612 0.54363 -
1.34672

H 2.16095 0.97686
0.17378

H 0.16962 2.48184
0.1697

H -2.23358 -1.01981 -
0.36247

H -2.05155 1.3979
0.31667

H 1.07007 -0.84637
1.36887

N -0.26904 -1.42569 -
0.09879

1-aza-1,3-cyclohexadiene N-oxide
The number of imaginary frequencies: 0
Total Energy (B3LYP/6-31G*)= -
324.63277973
ZPE= -324.516577
Enthalpy= -324.509843

C 0.6267 1.31775 0.03798

C 1.42079 0.06392 -
0.21697

C 0.65931 -1.17255
0.28757

C -1.42639 0.03063 -
0.04753

C -0.75579 1.24484
0.09511

H 1.13967 -2.09446 -
0.05729

H 1.62495 -0.05519 -
1.29413

H 2.39981 0.10172
0.27882

H 1.14155 2.26987
0.12251

H -2.50727 -0.04326 -
0.0897

H -1.35127 2.14476
0.22445

H 0.67434 -1.17434
1.39069

N -0.71792 -1.15182 -
0.21393

O -1.42449 -2.28268
0.05349

1-aza-1,3-cyclohexadiene N-oxide
The number of imaginary frequencies: 0
Total Energy (M06/6-311G+(d,p))= -324.49391803
ZPE= -324.379107
Enthalpy= -324.372359

C 1.82733 0.14558 0.02194

C 1.14609 -1.17099 -0.25112

C -0.28121 -1.17587 0.29827

C -0.36318 1.22259 -0.05323

C 1.0764 1.26062 0.08901

H -0.89464 -1.97468 -0.11745

H 1.13042 -1.35737 -1.33734

H 1.69393 -2.0095 0.19419

H 2.90984 0.18604 0.09445

H -0.97065 2.10895 -0.1874

H 1.52776 2.2399 0.22214

H -0.28773 -1.26345 1.39158

N -1.03086 0.08546 -0.0064

O -2.29069 -0.02746 -0.10557

1-aza-2,4-cyclohexadiene
The number of imaginary frequencies: 0
Total Energy (B3LYP/6-31G*)= -249.45167887
ZPE= -249.340348
Enthalpy= -249.334173

C -2.30401 -1.31125 0.62248

C -1.05536 -1.80673 -0.13119

C -0.1519 -0.90396 -0.56671

C -0.45591 0.58853 -0.38571

C -1.70425 0.97052 -0.02491

N -2.78378 -0.0306 0.06441

H 0.7636 -1.22337 -1.01919

H -3.08856 -2.03408 0.53947

H -0.89672 -2.85296 -0.28972

H 0.30693 1.32027 -0.55163

H -1.91192 2.00101 0.17478

H -3.51227 0.31062 0.65844

H -2.04402 -1.17826 1.65186

1-aza-2,4-cyclohexadiene
The number of imaginary frequencies: 0
Total Energy (M06/6-311G+(d,p))= -249.32709401
ZPE= -249.216903
Enthalpy= -249.210703
C 1.39067 0.19943 0.18922

C 0.43142 1.33873 -0.08369

C -0.89712 1.12767 -0.05346

C -1.43794 -0.21217 0.11795

C -0.58177 -1.25494 0.00288

N 0.76669 -1.06151 -0.224

H -1.58545 1.95708 -0.20181

H 2.32173 0.32422 -0.37781

H 0.84949 2.32853 -0.24406

H -2.50207 -0.38075 0.23128

H -0.91262 -2.28996 0.00251

H 1.35338 -1.87985 -0.14273

H 1.67716 0.17896 1.26318

1-aza-2,4-cyclohexadiene N-oxide
The number of imaginary frequencies: 0
Total Energy (B3LYP/6-31G*)= -324.57303945
ZPE= -324.457626
Enthalpy= -324.450823

C -0.28085 -1.18491 -0.26375

C 1.22585 -1.16982 0.04782

C 1.87146 0.01417 0.06584

C 1.05357 1.3002 -0.11439

C -0.29787 1.24445 -0.02711

N -0.96005 -0.02079 0.34208

H 2.93331 0.0551 0.19119

H -0.39958 -1.15661 -1.32677

H 1.75565 -2.08257 0.22415

H 1.54544 2.23312 -0.29502

H -0.88229 2.12337 -0.20277

H -0.72605 -2.07669 0.12537

O -2.25038 -0.0092 -0.08746

H -0.93599 -0.10124 1.33855

1-aza-2,4-cyclohexadiene N-oxide
The number of imaginary frequencies: 0
Total Energy (M06/6-311G+(d,p))= -324.44453052
ZPE= 324.329983
Enthalpy= -324.323224

C -0.24836 -1.20084 -0.26519

C 1.22684 -1.15818 0.03638

C 1.84678 0.03148 0.10207

C 1.07425 1.2597 -0.11747

C -0.26053 1.24207 -0.05667

N -0.97969 -0.00998 0.31366

H 2.91511 0.10598 0.28375

H -0.46106 -1.14334 -1.34039

H 1.76833 -2.09344 0.14919

H 1.59192 2.18676 -0.34905

H -0.95202 2.06128 -0.20094

H -0.76377 -2.07453 0.13585

O -2.27625 0.00994 -0.05223

H -0.86456 -0.09778 1.34905

Pyridinium
The number of imaginary frequencies: 0
Total Energy (B3LYP/6-31G*)= -248.65697731
ZPE= -248.553721
Enthalpy= -248.548407

C -0.03244 -0.6621 1.23589

C -0.00269 0.69287 1.26532

C 0.00936 1.47622 -0.05326

C -0.01009 0.79169 -1.21517

C -0.04363 -0.74752 -1.18302

N -0.05324 -1.38613 -0.05082

H 0.03277 2.54596 -0.05137

H -0.04093 -1.21208 2.15368

H 0.01227 1.21173 2.20098

H -0.00229 1.31275 -2.14969

H -0.05917 -1.29655 -2.10129

H -0.07511 -2.38589 -0.05126

Pyridinium
The number of imaginary frequencies: 0
Total Energy (M06/6-311G+(d,p))= -248.51713791
ZPE= -248.415283
Enthalpy= -248.409911

C -0.67805 1.18452 0.

C 0.70573 1.2185 0.

C 1.41588 0.01296 0.

C 0.72825 -1.20555 0.

C -0.65595 -1.19671 0.

N -1.30935 -0.01185 0.

H 2.50153 0.02298 0.

H -1.30651 2.06722 0.

H 1.21529 2.17534 0.

H 1.25524 -2.15289 0.

H -1.26803 -2.09086 0.

H -2.32731 -0.0212 0.

1-hydroxy-Pyridine
The number of imaginary frequencies: 0
Total Energy (B3LYP/6-31G*)= -323.81123177
ZPE= -323.70567
Enthalpy= -323.699038

C -1.16523 -0.8132 0.03587

C -1.21205 0.72488 -0.02244

C -0.05685 1.42088 -0.03123

C 1.26901 0.65134 0.01742

C 1.25249 -0.70323 0.06791

N -0.02711 -1.43997 0.07636

H -0.06515 2.49009 -0.0714

H -2.07813 -1.3713 0.04312

H -2.1514 1.23618 -0.05563

H 2.19958 1.17946 0.01156

H 2.17539 -1.2436 0.10195

O -0.01475 -2.79896 0.12744

H 0.77863 -3.1281 -0.30128

1-hydroxy-Pyridine
The number of imaginary frequencies: 0
Total Energy (M06/6-311G+(d,p))= -323.66548298
ZPE= -323.560957
Enthalpy= -323.554271

C 0.23341 1.19246 -0.00621

C -1.15148 1.2099 0.00375

C -1.85266 0.00002 0.01094

C -1.15151 -1.2099 0.00332

C 0.23337 -1.1925 -0.00605

N 0.8679 0.00001 0.00682

H -2.93802 0.00004 0.0132

H 0.87049 2.06819 -0.03361

H -1.6652 2.16469 -0.00291

H -1.66533 -2.16463 -0.00298

H 0.87062 -2.06813 -0.03229

O 2.24805 -0.0001 -0.10418

H 2.60097 0.00066 0.80976

3-cyano-Pyridine
The number of imaginary frequencies: 0
Total Energy (B3LYP/6-31G*)= -340.52598067
ZPE= -340.438405
Enthalpy= -340.431459

C -3.05562 -2.66599 -0.00376

C -1.65433 -2.64359 0.01081

C -0.98909 -1.41677 0.011

C -1.74292 -0.24238 -0.00339

C -3.14204 -0.32347 -0.0176

N -3.75522 -1.51899 -0.01742

H 0.08013 -1.37726 0.02198

H -3.57194 -3.60317 -0.00399

H -1.25726 0.71105 -0.00357

H -3.72602 0.57305 -0.02868

C -0.85613 -3.9605 0.02645

N -0.26184 -4.94099 0.03809

3-cyano-Pyridine
The number of imaginary frequencies: 0
Total Energy (M06/6-311G+(d,p))= -340.3605528
ZPE= -340.273929
Enthalpy= -340.266949

C	0.12048	-1.18974	0.
C	-0.59443	0.0218	0.
C	0.12883	1.2242	0.
C	1.5174	1.15859	0.
C	2.1282	-0.0978	0.
N	1.45178	-1.25419	0.
H	-0.39357	2.1756	0.
H	-0.41672	-2.13554	0.
H	2.11898	2.06197	0.
H	3.21325	-0.18147	0.
C	-2.02657	0.01677	0.
N	-3.18969	0.00799	0.

3-cyano-Pyridine N-oxide
The number of imaginary frequencies: 0
Total Energy (B3LYP/6-31G*)= -415.68426162
ZPE= -415.592623
Enthalpy= -415.584834

C	0.06948	-0.91129	0.
C	-0.91135	0.07723	0.
C	-0.55674	1.43507	0.
C	0.80257	1.74355	0.
C	1.75609	0.74304	0.
N	1.40225	-0.58791	0.
H	-1.31838	2.2051	0.
H	-0.13164	-1.97347	0.
H	1.13791	2.77546	0.
H	2.82567	0.90402	0.
O	2.28982	-1.49665	0.
C	-2.28998	-0.32043	0.
N	-3.40976	-0.63222	0.

3-cyano-Pyridine N-oxide
The number of imaginary frequencies: 0
Total Energy (M06/6-311G+(d,p))= -415.51038779
ZPE= -415.419669
Enthalpy= -415.411843

C	0.	0.91394	0.
C	0.90284	-0.14628	0.
C	0.44603	-1.47324	0.
C	-0.93279	-1.6775	0.
C	-1.8075	-0.6074	0.
N	-1.3535	0.69281	0.
H	1.14694	-2.29894	0.
H	0.28128	1.95775	0.
H	-1.3456	-2.68092	0.
H	-2.88622	-0.6866	0.
O	-2.16942	1.66639	0.
C	2.30771	0.14542	0.
N	3.44795	0.37118	0.

4-cyano-Pyridine
The number of imaginary frequencies: 0
Total Energy (B3LYP/6-31G*)= -340.52459398
ZPE= -340.437063
Enthalpy= -340.430124

C	-0.40029	-0.58541	-0.01707
C	1.00108	-0.56384	-0.01753
C	1.66703	0.66249	-0.00048

C 0.9138 1.83722
0.01661

C -0.48544 1.75697
0.01624

N -1.0993 0.56192 -0.0004

H -0.91715 -1.5222 -
0.03009

H 1.55521 -1.47908 -
0.03083

H 1.4 2.79028 0.02988

H -1.06896 2.65376
0.02929

C 3.20602 0.71843 -
0.00052

N 4.35186 0.76015 -
0.00055

4-cyano-Pyridine
The number of imaginary frequencies: 0
Total Energy (M06/6-311G+(d,p))= -
340.35906587
ZPE= -340.272444
Enthalpy= -340.265476

C -1.50627 1.14355 0.

C -0.11359 1.20613 0.

C 0.60141 0. 0.

C -0.11359 -1.20613 0.

C -1.50627 -1.14355 0.

N -2.20212 0. 0.

H -2.0922 2.06068 0.

H 0.40392 2.15925 0.

H 0.40392 -2.15925 0.

H -2.0922 -2.06068 0.

C 2.03706 0. 0.

N 3.19985 0. 0.

4-cyano-Pyridine N-oxide
The number of imaginary frequencies: 0
Total Energy (B3LYP/6-31G*)= -
415.68843336
ZPE= -415.596614
Enthalpy= -415.588847

C -0.40024 -0.58449 -
0.00878

C 1.00116 -0.56304 -
0.00932

C 1.66727 0.66251 -
0.0006

C 0.91391 1.83643
0.00824

C -0.48534 1.75604
0.00828

N -1.1024 0.56179 -
0.00007

H -0.91597 -1.52195 -
0.01535

H 1.55505 -1.47847 -
0.01634

H 1.39987 2.7897
0.01492

H -1.0678 2.65359 0.015

O -2.4615 0.51237
0.00034

C 3.20624 0.71849 -
0.00069

N 4.3521 0.76008 -
0.00076

4-cyano-Pyridine N-oxide
The number of imaginary frequencies: 0
Total Energy (M06/6-311G+(d,p))= -
415.51409429
ZPE= -415.423306
Enthalpy= -415.415492

C 1.04962 -1.18413
0.00001

C -0.32757 -1.20253
0.00001

C -1.0566 0.00001 0.

C -0.32757 1.20254
0.00001

C 1.04963 1.18413
0.00001

N 1.75342 0. 0.

H 1.67902 -2.06362 0.

H -0.83939 -2.1586
0.00001

H -0.83938 2.15861
0.00001

H 1.67903 2.06361
0.00001

O 3.02055 -0.00001 -
0.00002

C -2.48419 0.00001 0.

N -3.64821 -0.00001 -
0.00001

2-hydroxy-Pyridine
The number of imaginary frequencies: 0
Total Energy (B3LYP/6-31G*)= -
323.51588142
ZPE= -323.422209
Enthalpy= -323.41607

C -1.70067 -0.65433 -
0.00164

C -0.29927 -0.63503 -
0.00012

C 0.36866 0.59034
0.00169

C -0.38269 1.76641
0.00194

C -1.78206 1.68842
0.00038

N -2.39784 0.49424 -
0.00135

H 1.43801 0.62749
0.00287

H -2.21904 -1.59038 -
0.00303

H 0.25339 -1.55125 -
0.00035

H 0.10505 2.71878
0.00331

O -2.55997 2.88832
0.00062

H -2.73308 3.15764 -
0.90442

2-hydroxy-Pyridine
The number of imaginary frequencies: 0
Total Energy (M06/6-311G+(d,p))= -
323.38753442
ZPE= -323.294564
Enthalpy= -323.288361

C 0.99985 -1.22953 -
0.00002

C 1.81732 -0.10489
0.00009

C 1.20135 1.15327 -
0.00008

C -0.18351 1.23798 -
0.00002

C -0.90375 0.03356 -
0.00003

N -0.34147 -1.17223 -
0.00006

H 1.80054 2.05994 -
0.00002

H 1.42872 -2.22994 -
0.00004

H 2.89752 -0.20675
0.00025

H -0.71092 2.18538
0.00005

O -2.25708 0.08689
0.00006

H -2.55648 -0.84043
0.00002

2-carboxy-Pyridine
The number of imaginary frequencies: 0
Total Energy (B3LYP/6-31G*)= -
436.85938679
ZPE= -436.755087
Enthalpy= -436.747324

C 1.7186 -1.23797
0.00032

C 2.53278 -0.10114
0.00033

C 1.92424 1.15289
0.00015

C 0.53308 1.21892 -
0.00005

C -0.18796 0.01996 -
0.00008

N 0.38442 -1.19228
0.00013

H 2.52032 2.06116
0.00016

H 2.16171 -2.23235
0.00045

H 3.61395 -0.20322
0.00048

H -0.00286 2.16117 -
0.00018

C -1.68567 0.10587 -
0.00023

O -2.29595 1.15768 -
0.00036

O -2.29682 -1.09449 -
0.00017

H -1.63544 -1.79032 -
0.00013

2-carboxy-Pyridine
The number of imaginary frequencies: 0
Total Energy (M06/6-311G+(d,p))= -
436.70529032
ZPE= -436.601643
Enthalpy= -436.593822

C 1.66966 -1.23606
0.00031

C 2.5029 -0.11529
0.00033

C 1.92164 1.1535
0.00015

C 0.53216 1.25801 -
0.00006

C -0.21239 0.07885 -
0.00007

N 0.33633 -1.14606
0.00012

H 2.54217 2.04515
0.00016

H 2.08563 -2.24104
0.00045

H 3.5813 -0.23965
0.00049

H 0.0157 2.2112 -
0.00021

C -1.7219 0.1194 -
0.00029

O -2.3546 1.15072 -
0.00031

O -2.29271 -1.09554 -
0.00018

H -1.55305 -1.74509 -
0.0001

2-carboxy-Pyridine N-oxide
The number of imaginary frequencies: 0
Total Energy (B3LYP/6-31G*)= -
512.02448163
ZPE= -511.916422
Enthalpy= -511.907975

C 1.66966 -1.23606
0.00031

C 2.5029 -0.11529
0.00033

C 1.92164 1.1535
0.00015

C 0.53216 1.25801 -
0.00006

C -0.21239 0.07885 -
0.00007

N 0.33633 -1.14606
0.00012

H 2.54217 2.04515
0.00016

H 2.08563 -2.24104
0.00045

H 3.5813 -0.23965
0.00049

H 0.0157 2.2112 -
0.00021

C -1.7219 0.1194 -
0.00029

O -2.3546 1.15072 -
0.00031

O -2.29271 -1.09554 -
0.00018

H -1.55305 -1.74509 -
0.0001

O -0.44112 -2.26194
0.0001

2-carboxy-Pyridine N-oxide
The number of imaginary frequencies: 0
Total Energy (M06/6-311G+(d,p))= -
511.8594662
ZPE= -511.752215
Enthalpy= -511.743647

C -1.77734 1.02321 0.0003

C -2.55957 -0.11467
0.0003

C -1.9576 -1.37621
0.00013

C -0.57036 -1.43871 -
0.00004

C 0.2062 -0.28216 -
0.00005

N -0.41282 0.94794
0.00012

H -2.55597 -2.28072
0.00014

H -2.15273 2.03761
0.00042

H -3.63859 -0.00061
0.00045

H -0.02807 -2.37719 -
0.00017

C 1.71727 -0.41024 -
0.0002

O 2.21834 -1.515 -
0.00038

O 2.41535 0.71685 -
0.00026

H 1.76035 1.48162 -
0.00011

O 0.26045 2.0602
0.00011

2-Pyridone
The number of imaginary frequencies: 0
Total Energy (B3LYP/6-31G*)= -
323.51790653
ZPE= -323.424021
Enthalpy= -323.417805

C -1.8924 0.68529
0.13687

C -0.46108 0.93021 -
0.35424

C -0.02365 2.19097 -
0.49075

C -0.92536 3.34888 -
0.05024

C -2.11117 3.11354
0.57158

N -2.74047 1.77228
0.67189

H 0.94411 2.38593 -
0.90345

H 0.17358 0.10651 -
0.60648

H -0.60593 4.35708 -
0.21275

H -2.60985 3.94024
1.03287

O -2.37024 -0.47619
0.05803

H -3.58952 1.74449
0.1443

2-Pyridone
The number of imaginary frequencies: 0
Total Energy (M06/6-311G+(d,p))= -
323.38844194
ZPE= -323.295431
Enthalpy= -323.289162

C -1.06793 0.06468
0.00025

C -0.24816 1.26434 -
0.00034

C 1.11647 1.20264
0.00011

C 1.80662 -0.04718
0.00008

C 1.06195 -1.18969 -
0.00016

N -0.30094 -1.12399
0.00007

H 1.6948 2.12371
0.00041

H -0.78218 2.20802 -
0.0001

H 2.8886 -0.09729
0.00004

H 1.49261 -2.18554 -
0.00043

O -2.29317 0.00283 -
0.00003

H -0.85557 -1.97241
0.00023

Piperidine
The number of imaginary frequencies: 0
Total Energy (B3LYP/6-31G*)= -
251.90382822
ZPE= -251.744054
Enthalpy= -251.737514

C 0.80382 -0.90026
0.03176

C 1.37062 0.53089 -
0.01985

C 0.84842 1.31759
1.18814

C -0.68376 1.33307
1.13349

C -1.20021 -0.11774
1.15683

H 2.43973 0.49715
0.00788

H 1.12156 -1.37685
0.93553

H 1.16457 -1.4533 -
0.81021

H 1.17087 0.84406
2.09185

H 1.22361 2.31925
1.15976

H -1.0683 1.86694 1.9773

H -1.00586 1.81248
0.23275

H -0.87764 -0.59622
2.05788

H -2.26948 -0.1124
1.11767

H 1.05345 1.00837 -
0.92335

N -0.67191 -0.8579 -
0.00685

H -0.96641 -0.40343 -
0.84752

Piperidine
The number of imaginary frequencies: 0
Total Energy (M06/6-311G+(d,p))= -
251.7736967
ZPE= -251.616109
Enthalpy= -251.609536

C 0.00832 -0.79156
1.21662

C 0.00832 0.74696 1.2659

C -0.63986 1.33068 0.

C 0.00832 0.74696 -
1.2659

C 0.00832 -0.79156 -
1.21662

H -0.51435 1.09782
2.1661

H -1.02808 -1.15594
1.27119

H 0.53487 -1.20911
2.08319

H -1.71252 1.08526 0.

H -0.57077 2.4258 0.

H -0.51435 1.09782 -
2.1661

H 1.04647 1.10377 -
1.34443

H -1.02808 -1.15594 -
1.27119

H 0.53487 -1.20911 -
2.08319

H 1.04647 1.10377
1.34443

N 0.60573 -1.35515 0.

H 1.60481 -1.14691 0.

Piperidine
The number of imaginary frequencies: 0
Total Energy (B3PW91/6-31G*)= -
251.8164414
ZPE= -251.656285
Enthalpy= -251.649749

C 0.80382 -0.90026
0.03176

C 1.37062 0.53089 -
0.01985

C 0.84842 1.31759
1.18814

C -0.68376 1.33307
1.13349

C -1.20021 -0.11774
1.15683

H 2.43973 0.49715
0.00788

H 1.12156 -1.37685
0.93553

H 1.16457 -1.4533 -
0.81021

H 1.17087 0.84406
2.09185

H 1.22361 2.31925
1.15976

H -1.0683 1.86694 1.9773

H -1.00586 1.81248
0.23275

H -0.87764 -0.59622
2.05788

H -2.26948 -0.1124
1.11767

H 1.05345 1.00837 -
0.92335

N -0.67191 -0.8579 -
0.00685

H -0.96641 -0.40343 -
0.84752

Piperidine
The number of imaginary frequencies: 0
Total Energy (B3PW91/6-31G**)= -
251.832313
ZPE= -251.672731
Enthalpy= -251.666174

C 0.80382 -0.90026
0.03176

C 1.37062 0.53089 -
0.01985

C 0.84842 1.31759
1.18814

C -0.68376 1.33307
1.13349

C -1.20021 -0.11774
1.15683

H 2.43973 0.49715
0.00788

H 1.12156 -1.37685
0.93553

H 1.16457 -1.4533 -
0.81021

H 1.17087 0.84406
2.09185

H 1.22361 2.31925
1.15976

H -1.0683 1.86694 1.9773

H -1.00586 1.81248
0.23275

H -0.87764 -0.59622
2.05788

H -2.26948 -0.1124
1.11767

H 1.05345 1.00837 -
0.92335

N -0.67191 -0.8579 -
0.00685

H -0.96641 -0.40343 -
0.84752

Piperidine
The number of imaginary frequencies: 0
Total Energy (B3PW91/6-311G+(d,p))= -
251.88472135
ZPE= -251.726148
Enthalpy= -251.719542

C 0.80382 -0.90026
0.03176

C 1.37062 0.53089 -
0.01985

C 0.84842 1.31759
1.18814

C -0.68376 1.33307
1.13349

C -1.20021 -0.11774
1.15683

H 2.43973 0.49715
0.00788

H 1.12156 -1.37685
0.93553

H 1.16457 -1.4533 -
0.81021

H 1.17087 0.84406
2.09185

H 1.22361 2.31925
1.15976

H -1.0683 1.86694 1.9773

H -1.00586 1.81248
0.23275

H -0.87764 -0.59622
2.05788

H -2.26948 -0.1124 1.11767

H 1.05345 1.00837 -0.92335

N -0.67191 -0.8579 -0.00685

H -0.96641 -0.40343 -0.84752

Piperidine N-oxide
The number of imaginary frequencies: 0
Total Energy (B3LYP/6-31G*)= -327.0440554
ZPE= -326.879636
Enthalpy= -326.872325

C 0.80347 -0.89926 0.03148

C 1.37057 0.53182 -0.01909

C 0.84895 1.31772 1.18899

C -0.68267 1.33355 1.13361

C -1.19976 -0.11706 1.15609

H 2.43967 0.49773 0.00868

H 1.12103 -1.37637 0.93504

H 1.16483 -1.45127 -0.8109

H 1.17105 0.84358 2.0925

H 1.2245 2.31927 1.16133

H -1.06737 1.86712 1.97753

H -1.00423 1.81349 0.23297

H -0.87757 -0.59598 2.05705

H -2.26903 -0.11045 1.11688

H 1.05356 1.00998 -0.92229

N -0.67284 -0.85879 -0.00789

H -0.96914 -0.40744 -0.84961

O -1.15312 -2.13083 0.02136

Piperidine N-oxide
The number of imaginary frequencies: 0
Total Energy (M06/6-311G+(d,p))= -326.91190613
ZPE= -326.749232
Enthalpy= -326.741974

C 0.09165 0.39957 1.23954

C 0.09165 -1.13015 1.26347

C 0.74857 -1.7078 0.

C 0.09165 -1.13015 -1.26347

C 0.09165 0.39957 -1.23954

H 0.61246 -1.47217 2.16602

H 1.10029 0.82154 1.22377

H -0.44991 0.84998 2.07398

H 1.81803 -1.45531 0.

H 0.68088 -2.80185 0.

H 0.61246 -1.47217 -2.16602

H -0.9413 -1.49953 -
1.34587

H 1.10029 0.82154 -
1.22377

H -0.44991 0.84998 -
2.07398

H -0.9413 -1.49953
1.34587

N -0.56273 0.96836 0.

H -1.53432 0.59157 0.

O -0.54495 2.31266 0.

Piperidine N-oxide
The number of imaginary frequencies: 0
Total Energy (B3PW91/6-31G*)= -
326.92875651
ZPE= -326.763861
Enthalpy= -326.756572

C 0.09302 0.39957
1.23188

C 0.09302 -1.12482 1.258

C 0.74921 -1.70012 0.

C 0.09302 -1.12482 -1.258

C 0.09302 0.39957 -
1.23188

H 0.61267 -1.46598
2.16164

H 1.10203 0.82206
1.21291

H -0.44526 0.85182
2.06809

H 1.81835 -1.44562 0.

H 0.68408 -2.79446 0.

H 0.61267 -1.46598 -
2.16164

H -0.93988 -1.49501 -
1.34041

H 1.10203 0.82206 -
1.21291

H -0.44526 0.85182 -
2.06809

H -0.93988 -1.49501
1.34041

N -0.56203 0.96462 0.

H -1.52969 0.57835 0.

O -0.55316 2.29841 0.

Piperidine N-oxide
The number of imaginary frequencies: 0
Total Energy (B3PW91/6-31G**)= -
326.94396243
ZPE= -326.779742
Enthalpy= -326.772426

C 0.80347 -0.89926
0.03148

C 1.37057 0.53182 -
0.01909

C 0.84895 1.31772
1.18899

C -0.68267 1.33355
1.13361

C -1.19976 -0.11706
1.15609

H 2.43967 0.49773
0.00868

H 1.12103 -1.37637
0.93504

H 1.16483 -1.45127 -
0.8109

H 1.17105 0.84358
2.0925

H 1.2245 2.31927
1.16133

H -1.06737 1.86712
1.97753

H -1.00423 1.81349
0.23297

H -0.87757 -0.59598
2.05705

H -2.26903 -0.11045
1.11688

H 1.05356 1.00998 -
0.92229

N -0.67284 -0.85879 -
0.00789

H -0.96914 -0.40744 -
0.84961

O -1.15312 -2.13083
0.02136

Piperidine N-oxide
The number of imaginary frequencies: 0
Total Energy (B3PW91/6-311G+(d,p))= -
327.02772616
ZPE= -326.863969
Enthalpy= -326.856636

C 0.80347 -0.89926
0.03148

C 1.37057 0.53182 -
0.01909

C 0.84895 1.31772
1.18899

C -0.68267 1.33355
1.13361

C -1.19976 -0.11706
1.15609

H 2.43967 0.49773
0.00868

H 1.12103 -1.37637
0.93504

H 1.16483 -1.45127 -
0.8109

H 1.17105 0.84358
2.0925

H 1.2245 2.31927
1.16133

H -1.06737 1.86712
1.97753

H -1.00423 1.81349
0.23297

H -0.87757 -0.59598
2.05705

H -2.26903 -0.11045
1.11688

H 1.05356 1.00998 -
0.92229

N -0.67284 -0.85879 -
0.00789

H -0.96914 -0.40744 -
0.84961

O -1.15312 -2.13083
0.02136

Piperidinium
The number of imaginary frequencies: 0
Total Energy (B3LYP/6-31G*)= -
252.2859402
ZPE= -252.110728
Enthalpy= -252.104086

C 0.2563 0.70643 1.21342

C -0.25474 -0.74618
1.24371

C 0.25761 -1.48092 0.

C -0.25474 -0.74618 -
1.24371

C 0.2563 0.70643 -
1.21342

H 0.10397 -1.23684
2.12432

H 1.32629 0.70745
1.21061

H -0.09987 1.22324
2.07999

H 1.3276 -1.48454 0.

H -0.09992 -2.48943 0.

H 0.10397 -1.23684 -2.12432

H -1.32474 -0.74843 -1.24647

H 1.32629 0.70745 -1.21061

H -0.09987 1.22324 -2.07999

H -1.32474 -0.74843 1.24647

N -0.23698 1.38978 0.

H -1.23696 1.3833 0.

H 0.09254 2.33393 0.

Piperidinium
The number of imaginary frequencies: 0
Total Energy (M06/6-311G+(d,p))= -252.14582512
ZPE= -251.973176
Enthalpy= -251.966535

C -0.01538 -0.74124 1.27281

C -0.01538 0.78498 1.26832

C -0.67423 1.35037 0.

C -0.01538 0.78498 -1.26832

C -0.01538 -0.74124 -1.27281

H -0.54349 1.11969 2.16719

H -1.02853 -1.15319 1.26942

H 0.5376 -1.16721 2.11354

H -1.74484 1.10712 0.

H -0.60063 2.44169 0.

H -0.54349 1.11969 -2.16719

H 1.0163 1.15152 -1.36189

H -1.02853 -1.15319 -1.26942

H 0.5376 -1.16721 -2.11354

H 1.0163 1.15152 1.36189

N 0.6456 -1.25965 0.

H 1.63257 -0.97419 0.

H 0.64443 -2.28581 0.

Piperideine
The number of imaginary frequencies: 0
Total Energy (B3LYP/6-31G*)= -250.68792691
ZPE= -250.552631
Enthalpy= -250.546306

C -2.07985 -1.79356 -0.0563

C -0.55751 -1.63904 -0.2454

C -0.48945 0.3334 1.20161

C -2.02668 0.37596 1.1092

C -2.53773 -1.05764 1.20991

H -0.01514 -2.40174 -0.76407

H -2.56803 -1.36076 -0.90439

H -2.33982 -2.82885 0.01785

H -0.21326 -0.01662 2.17429

H -0.10058 1.3184 1.04845

H -2.40771 0.97091 1.91279

H -2.34223 0.79797 0.17795

H -2.13427 -1.51611 2.0885

H -3.60626 -1.07955 1.26157

N 0.08263 -0.59333 0.20215

Piperideine
The number of imaginary frequencies: 0
Total Energy (M06/6-311G+(d,p))= -250.55535317
ZPE= -250.421846
Enthalpy= -250.415526

C -1.46593 -0.03236 -0.10309

C -0.6106 -1.28267 -0.05486

C 1.44143 -0.12494 0.12621

C 0.72363 1.15552 -0.32129

C -0.67552 1.21859 0.30171

H -1.15183 -2.23392 -0.11839

H -1.86442 0.07197 -1.1245

H -2.3435 -0.17948 0.54075

H 1.78447 -0.02729 1.1669

H 2.34748 -0.28683 -0.47088

H 1.32103 2.03397 -0.04786

H 0.63495 1.15955 -1.41679

H -0.58395 1.25474 1.39585

H -1.20776 2.12749 -0.00174

N 0.65507 -1.36073 0.05494

Piperideine N-oxide
The number of imaginary frequencies: 0
Total Energy (B3LYP/6-31G*)= -325.85702784
ZPE= -325.716731
Enthalpy= -325.709625

C -2.08123 -1.79408 -0.05013

C -0.56199 -1.62795 -0.25884

C -0.49373 0.33486 1.19748

C -2.02953 0.37622 1.10149

C -2.53384 -1.05886 1.21551

H -0.02924 -2.37362 -0.81115

H -2.58157 -1.36391 -0.89245

H -2.33254 -2.83136 0.02599

H -0.22584 -0.01254 2.17342

H -0.10293 1.31897 1.04352

H -2.41225 0.97676 1.9001

H -2.3462 0.7904 0.1671

H -2.12261 -1.51065
2.09395

H -3.60186 -1.08654
1.27436

N 0.0904 -0.5958
0.20607

O 1.36173 -0.3776 -
0.22488

Piperideine N-oxide
The number of imaginary frequencies: 0
Total Energy (M06/6-311G+(d,p))= -
325.71737713
ZPE= -325.578818
Enthalpy= -325.571733

C 1.1169 1.27399 -
0.10727

C -0.38305 1.21071 -
0.07714

C -0.41689 -1.23105
0.09571

C 1.05279 -1.21775 -
0.31594

C 1.77604 -0.03767
0.3384

H -0.98671 2.10985 -
0.13628

H 1.45321 1.52909 -
1.12602

H 1.44829 2.10752
0.52648

H -0.5494 -1.55471
1.13348

H -1.02854 -1.87903 -
0.53451

H 1.50858 -2.17376 -
0.03244

H 1.13117 -1.13916 -
1.40856

H 1.71378 -0.1312
1.43079

H 2.83955 -0.0345
0.07564

N -1.08114 0.10558
0.00697

O -2.35458 0.05469
0.0525

Benzene
The number of imaginary frequencies: 0
Total Energy (B3LYP/6-31G*)= -
232.24865193
ZPE= -232.147884
Enthalpy= -232.142551

C -0.07905 0.01976 0.

C 1.31611 0.01976 0.

C 2.01365 1.22751 0.

C 1.31599 2.43602 -
0.0012

C -0.07883 2.43594 -
0.00168

C -0.77643 1.22774 -
0.00068

H -0.62881 -0.93255
0.00045

H 1.86562 -0.93275
0.00132

H 3.11333 1.22759
0.00063

H 1.86619 3.38817 -
0.00126

H -0.62895 3.38823 -
0.00263

H -1.87604 1.22792 -
0.00086

Benzene
The number of imaginary frequencies: 0
Total Energy (M06/6-311G+(d,p))= -
232.11261199

ZPE= -232.013098
Enthalpy= -232.007708

C 1.06282 -0.9059 -0.00001

C -0.25327 -1.37325 -0.00007

C -1.31599 -0.46745 0.00006

C -1.06275 0.90599 -0.00001

C 0.25315 1.37327 -0.00006

C 1.31603 0.46735 0.00006

H 1.8897 -1.61101 0.00007

H -0.45015 -2.44198 -0.00008

H -2.34004 -0.83108 0.00015

H -1.8898 1.61089 -0.00001

H 0.45028 2.44195 -0.00002

H 2.33999 0.83123 0.00006

Benzene
The number of imaginary frequencies: 0
Total Energy (B3PW91/6-31G*)= -232.15906676
ZPE= -232.058021
Enthalpy= -232.05268

C -0.07905 0.01976 0.

C 1.31611 0.01976 0.

C 2.01365 1.22751 0.

C 1.31599 2.43602 -0.0012

C -0.07883 2.43594 -0.00168

C -0.77643 1.22774 -0.00068

H -0.62881 -0.93255 0.00045

H 1.86562 -0.93275 0.00132

H 3.11333 1.22759 0.00063

H 1.86619 3.38817 -0.00126

H -0.62895 3.38823 -0.00263

H -1.87604 1.22792 -0.00086

Benzene
The number of imaginary frequencies: 0
Total Energy (B3PW91/6-31G**)= -232.16807009
ZPE= -232.067176
Enthalpy= -232.061831

C -0.07905 0.01976 0.

C 1.31611 0.01976 0.

C 2.01365 1.22751 0.

C 1.31599 2.43602 -0.0012

C -0.07883 2.43594 -0.00168

C -0.77643 1.22774 -0.00068

H -0.62881 -0.93255 0.00045

H 1.86562 -0.93275 0.00132

H 3.11333 1.22759 0.00063

H 1.86619 3.38817 -0.00126

H -0.62895 3.38823 -0.00263

H -1.87604 1.22792 -0.00086

Benzene
The number of imaginary frequencies: 0
Total Energy (B3PW91/6-311G+(d,p))= -232.21577033
ZPE= -232.115389
Enthalpy= -232.110038

C -0.07905 0.01976 0.

C 1.31611 0.01976 0.

C 2.01365 1.22751 0.

C 1.31599 2.43602 -0.0012

C -0.07883 2.43594 -0.00168

C -0.77643 1.22774 -0.00068

H -0.62881 -0.93255 0.00045

H 1.86562 -0.93275 0.00132

H 3.11333 1.22759 0.00063

H 1.86619 3.38817 -0.00126

H -0.62895 3.38823 -0.00263

H -1.87604 1.22792 -0.00086

Phenol
The number of imaginary frequencies: 1 (-350.23 cm^{-1})
Total Energy (B3LYP/6-31G*)= -307.45835198
ZPE= -307.354566
Enthalpy= -307.34847

C 0.49055 -0.07725 0.00017

C 1.89195 -0.07726 0.00056

C 2.59266 1.13639 -0.00004

C 1.89197 2.35004 -0.00102

C 0.49057 2.35005 -0.00141

C -0.21014 1.1364 -0.00082

H -0.04445 -1.00389 0.00062

H 2.42695 -1.00391 0.00131

H 3.66266 1.13638 0.00026

H -0.04443 3.2767 -0.00217

H -1.28014 1.13641 -0.00111

O 2.60697 3.58845 -0.00163

H 2.76723 3.86499 -0.90686

Phenol
The number of imaginary frequencies: 1 (-358.89 cm^{-1})
Total Energy (M06/6-311G+(d,p))= -307.32521462
ZPE= -307.222264
Enthalpy= -307.216106

C 0.49055 -0.07725 0.00017

C 1.89195 -0.07726 0.00056

C 2.59266 1.13639 -0.00004

C 1.89197 2.35004 -0.00102

C 0.49057 2.35005 -0.00141

C -0.21014 1.1364 -0.00082

H -0.04445 -1.00389 0.00062

H 2.42695 -1.00391 0.00131

H 3.66266 1.13638 0.00026

H -0.04443 3.2767 -0.00217

H -1.28014 1.13641 -0.00111

O 2.60697 3.58845 -0.00163

H 2.76723 3.86499 -0.90686

Phenol
The number of imaginary frequencies: 1 (-351.81 cm^{-1})
Total Energy (B3PW91/6-31G*)= -307.34118811
ZPE= -307.237045
Enthalpy= -307.230945

C 0.49055 -0.07725 0.00017

C 1.89195 -0.07726 0.00056

C 2.59266 1.13639 -0.00004

C 1.89197 2.35004 -0.00102

C 0.49057 2.35005 -0.00141

C -0.21014 1.1364 -0.00082

H -0.04445 -1.00389 0.00062

H 2.42695 -1.00391 0.00131

H 3.66266 1.13638 0.00026

H -0.04443 3.2767 -0.00217

H -1.28014 1.13641 -0.00111

O 2.60697 3.58845 -0.00163

H 2.76723 3.86499 -0.90686

Phenol
The number of imaginary frequencies: 1 (-352.75 cm^{-1})
Total Energy (B3PW91/6-31G**)= -307.35457193
ZPE= -307.250439
Enthalpy= -307.244334

C 0.49055 -0.07725 0.00017

C 1.89195 -0.07726 0.00056

C 2.59266 1.13639 -0.00004

C 1.89197 2.35004 -0.00102

C 0.49057 2.35005 -0.00141

C -0.21014 1.1364 -0.00082

H -0.04445 -1.00389 0.00062

H 2.42695 -1.00391 0.00131

H 3.66266 1.13638 0.00026

H -0.04443 3.2767 -
0.00217

H -1.28014 1.13641 -
0.00111

O 2.60697 3.58845 -
0.00163

H 2.76723 3.86499 -
0.90686

Phenol
The number of imaginary frequencies: 1 (-355.52 cm^{-1})
Total Energy (B3PW91/6-311G+(d,p))= -307.42853754
ZPE= -307.32493
Enthalpy= -307.318804

C 0.49055 -0.07725
0.00017

C 1.89195 -0.07726
0.00056

C 2.59266 1.13639 -
0.00004

C 1.89197 2.35004 -
0.00102

C 0.49057 2.35005 -
0.00141

C -0.21014 1.1364 -
0.00082

H -0.04445 -1.00389
0.00062

H 2.42695 -1.00391
0.00131

H 3.66266 1.13638
0.00026

H -0.04443 3.2767 -
0.00217

H -1.28014 1.13641 -
0.00111

O 2.60697 3.58845 -
0.00163

H 2.76723 3.86499 -
0.90686

Phenoxide
The number of imaginary frequencies: 0
Total Energy (B3LYP/6-31G*)= -306.88416774
ZPE= -306.793442
Enthalpy= -306.787254

C 1.04882 -0.56995
0.00005

C 2.45022 -0.57002
0.00054

C 3.15099 0.64359 0.

C 2.45035 1.85727 -
0.00103

C 1.04895 1.85735 -
0.00151

C 0.34819 0.64374 -
0.00097

H 0.51377 -1.49657
0.00046

H 2.98517 -1.4967
0.00131

H 4.22099 0.64353
0.00036

H 2.9854 2.78389 -
0.00144

H 0.514 2.78402 -
0.00229

O -1.08181 0.64381 -
0.00146

Phenoxide
The number of imaginary frequencies: 0
Total Energy (M06/6-311G+(d,p))= -306.76850993
ZPE= -306.678655
Enthalpy= -306.672387

C -0.28635 1.21265 0.

C 1.10202 1.20101 0.

C 1.83103 0. 0.

C 1.10202 -1.20101 0.

C -0.28635 -1.21265 0.

C -1.08463 0. 0.

H -0.83192 2.15714 0.

H 1.64175 2.15246 0.

H 2.92018 0. 0.

H 1.64175 -2.15246 0.

H -0.83192 -2.15714 0.

O -2.35078 0. 0.

Phenoxide
The number of imaginary frequencies: 0
Total Energy (B3PW91/6-31G*)= -306.76798829
ZPE= -306.676996
Enthalpy= -306.6708

C 1.04882 -0.56995 0.00005

C 2.45022 -0.57002 0.00054

C 3.15099 0.64359 0.

C 2.45035 1.85727 -0.00103

C 1.04895 1.85735 -0.00151

C 0.34819 0.64374 -0.00097

H 0.51377 -1.49657 0.00046

H 2.98517 -1.4967 0.00131

H 4.22099 0.64353 0.00036

H 2.9854 2.78389 -0.00144

H 0.514 2.78402 -0.00229

O -1.08181 0.64381 -0.00146

Phenoxide
The number of imaginary frequencies: 0
Total Energy (B3PW91/6-31G**)= -306.77579656
ZPE= -306.68491
Enthalpy= -306.678712

C 1.04882 -0.56995 0.00005

C 2.45022 -0.57002 0.00054

C 3.15099 0.64359 0.

C 2.45035 1.85727 -0.00103

C 1.04895 1.85735 -0.00151

C 0.34819 0.64374 -0.00097

H 0.51377 -1.49657 0.00046

H 2.98517 -1.4967 0.00131

H 4.22099 0.64353 0.00036

H 2.9854 2.78389 -0.00144

H 0.514 2.78402 -0.00229

O -1.08181 0.64381 -0.00146

Phenoxide
The number of imaginary frequencies: 0
Total Energy (B3PW91/6-311G+(d,p))= -306.86725975
ZPE= -306.776989
Enthalpy= -306.770741

C 1.04882 -0.56995
0.00005

C 2.45022 -0.57002
0.00054

C 3.15099 0.64359 0.

C 2.45035 1.85727 -
0.00103

C 1.04895 1.85735 -
0.00151

C 0.34819 0.64374 -
0.00097

H 0.51377 -1.49657
0.00046

H 2.98517 -1.4967
0.00131

H 4.22099 0.64353
0.00036

H 2.9854 2.78389 -
0.00144

H 0.514 2.78402 -
0.00229

O -1.08181 0.64381 -
0.00146

Cyclohexane
The number of imaginary frequencies: 0
Total Energy (B3LYP/6-31G*)= -
235.88044327
ZPE= -235.709245
Enthalpy= -235.702607

C -0.68364 -0.89724 0.

C 0.83147 -0.89724 0.

C 1.3834 0.51383 0.

C 0.83374 1.31837
1.16066

C -0.68139 1.31903
1.16017

C -1.23419 -0.09159
1.15888

H 2.502 0.47984
0.06271

H 1.20402 -1.44288
0.90656

H 1.20676 -1.4471 -
0.90191

H -1.05633 -0.46422 -
0.96539

H -1.05923 -1.95146
0.0635

H 1.20627 0.88353
2.12527

H 1.20976 2.37251
1.09867

H -1.05671 1.86846
2.06229

H -1.05324 1.86563
0.25385

H -0.96818 -0.59731
2.12419

H -2.35274 -0.05653
1.09394

H 1.11478 1.01947 -
0.96455

Cyclohexane
The number of imaginary frequencies: 0
Total Energy (M06/6-311G+(d,p))= -
235.74170309
ZPE= -235.572734
Enthalpy= -235.566134

C 1.41187 -0.33689
0.23177

C 0.41394 -1.39013 -
0.23177

C -0.99755 -1.05372
0.23173

C -1.41187 0.33689 -
0.23177

C -0.41394 1.39013
0.23177

C 0.99755 1.05372 -
0.23173

H -1.70942 -1.80653 -
0.12723

H 0.42879 -1.43952 -
1.33177

H 0.70921 -2.38334
0.12701

H 1.46359 -0.34914
1.33171

H 2.41924 -0.57756 -
0.12822

H -1.46359 0.34914 -
1.33171

H -2.41924 0.57756
0.12822

H -0.70921 2.38334 -
0.12701

H -0.42879 1.43952
1.33177

H 1.03335 1.09098 -
1.33175

H 1.70942 1.80653
0.12723

H -1.03335 -1.09098
1.33175

Cyclohexane
The number of imaginary frequencies: 0
Total Energy (B3PW91/6-31G*)= -
235.79991158
ZPE= -235.628375
Enthalpy= -235.621737

C -0.68364 -0.89724 0.

C 0.83147 -0.89724 0.

C 1.3834 0.51383 0.

C 0.83374 1.31837
1.16066

C -0.68139 1.31903
1.16017

C -1.23419 -0.09159
1.15888

H 2.502 0.47984
0.06271

H 1.20402 -1.44288
0.90656

H 1.20676 -1.4471 -
0.90191

H -1.05633 -0.46422 -
0.96539

H -1.05923 -1.95146
0.0635

H 1.20627 0.88353
2.12527

H 1.20976 2.37251
1.09867

H -1.05671 1.86846
2.06229

H -1.05324 1.86563
0.25385

H -0.96818 -0.59731
2.12419

H -2.35274 -0.05653
1.09394

H 1.11478 1.01947 -
0.96455

Cyclohexane
The number of imaginary frequencies: 0
Total Energy (B3PW91/6-31G**)= -
235.81539469
ZPE= -235.644589
Enthalpy= -235.637903

C -0.52919 -1.36163
0.22935

C -1.44445 -0.22267 -
0.22939

C -0.91503 1.13895
0.2294

C 0.52919 1.36163 -
0.22934

C 1.44445 0.22267 0.2294

C 0.91502 -1.13895 -
0.22942

H -1.56141 1.94345 -
0.14206

H -1.50938 -0.23263 -
1.32715

H -2.46417 -0.37997
0.14279

H -0.55257 -1.42175
1.32714

H -0.90269 -2.32375 -
0.14193

H 0.5526 1.42177 -
1.32713

H 0.90269 2.32374
0.14196

H 2.46417 0.37997 -
0.14278

H 1.50937 0.2326
1.32716

H 0.95559 -1.18907 -
1.3272

H 1.56141 -1.94346 0.142

H -0.95563 1.18911
1.32717

Cyclohexane
The number of imaginary frequencies: 0
Total Energy (B3PW91/6-311G+(d,p))= -
235.8589444
ZPE= -235.689291
Enthalpy= -235.682583

C 0. -1.45193 0.25667

C -1.2574 -0.72596 -
0.25667

C -1.2574 0.72596
0.25667

C 0. 1.45193 -0.25667

C 1.2574 0.72596
0.25667

C 1.2574 -0.72596 -
0.25667

H -2.13106 1.23037 -0.1

H -1.2574 -0.72596 -
1.32667

H -2.13106 -1.23037 0.1

H 0. -1.45193 1.32667

H 0. -2.46073 -0.1

H 0. 1.45193 -1.32667

H 0. 2.46073 0.1

H 2.13106 1.23037 -0.1

H 1.2574 0.72596
1.32667

H 1.2574 -0.72596 -
1.32667

H 2.13106 -1.23037 0.1

H -1.2574 0.72596
1.32667

Cyclohexene
The number of imaginary frequencies: 0
Total Energy (B3LYP/6-31G*)= -
234.64828834
ZPE= -234.501263
Enthalpy= -234.494842

C -1.09705 -2.25773 -
0.09838

C 0.41779 -2.25199
0.12713

C 0.93262 -0.82852 -
0.15134

C 0.15985 0.21534
0.68073

C -1.11685 -0.013 1.0939

C -1.74518 -1.41963
1.01803

H 1.97685 -0.77566
0.07599

H 0.6251 -2.54628
1.13475

H 0.90114 -2.92981 -
0.54505

H -1.31126 -1.84627 -
1.06259

H -1.48166 -3.25488 -
0.0468

H 0.63273 1.14297
0.92728

H -1.69215 0.79701
1.49117

H -1.57375 -1.91002
1.95346

H -2.79722 -1.34005
0.83977

H 0.77709 -0.60796 -
1.18675

Cyclohexene
The number of imaginary frequencies: 0
Total Energy (M06/6-311G+(d,p))= -
234.51167786
ZPE= -234.366563
Enthalpy= -234.360136

C -0.37174 -0.67134 -
1.19224

C 0.37174 0.67134 -
1.19224

C 0.00551 1.50293
0.04786

C -0.00551 0.66851
1.3061

C 0.00551 -0.66851
1.3061

C -0.00551 -1.50293
0.04786

H 0.70963 2.33869
0.16382

H 1.45443 0.48193 -
1.19273

H 0.15032 1.23633 -
2.10602

H -1.45443 -0.48193 -
1.19273

H -0.15032 -1.23633 -
2.10602

H -0.01996 1.20449
2.25474

H 0.01996 -1.20449
2.25474

H 0.98274 -1.97072 -
0.09014

H -0.70963 -2.33869
0.16382

H -0.98274 1.97072 -
0.09014

1,3-Cyclohexadiene
The number of imaginary frequencies: 0
Total Energy (B3LYP/6-31G*)= -
233.41893221
ZPE= -233.296105
Enthalpy= -233.289987

C -0.15118 0.13054
0.24939

C 1.32754 0.10935 -
0.18631

C 2.02124 1.27034 -
0.27831

C 1.35327 2.58323
0.16487

C -0.13997 2.58558 -
0.21611

C -0.82738 1.3048
0.28723

H -0.64641 -0.78146 0.51

H 1.80897 -0.82106 -
0.40418

H 3.03042 1.27026 -
0.63391

H 1.4551 2.66525
1.22685

H -0.60147 3.44849
0.2167

H -1.83632 1.33633
0.6421

H 1.82749 3.41813 -
0.30731

H -0.24093 2.6198 -
1.28078

1,3-Cyclohexadiene
The number of imaginary frequencies: 0
Total Energy (M06/6-311G+(d,p))= -
233.28434875
ZPE= -233.162923
Enthalpy= -233.15679

C -0.7637 0.10482
1.26813

C 0.7637 -0.10482
1.26813

C 1.45068 -0.06654
0.10016

C 0.7097 0.3001 -
1.19706

C -0.7097 -0.3001 -
1.19706

C -1.45068 0.06654
0.10016

H -1.28239 0.26836
2.18961

H 1.28239 -0.26836
2.18961

H 2.50164 -0.26688
0.08443

H 0.65063 1.36689 -
1.25519

H -1.2401 0.0774 -
2.04622

H -2.50164 0.26688
0.08443

H 1.2401 -0.0774 -
2.04622

H -0.65063 -1.36689 -
1.25519

Cyclohexanol
The number of imaginary frequencies: 0
Total Energy (B3LYP/6-31G*)= -
311.09023047
ZPE= -310.914856
Enthalpy= -310.907074

C -1.69842 -0.9235 -
0.03227

C -0.16061 -0.92136
0.04987

C 0.35182 0.53086
0.04271

C -0.22796 1.28577
1.25332

C -1.76576 1.28364
1.17118

C -2.2782 -0.16859
1.17833

H 1.4203 0.53234
0.09979

H 0.14899 -1.40438
0.95306

H 0.24222 -1.44588 -
0.79126

H -2.00802 -0.44048 -
0.93546

H -2.05446 -1.93251 -
0.0273

H 0.08164 0.80275
2.15651

H -2.1686 1.80815
2.01231

H -2.07536 1.76666
0.26799

H -1.9686 -0.6516
2.08152

H -3.34667 -0.17007
1.12126

H 0.04222 1.01388 -
0.86047

O 0.24788 2.63427
1.24667

H 1.20651 2.6356
1.29788

Cyclohexanol
The number of imaginary frequencies: 0
Total Energy (M06/6-311G+(d,p))= -
310.9561851
ZPE= -310.782442
Enthalpy= -310.774714

C 1.87188 0.015 -0.27822

C 1.14616 1.27958
0.20746

C -0.34037 1.25452 -
0.18469

C -1.03808 -0.01488
0.32185

C -0.31932 -1.2696 -
0.17361

C 1.16738 -1.25843
0.21595

H -0.85392 2.14483
0.20746

H 1.23313 1.34863
1.30195

H 1.62713 2.17808 -
0.19869

H 1.89506 0.01191 -
1.37782

H 2.91685 0.02568
0.05675

H -1.01273 -0.01168
1.42704

H -0.82456 -2.15708
0.22535

H -0.42203 -1.30947 -
1.26703

H 1.25839 -1.31957
1.31058

H 1.66502 -2.14978 -
0.18566

H -0.44264 1.28377 -
1.27849

O -2.38974 -0.09818 -
0.13038

H -2.84767 0.70295
0.16909

Cyclohexanol
The number of imaginary frequencies: 0
Total Energy (B3PW91/6-31G*)= -
310.98134763
ZPE= -310.805554
Enthalpy= -310.797773

C -1.69842 -0.9235 -
0.03227

C -0.16061 -0.92136
0.04987

C 0.35182 0.53086
0.04271

C -0.22796 1.28577
1.25332

C -1.76576 1.28364
1.17118

C -2.2782 -0.16859
1.17833

H 1.4203 0.53234
0.09979

H 0.14899 -1.40438
0.95306

H 0.24222 -1.44588 -
0.79126

H -2.00802 -0.44048 -
0.93546

H -2.05446 -1.93251 -
0.0273

H 0.08164 0.80275
2.15651

H -2.1686 1.80815
2.01231

H -2.07536 1.76666
0.26799

H -1.9686 -0.6516
2.08152

H -3.34667 -0.17007
1.12126

H 0.04222 1.01388 -
0.86047

O 0.24788 2.63427
1.24667

H 1.20651 2.6356
1.29788

Cyclohexanol
The number of imaginary frequencies: 0
Total Energy (B3PW91/6-31G**)= -
311.00115199
ZPE= -310.825835
Enthalpy= -310.818032

C -1.69842 -0.9235 -
0.03227

C -0.16061 -0.92136
0.04987

C 0.35182 0.53086
0.04271

C -0.22796 1.28577
1.25332

C -1.76576 1.28364
1.17118

C -2.2782 -0.16859
1.17833

H 1.4203 0.53234
0.09979

H 0.14899 -1.40438
0.95306

H 0.24222 -1.44588 -
0.79126

H -2.00802 -0.44048 -
0.93546

H -2.05446 -1.93251 -
0.0273

H 0.08164 0.80275
2.15651

H -2.1686 1.80815
2.01231

H -2.07536 1.76666
0.26799

H -1.9686 -0.6516
2.08152

H -3.34667 -0.17007
1.12126

H 0.04222 1.01388 -
0.86047

O 0.24788 2.63427
1.24667

H 1.20651 2.6356
1.29788

Cyclohexanol
The number of imaginary frequencies: 0
Total Energy (B3PW91/6-311G+(d,p))= -311.07296237
ZPE= -310.89868
Enthalpy= -310.890825

C -1.69842 -0.9235 -
0.03227

C -0.16061 -0.92136
0.04987

C 0.35182 0.53086
0.04271

C -0.22796 1.28577
1.25332

C -1.76576 1.28364
1.17118

C -2.2782 -0.16859
1.17833

H 1.4203 0.53234
0.09979

H 0.14899 -1.40438
0.95306

H 0.24222 -1.44588 -
0.79126

H -2.00802 -0.44048 -
0.93546

H -2.05446 -1.93251 -
0.0273

H 0.08164 0.80275
2.15651

H -2.1686 1.80815
2.01231

H -2.07536 1.76666
0.26799

H -1.9686 -0.6516
2.08152

H -3.34667 -0.17007
1.12126

H 0.04222 1.01388 -
0.86047

O 0.24788 2.63427
1.24667

H 1.20651 2.6356
1.29788

Cyclohexanoxide
The number of imaginary frequencies: 0
Total Energy (B3LYP/6-31G*)= -310.46048432
ZPE= -310.301528
Enthalpy= -310.294035

C -2.17913 -2.26853 -
0.02636

C -0.64001 -2.29336
0.01911

C -0.10181 -0.85115 -
0.02535

C -0.63853 -0.06539
1.18548

C -2.17766 -0.04057
1.14002

C -2.71585 -1.48278
1.18448

H 0.96759 -0.8684
0.00624

H -0.31747 -2.76671
0.92288

H -0.26709 -2.83931 -
0.82218

H -2.50167 -1.79518 -
0.93013

H -2.55308 -3.27059
0.00453

H -0.31599 -0.53875
2.08926

H -2.55057 0.50538
1.98131

H -2.50019 0.43279
0.23624

H -2.39332 -1.95613
2.08825

H -3.78525 -1.46553 1.15288

H -0.42434 -0.3778 -0.92912

O -0.13877 1.2738 1.1442

Cycclohexanoxide
The number of imaginary frequencies: 0
Total Energy (M06/6-311G+(d,p))= -310.34864084
ZPE= -310.190974
Enthalpy= -310.183555

C 0.74639 -1.69549 0.

C 0.08897 -1.13045 1.27017

C 0.08897 0.40692 1.26393

C -0.59753 1.06331 0.

C 0.08897 0.40692 -1.26393

C 0.08897 -1.13045 -1.27017

H -0.41344 0.80182 2.15935

H -0.95188 -1.49063 1.31339

H 0.59165 -1.54105 2.1635

H 1.81428 -1.42266 0.

H 0.70757 -2.79762 0.

H -1.64779 0.56877 0.

H -0.41344 0.80182 -2.15935

H 1.12539 0.78331 -1.29784

H -0.95188 -1.49063 -1.31339

H 0.59165 -1.54105 -2.1635

H 1.12539 0.78331 1.29784

O -0.57575 2.37751 0.

Cyclohexanoxide
The number of imaginary frequencies: 0
Total Energy (B3PW91/6-31G*)= -310.35127286
ZPE= -310.191988
Enthalpy= -310.184495

C -2.17913 -2.26853 -0.02636

C -0.64001 -2.29336 0.01911

C -0.10181 -0.85115 -0.02535

C -0.63853 -0.06539 1.18548

C -2.17766 -0.04057 1.14002

C -2.71585 -1.48278 1.18448

H 0.96759 -0.8684 0.00624

H -0.31747 -2.76671 0.92288

H -0.26709 -2.83931 -0.82218

H -2.50167 -1.79518 -0.93013

H -2.55308 -3.27059 0.00453

H -0.31599 -0.53875 2.08926

H -2.55057 0.50538 1.98131

H -2.50019 0.43279 0.23624

H -2.39332 -1.95613 2.08825

H -3.78525 -1.46553 1.15288

H -0.42434 -0.3778 -0.92912

O -0.13877 1.2738 1.1442

Cyclohexanoxide
The number of imaginary frequencies: 0
Total Energy (B3PW91/6-31G**)= -310.36598991
ZPE= -310.20731
Enthalpy= -310.199792

C -2.17913 -2.26853 -0.02636

C -0.64001 -2.29336 0.01911

C -0.10181 -0.85115 -0.02535

C -0.63853 -0.06539 1.18548

C -2.17766 -0.04057 1.14002

C -2.71585 -1.48278 1.18448

H 0.96759 -0.8684 0.00624

H -0.31747 -2.76671 0.92288

H -0.26709 -2.83931 -0.82218

H -2.50167 -1.79518 -0.93013

H -2.55308 -3.27059 0.00453

H -0.31599 -0.53875 2.08926

H -2.55057 0.50538 1.98131

H -2.50019 0.43279 0.23624

H -2.39332 -1.95613 2.08825

H -3.78525 -1.46553 1.15288

H -0.42434 -0.3778 -0.92912

O -0.13877 1.2738 1.1442

Cyclohexanoxide
The number of imaginary frequencies: 0
Total Energy (B3PW91/6-311G+(d,p))= -310.46013267
ZPE= -310.302065
Enthalpy= -310.294548

C -2.17913 -2.26853 -0.02636

C -0.64001 -2.29336 0.01911

C -0.10181 -0.85115 -0.02535

C -0.63853 -0.06539 1.18548

C -2.17766 -0.04057 1.14002

C -2.71585 -1.48278 1.18448

H 0.96759 -0.8684 0.00624

H -0.31747 -2.76671 0.92288

H -0.26709 -2.83931 -0.82218

H -2.50167 -1.79518 -0.93013

H -2.55308 -3.27059 0.00453

H -0.31599 -0.53875
2.08926

H -2.55057 0.50538
1.98131

H -2.50019 0.43279
0.23624

H -2.39332 -1.95613
2.08825

H -3.78525 -1.46553
1.15288

H -0.42434 -0.3778 -
0.92912

O -0.13877 1.2738 1.1442

1,3-Cyclohexadiene-1-ol ion
The number of imaginary frequencies: 0
Total Energy (B3LYP/6-31G*)= -
308.06288455
ZPE= -307.950009
Enthalpy= -307.943075

C -2.39644 -0.99279 -
0.08473

C -1.18737 -0.65562 -
0.59663

C -0.40603 0.53315 -
0.00229

C -1.02541 1.39408
0.84195

C -2.53292 1.23688
1.10459

C -2.91972 -0.25388
1.15883

H -0.77084 -1.22108 -
1.40388

H -2.97031 -1.77998 -
0.52735

H -2.79622 1.69212
2.03643

H -3.06045 1.71802
0.30765

H -2.48319 -0.70895
2.02327

H -3.98575 -0.32833
1.21295

H 0.61999 0.68539 -
0.26497

O -0.29137 2.45555
1.45789

1,3-Cyclohexadiene-1-ol ion
The number of imaginary frequencies: 0
Total Energy (M06/6-311G+(d,p))= -
307.94858474
ZPE= -307.836671
Enthalpy= -307.829759

C -2.39644 -0.99279 -
0.08473

C -1.18737 -0.65562 -
0.59663

C -0.40603 0.53315 -
0.00229

C -1.02541 1.39408
0.84195

C -2.53292 1.23688
1.10459

C -2.91972 -0.25388
1.15883

H -0.77084 -1.22108 -
1.40388

H -2.97031 -1.77998 -
0.52735

H -2.79622 1.69212
2.03643

H -3.06045 1.71802
0.30765

H -2.48319 -0.70895
2.02327

H -3.98575 -0.32833 1.21295

H 0.61999 0.68539 -0.26497

O -0.29137 2.45555 1.45789

1,5-Cyclohexadiene-1-ol ion
The number of imaginary frequencies: 0
Total Energy (B3LYP/6-31G*)= -308.04298519
ZPE= -307.930465
Enthalpy= -307.92353

C -2.51853 -0.72365 -0.12507

C -0.99771 -0.63793 -0.33961

C -0.3566 0.52872 -0.08372

C -1.13237 1.71417 0.52438

C -2.37016 1.51757 1.04089

C -2.93616 0.08918 1.11582

H -0.45518 -1.48316 -0.70859

H -2.99914 -0.32717 -0.99497

H -2.81591 -1.74156 0.0175

H -2.93799 2.34444 1.41338

H -2.54907 -0.37516 1.99868

H -4.0048 0.11596 1.16272

H 0.68325 0.63038 -0.31455

O -0.54097 3.01572 0.55806

1,5-Cyclohexadiene-1-ol ion
The number of imaginary frequencies: 0
Total Energy (M06/6-311G+(d,p))= -307.92956598
ZPE= -307.818063
Enthalpy= -307.811119

C -2.51853 -0.72365 -0.12507

C -0.99771 -0.63793 -0.33961

C -0.3566 0.52872 -0.08372

C -1.13237 1.71417 0.52438

C -2.37016 1.51757 1.04089

C -2.93616 0.08918 1.11582

H -0.45518 -1.48316 -0.70859

H -2.99914 -0.32717 -0.99497

H -2.81591 -1.74156 0.0175

H -2.93799 2.34444 1.41338

H -2.54907 -0.37516 1.99868

H -4.0048 0.11596 1.16272

H 0.68325 0.63038 -0.31455

O -0.54097 3.01572 0.55806

H_2 Gas
The number of imaginary frequencies: 0
Total Energy (B3LYP/6-31G*)= -1.17548239

ZPE= -1.165337
Enthalpy= -1.162033

H	0.	0.	0.37139
H	0.	0.	-0.37139

H_2 Gas
The number of imaginary frequencies: 0
Total Energy (M06/6-311G+(d,p))= -1.17039476
ZPE=-1.160564
Enthalpy=-1.157260

H	0.	0.	0.37139
H	0.	0.	-0.37139

H_2 Gas
The number of imaginary frequencies: 0
Total Energy (B3PW91/6-31G*)= -1.17456185
ZPE= -1.164426
Enthalpy= -1.161121

H	0.	0.	0.37139
H	0.	0.	-0.37139

H_2 Gas
The number of imaginary frequencies: 0
Total Energy (B3PW91/6-31G**)= -1.17751625
ZPE= -1.167344
Enthalpy= -1.16404

H	0.	0.	0.37139
H	0.	0.	-0.37139

H_2 Gas
The number of imaginary frequencies: 0
Total Energy (B3PW91/6-311G+(d,p))= -1.17857658
ZPE= -1.168516
Enthalpy= -1.165212

H	0.	0.	0.37268
H	0.	0.	-0.37268

Triplet Oxygen, ^{3}O
The number of imaginary frequencies: 0
Total Energy (B3LYP/6-31G*)= -75.06062312
ZPE= -75.060623
Enthalpy= -75.058263
O 0. 0. 0.

Triplet Oxygen, ^{3}O
The number of imaginary frequencies: 0
Total Energy (B3LYP/6-31G**)= -75.06062143
ZPE= -75.060621
Enthalpy= -75.058261
O 0. 0. 0.

Triplet Oxygen, ^{3}O
The number of imaginary frequencies: 0
Total Energy (M06/6-311G+(d,p))= -75.05344297
ZPE= -75.053443
Enthalpy= -75.051083
O 0. 0. 0.

Triplet Oxygen, ^{3}O
The number of imaginary frequencies: 0
Total Energy (B3PW91/6-31G*)= -75.03133311
ZPE= -75.031333
Enthalpy= -75.028973
O 0. 0. 0.

Triplet Oxygen, ^{3}O
The number of imaginary frequencies: 0
Total Energy (B3PW91/6-31G**)= -75.03133311
ZPE= -75.031333
Enthalpy= -75.028973
O 0. 0. 0.

Triplet Oxygen, ^{3}O
The number of imaginary frequencies: 0
Total Energy (B3PW91/6-311G+(d,p))= -75.05845466
ZPE= -75.058455
Enthalpy= -75.056094
O 0. 0. 0.

Singlet Oxygen, ^{1}O
The number of imaginary frequencies: 0

Total Energy (B3LYP/6-31G*)= -74.95739789
ZPE= -74.957398
Enthalpy= -74.955037
O 0. 0. 0.

Singlet Oxygen, [1]O
The number of imaginary frequencies: 0
Total Energy (M06/6-311G+(d,p))= -74.95543188
ZPE= -74.955432
Enthalpy= -74.953071

O 0. 0. 0.

Carbon Monoxide
The number of imaginary frequencies: 0
Total Energy (B3LYP/6-31G*)= -113.30945434
ZPE= -113.304423
Enthalpy= -113.301118

C -0.68024 0.02465 0.12843

O -0.30843 0.55045 1.03916

Carbon Monoxide
The number of imaginary frequencies: 0
Total Energy (M06/6-311G+(d,p))= -113.28594104
ZPE= -113.280844
Enthalpy= -113.277539

C 0. 0. -0.65026

O 0. 0. 0.48769

Carbon Dioxide
The number of imaginary frequencies: 0
Total Energy (B3LYP/6-31G*)= -188.58094022
ZPE= -188.569347
Enthalpy= -188.565756

C 1.4248 0.55144 0.3892

O 2.45228 0.9147 1.0184

O 0.39732 0.18818 -0.24

Carbon Dioxide
The number of imaginary frequencies: 0
Total Energy (M06/6-311G+(d,p))= -188.5592754
ZPE= -188.547202
Enthalpy= -188.543659
C 0. 0. 0.

O 0. 0. 1.16916

O 0. 0. -1.16916

Nitric Oxide
The number of imaginary frequencies: 0
Total Energy (B3LYP/6-31G*)= -129.8881562
ZPE= -129.88362
Enthalpy= -129.880315

O 1.3391 0.28484 0.

N 0.9396 1.41299 0.

Nitric Oxide
The number of imaginary frequencies: 0
Total Energy (M06/6-311G+(d,p))= -129.85739211
ZPE= -129.852775
Enthalpy= -129.84947
O 0. 0. 0.54073

N 0. 0. -0.61797

Nitrogen Dioxide
The number of imaginary frequencies: 0
Total Energy (B3LYP/6-31G*)= -205.07220619
ZPE= -205.06338
Enthalpy= -205.059499
N -0.62902 0.26164 -0.07844

O -0.27177 -0.91919 0.0199

O -0.27175 0.93721 -1.05189

Nitrogen Dioxide
The number of imaginary frequencies: 0

Total Energy (M06/6-311G+(d,p))= -205.03467527
ZPE= -205.025534
Enthalpy= -205.021663

N 0. 0. 0.32802

O 0. 1.10685 -0.14351

O 0. -1.10685 -0.14351

$^3O_2$, Triplet
The number of imaginary frequencies: 0
Total Energy (B3LYP/6-31G*)= -150.32004208
ZPE= -150.316263
Enthalpy= -150.312956

O -1.80199 0.58416 0.

O -0.64039 0.58416 0.

$^3O_2$, Triplet
The number of imaginary frequencies: 0
Total Energy (M06/6-311G+(d,p))= -150.29135144
ZPE= -150.287488
Enthalpy= -150.284182

O 0. 0. 0.6073

O 0. 0. -0.6073

$^1O_2$, Singlet
The number of imaginary frequencies: 0
Total Energy (B3LYP/6-31G*)= -150.25742664
ZPE= -150.253684
Enthalpy= -150.250376

O -0.83169 2.06931 0.

O 0.32991 2.06931 0.

$^1O_2$, Singlet
The number of imaginary frequencies: 0
Total Energy (M06/6-311G+(d,p))= -150.2320003
ZPE= -150.228126
Enthalpy= -150.22482

O 0. 0. 0.608

O 0. 0. -0.608

Bicyclo[1.1.0]butane
The number of imaginary frequencies: 0
Total Energy (B3LYP/6-31G*)= -155.94803158
ZPE= -155.861217
Enthalpy= -155.856544

C -1.29743 0.01091 0.7812

C -0.00313 -0.00913 -0.18404

C -1.03745 -1.13365 -0.20384

H -0.93566 -0.08314 1.78379

H -1.67228 -1.18996 -1.06333

H -0.83659 -2.14254 0.09058

C -1.02504 1.13205 -0.22061

H -1.668 1.18268 -1.07439

H -0.80332 2.14282 0.05157

H 0.85437 0.09034 0.44817

Bicyclo[1.1.0]butane
The number of imaginary frequencies: 0
Total Energy (M06/6-311G+(d,p))= -155.87025179
ZPE= -155.784497
Enthalpy= -155.779812

C -0.80733 -0.00656 -0.31956

C 0.80733 0.00656 -0.31956

C -0.01329 1.13282 0.31767

H -1.11469 -0.10966 -1.33926

H -0.01935 1.18618 1.38632

H -0.01902 2.14275 -0.03575

C 0.01329 -1.13282 0.31767

H 0.01935 -1.18618 1.38632

H 0.01902 -2.14275 -0.03575

H 1.11469 0.10966 -1.33926

1-cyano-Bicyclo[1.1.0]butane
The number of imaginary frequencies: 0
Total Energy (B3LYP/6-31G*)= -248.19372057
ZPE= -248.107661
Enthalpy= -248.101465

C -1.17456 0.00083 0.72459

C -0.0488 -0.00079 -0.28293

C -1.03706 -1.14061 -0.22917

H -1.08061 0.00069 1.80143

H -1.74718 -1.2416 -1.05321

H -0.67545 -2.08454 0.174

C -1.03427 1.14166 -0.22941

H -1.74456 1.24418 -1.0531

H -0.67019 2.08473 0.17352

C 1.35403 -0.00132 -0.0764

N 2.50885 -0.00029 0.0739

1-cyano-Bicyclo[1.1.0]butane
The number of imaginary frequencies: 0
Total Energy (M06/6-311G+(d,p))= -248.08154884
ZPE= -247.996309
Enthalpy= -247.990121

C -1.14802 0.00005 0.72613

C -0.05352 -0.00003 -0.2782

C -1.0329 -1.13224 -0.22727

H -1.04334 0.00004 1.80049

H -1.75195 -1.21366 -1.0438

H -0.66789 -2.0771 0.1664

C -1.03276 1.1323 -0.22729

H -1.75181 1.21381 -1.04381

H -0.66762 2.07713 0.16637

C 1.34359 -0.00015 -0.08058

N 2.48918 0.00003 0.06821

Cyclobutane
The number of imaginary frequencies: 1 (-136.39 cm^{-1})
Total Energy (B3LYP/6-31G*)= -157.21183323
ZPE= -157.100818
Enthalpy= -157.096326

C 0.43478 0.49407 0.

C 1.97803 0.49407 0.

C 1.97797 2.03726 0.

C 0.43474 2.03726 -0.00027

H -0.01821 0.04096 -0.90642

H -0.01788 0.04111 0.90664

H 2.43094 0.04126 -0.90662

H 2.43091 0.04102 0.90653

H 2.43089 2.49002 -0.90666

H 2.43069 2.49039 0.9065

H -0.01796 2.48979 -0.90724

H -0.01853 2.49052 0.90596

Cyclobutane
The number of imaginary frequencies: 1 (-169.56 cm^{-1})
Total Energy (M06/6-311G+(d,p))= -157.11692129
ZPE= -157.007394
Enthalpy= -157.002855

C -0.07615 1.09848 -0.00016

C 1.09849 0.07615 -0.00005

C 0.07615 -1.09848 -0.00007

C -1.09848 -0.07615 0.00028

H -0.12071 1.74204 0.88428

H -0.12091 1.74147 -0.885

H 1.74166 0.12083 0.88465

H 1.74184 0.1208 -0.88464

H 0.12095 -1.74189 0.88446

H 0.12067 -1.7416 -0.88483

H -1.74136 -0.12076 0.88521

H -1.74215 -0.12087 -0.88407

1-cyano-cyclobutane
The number of imaginary frequencies: 0
Total Energy (B3LYP/6-31G*)= -249.45559746
ZPE= -249.345159
Enthalpy= -249.338545

C -0.07615 1.09848 -0.00016

C 1.09849 0.07615 -0.00005

C 0.07615 -1.09848 -0.00007

C -1.09848 -0.07615 0.00028

H -0.12091 1.74147 -0.885

H 1.74166 0.12083 0.88465

H 1.74184 0.1208 -0.88464

H 0.12095 -1.74189 0.88446

H 0.12067 -1.7416 -0.88483

H -1.74136 -0.12076 0.88521

H -1.74215 -0.12087 -0.88407

C -0.13884 2.00382 1.24405

N -0.18606 2.68576 2.18124

1-cyano-cyclobutane
The number of imaginary frequencies: 0
Total Energy (M06/6-311G+(d,p))= -249.32981858
ZPE= -249.220632

Enthalpy= -249.214045

C -0.40578 0.16243 0.

C 0.20369 -0.79289 -1.04213

C -0.07507 -1.89255 0.

C 0.20369 -0.79289 1.04213

H -1.47442 0.10829 0.

H 1.2533 -0.62346 -1.16275

H -0.19232 -0.85683 -2.0341

H 0.47817 -2.80841 0.

H -1.10581 -2.17976 0.

H 1.2533 -0.62346 1.16275

H -0.19232 -0.85683 2.0341

C -0.1018 1.53058 0.

N 0.14765 2.64963 0.

Isobutane
The number of imaginary frequencies: 0
Total Energy (B3LYP/6-31G*)= -158.45880816
ZPE= -158.326386
Enthalpy= -158.319765

C 0. 1.46175 -0.09598

C 0. 0. 0.37309

H 0. 0. 1.47365

H -0.88629 1.99733 0.26549

H 0.88629 1.99733 0.26549

H 0. 1.52015 -1.19253

C 1.26592 -0.73088 -0.09598

C -1.26592 -0.73088 -0.09598

H 1.28659 -1.76622 0.26549

H 1.31649 -0.76007 -1.19253

H 2.17288 -0.23111 0.26549

H -1.31649 -0.76007 -1.19253

H -1.28659 -1.76622 0.26549

H -2.17288 -0.23111 0.26549

Isobutane
The number of imaginary frequencies: 0
Total Energy (M06/6-311G+(d,p))= -158.36042407
ZPE= -158.229965
Enthalpy= -158.223369

C -0.8703 1.17835 0.

C 0.31399 0.20152 0.

H 1.24021 0.79595 0.

H -0.85537 1.82432 0.88629

H -0.85537 1.82432 -0.88629

H -1.82469 0.63522 0.

C 0.31399 -0.66693 -1.26592

C 0.31399 -0.66693 1.26592

H 1.17741 -1.34303 -1.28659

H -0.59309 -1.28378 -1.31649

H 0.34826 -0.0511 -2.17288

H -0.59309 -1.28378
1.31649

H 1.17741 -1.34303
1.28659

H 0.34826 -0.0511
2.17288

2-cyano-isobutane
The number of imaginary frequencies: 0
Total Energy (B3LYP/6-31G*)= -
250.69730861
ZPE= -250.566261
Enthalpy= -250.558043

C 0. 1.46175 -0.78112

C 0. 0. -0.31205

H -0.88629 1.99733 -
0.41965

H 0.88629 1.99733 -
0.41965

H 0. 1.52015 -1.87768

C 1.26591 -0.73088 -
0.78112

C -1.26591 -0.73088 -
0.78112

H 1.28659 -1.76622 -
0.41965

H 1.31649 -0.76007 -
1.87768

H 2.17288 -0.23111 -
0.41965

H -1.31649 -0.76007 -
1.87768

H -1.28659 -1.76622 -
0.41965

H -2.17288 -0.23111 -
0.41965

C 0. 0.
1.22795

N 0. 0.
2.38795

2-cyano-isobutane
The number of imaginary frequencies: 0
Total Energy (M06/6-311G+(d,p))= -
250.56898547
ZPE= -250.439709
Enthalpy= -250.431521

C 0. 1.46469 -0.77277

C 0. 0. -0.27937

H -0.88699 2.00042 -
0.42021

H 0.88699 2.00042 -
0.42021

H 0. 1.48094 -
1.86848

C 1.26846 -0.73234 -
0.77277

C -1.26846 -0.73234 -
0.77277

H 1.28892 -1.76836 -
0.42021

H 1.28253 -0.74047 -
1.86848

H 2.17591 -0.23206 -
0.42021

H -1.28253 -0.74047 -
1.86848

H -1.28892 -1.76836 -
0.42021

H -2.17591 -0.23206 -
0.42021

C 0. 0.
1.19869

N 0. 0. 2.36008

Isobutane Oxide
The number of imaginary frequencies: 0
Total Energy (B3LYP/6-31G*)= -
233.0061692
ZPE= -232.883092

Enthalpy= -232.875636

C -0.08465 0.42439
0.00229

C 1.45504 0.44006 -
0.02467

H -0.45153 1.42951
0.00782

H -0.45139 -0.0844 -
0.86462

H -0.42081 -0.08284
0.88241

C 1.98307 -1.00656 -
0.03263

C 1.98287 1.17233
1.22304

H 3.05285 -0.99567 -
0.05136

H 1.6469 -1.51379
0.84749

H 1.61632 -1.51535 -
0.89954

H 1.64671 0.6651
2.10316

H 3.05265 1.18323
1.2043

H 1.61599 2.17746
1.22856

O 1.9043 1.11795 -
1.20091

Isobutane Oxide
The number of imaginary frequencies: 0
Total Energy (M06/6-311G+(d,p))= -
232.90163743
ZPE= -232.780778
Enthalpy= -232.773277

C -1.48801 0.2227 0.

C 0.06425 0.05546 0.

H -1.81464 0.76608
0.89058

H -1.81464 0.76608 -
0.89058

H -1.94366 -0.77347 0.

C 0.5156 -0.67984 -
1.27764

C 0.5156 -0.67984
1.27764

H 1.60844 -0.74996 -
1.30523

H 0.10776 -1.69648 -
1.31582

H 0.1856 -0.13249 -
2.16599

H 0.10776 -1.69648
1.31582

H 1.60844 -0.74996
1.30523

H 0.1856 -0.13249
2.16599

O 0.5156 1.36104 0.

Tert-butoxide
The number of imaginary frequencies: 0
Total Energy (B3LYP/6-31G*)= -
233.03927664
ZPE= -232.919
Enthalpy= -232.911557

C -2.95594 -0.07066
0.00229

C -1.41625 -0.05499 -
0.02467

H -3.32281 0.93446
0.00782

H -3.32268 -0.57945 -
0.86462

H -3.2921 -0.57789
0.88241

C -0.88822 -1.50161 -
0.03263

C -0.88842 0.67728
1.22304

H 0.18156 -1.49072 -
0.05136

H -1.22438 -2.00884
0.84749

H -1.25496 -2.0104 -
0.89954

H -1.22458 0.17005
2.10316

H 0.18136 0.68818
1.2043

H -1.2553 1.68241
1.22856

O -0.96699 0.6229 -
1.20091

Tert-butoxide
The number of imaginary frequencies: 0
Total Energy (M06/6-311G+(d,p))= -
232.96980782
ZPE= -232.850251
Enthalpy= -232.843033

C 0. 1.46319 -0.43682

C 0. 0. 0.15961

H -0.88471 1.99423 -
0.05736

H 0.88471 1.99423 -
0.05736

H 0. 1.53306 -1.54093

C 1.26716 -0.7316 -
0.43682

C -1.26716 -0.7316 -
0.43682

H 1.2847 -1.7633 -
0.05736

H 1.32767 -0.76653 -
1.54093

H 2.16941 -0.23093 -
0.05736

H -1.32767 -0.76653 -
1.54093

H -1.2847 -1.7633 -
0.05736

H -2.16941 -0.23093 -
0.05736

O 0. 0. 1.484

Tert-butanol
The number of imaginary frequencies: 0
Total Energy (B3LYP/6-31G*)= -
233.67097108
ZPE= -233.53469
Enthalpy= -233.527061

C -2.78614 0.77678
0.00423

C -1.24647 0.7924 -0.0232

H -3.15299 1.78192
0.00918

H -3.15316 0.26742 -
0.86222

H -3.12206 0.27017
0.8848

C -0.71848 -0.65424 -
0.03032

C -0.71825 1.52551
1.22385

H 0.3513 -0.64339 -
0.04937

H -1.0544 -1.16086
0.85025

H -1.08549 -1.16361 -
0.89677

H -1.05416 1.01889
2.10442

H 0.35153 1.53636
1.2048

H -1.08509 2.53065
1.2288

O -0.79753 1.46947 -
1.20003

H -1.12681 1.01247 -
1.97741

Tert-butanol
The number of imaginary frequencies: 0
Total Energy (M06/6-311G+(d,p))= -
233.57822574
ZPE= -233.443543
Enthalpy= -233.435882

C -0.48647 -0.70717
1.26567

C 0.00063 0.01449 0.

H -0.16536 -0.16219
2.1595

H -1.58284 -0.77034
1.28057

H -0.09595 -1.72997
1.3225

C -0.48647 -0.70717 -
1.26567

C 1.52516 0.14866 0.

H -0.16536 -0.16219 -
2.1595

H -0.09595 -1.72997 -
1.3225

H -1.58284 -0.77034 -
1.28057

H 2.00594 -0.83553 0.

H 1.85667 0.6993 -
0.88647

H 1.85667 0.6993
0.88647

O -0.48647 1.36797 0.

H -1.45634 1.32533 0.

Dimethyldioxirane
The number of imaginary frequencies: 0
Total Energy (B3LYP/6-31G*)= -
268.26893635
ZPE= -268.180245
Enthalpy= -268.173668

C 0. 0. 0.09405

O -0.75265 0. -1.09014

O 0.75265 0. -1.09014

C 0. 1.29372 0.87204

H -0.88605 1.35345
1.51402

H 0.88605 1.35345
1.51402

H 0. 2.13721 0.17866

C 0. -1.29372 0.87204

H 0.88605 -1.35345
1.51402

H -0.88605 -1.35345
1.51402

H 0. -2.13721 0.17866

Diemthyldioxirane
The number of imaginary frequencies: 0
Total Energy (M06/6-311G+(d,p))= -
268.17073042
ZPE= -268.083194
Enthalpy= -268.076595

C 0. 0. 0.09405

O -0.75265 0. -1.09014

O 0.75265 0. -1.09014

C 0. 1.29372 0.87204

H -0.88605 1.35345
1.51402

H 0.88605 1.35345
1.51402

H 0. 2.13721
0.17866

C 0. -1.29372
0.87204

H 0.88605 -1.35345
1.51402

H -0.88605 -1.35345
1.51402

H 0. -2.13721 0.17866

1-azabicyclo[3.3.1]nonan-2-one
The number of imaginary frequencies: 0
Total Energy (B3LYP/6-31G*)= -
442.65877326
ZPE= -442.45197
Enthalpy= -442.443029

C -0.49637 -1.28822 -
0.57938

C -0.35609 1.22423
0.35726

C -1.62086 0.84888 -
0.48922

H 0.00119 -0.79517 -
1.38823

H -0.49982 -2.34298 -
0.75929

H -1.46437 1.24938 -
1.46902

H -2.51443 1.2602 -
0.06822

C 1.4982 -0.78156
0.27343

C 1.04761 1.20895 -
0.41451

H 1.80583 1.4413
0.30382

H 1.00949 1.96103 -
1.17466

H -0.49512 2.21928
0.7253

C -0.27607 0.19476
1.4253

H 0.43419 0.4558 2.1818

H -1.23488 0.06857
1.8832

C 1.45124 -0.1463 -
1.11957

H 2.32082 -0.73557 -
1.32327

H 2.01282 0.54207 -
1.71596

C -1.83906 -0.69692 -
0.48923

H -2.27832 -1.03664
0.42539

H -2.48041 -0.96277 -
1.30342

N 0.18165 -1.01827
0.72304

O 2.54604 -1.04261
0.91953

1-azabicyclo[3.3.1]nonan-2-one
The number of imaginary frequencies: 0
Total Energy (M06/6-311G+(d,p))= -
442.46542043
ZPE= -442.260754
Enthalpy= -442.251891

C 1.47992 1.13475
0.30259

C 0.06654 -1.51858
0.09096

C 1.03437 -0.97622 -
0.99587

H 1.31198 1.97218 -
0.37847

H 2.1127 1.48961
1.12406

H 0.46244 -0.40392 -
1.74022

H 1.48569 -1.80863 -
1.54549

C -0.91909 1.04429
0.01271

C -1.40185 -1.41106 -
0.3435

H -1.9993 -2.1865 0.1493

H -1.48843 -1.58693 -
1.42171

H 0.31208 -2.56073
0.32929

C 0.20236 -0.63269
1.32386

H -0.60147 -0.80755
2.04679

H 1.14083 -0.77841
1.86588

C -1.9898 -0.02338
0.01336

H -2.44866 -0.06345
1.00852

H -2.76771 0.28707 -
0.68583

C 2.11858 -0.06536 -
0.42347

H 2.75523 -0.63713
0.26438

H 2.77868 0.29181 -
1.22024

N 0.1682 0.73597
0.81707

O -0.9722 2.04129 -
0.67495

1-azabicyclo[3.3.1]nonan-2-one N-oxide
The number of imaginary frequencies: 0
Total Energy (B3LYP/6-31G*)= -
517.78066499
ZPE= -517.570525
Enthalpy= -517.56056

C 1.8199 -0.07291 -
0.18609

C -0.76203 -1.18497
0.66379

C -0.00207 -1.5653 -
0.65429

H 2.02216 0.36127 -
1.1429

H 2.69605 -0.01968
0.42582

H -0.13215 -0.83388 -
1.42435

H -0.37165 -2.50766 -
1.0011

C -0.38989 0.89081 -
0.63882

C -2.10937 -0.57304
0.23742

H -2.85726 -0.58977
1.00245

H -2.47067 -1.11804 -
0.60954

H -0.91426 -2.04993 1.275

C 0.07376 -0.11999
1.4419

H -0.58188 0.41577
2.09611

H 0.86954 -0.54064
2.02042

C -1.77131 0.88819 -
0.03872

H -1.70032 1.40413
0.89599

H -2.5119 1.3659 -
0.64553

C 1.43965 -1.57586 -
0.30576

H 1.54267 -2.10519
0.61841

H 2.06768 -2.03641 -
1.0395

N 0.67578 0.69627
0.38612

O -0.16179 1.01346 -
1.87028

O 1.13924 1.89388
0.83393

1-azabicyclo[3.3.1]nonan-2-one N-oxide
The number of imaginary frequencies: 0
Total Energy (M06/6-311G+(d,p))= -
517.5822033
ZPE= -517.374803
Enthalpy= -517.364771

C -1.54201 -1.00243 -0.1217

C -0.0647 1.58133
0.50268

C -1.07029 1.45316 -
0.69354

H -1.40068 -1.63532 -
0.99732

H -2.11124 -1.5535
0.62939

H -0.52328 1.20489 -
1.61085

H -1.54772 2.42095 -0.881

C 0.90566 -0.73077 -
0.56889

C 1.41296 1.64627
0.03022

H 2.02105 2.18527
0.76515

H 1.48025 2.20418 -
0.91013

H -0.30152 2.48045
1.08175

C -0.19775 0.3506
1.40313

H 0.63236 0.2215
2.09731

H -1.12641 0.3022
1.97627

C 2.00555 0.21376 -
0.15333

H 2.4325 -0.14896
0.78555

H 2.78694 0.18609 -
0.9149

C -2.15185 0.37882 -
0.45584

H -2.80987 0.70143
0.3603

H -2.786 0.29039 -
1.34461

N -0.17465 -0.84878
0.48961

O 0.79199 -1.30301 -
1.62165

O 0.29435 -1.97981
1.10659

1-aza-2-adamantanone
The number of imaginary frequencies: 0
Total Energy (B3LYP/6-31G*)= -
480.75695031
ZPE= -480.543716
Enthalpy= -480.53497

C -0.6513 -0.25597 -
0.01662

H 0.41848 -0.26424 -
0.03661

H -0.9986 0.75597 -
0.03286

C -1.15275 -0.95091
1.25587

H -0.79286 -0.43121
2.11917

C -1.20094 -1.00657 -1.23708

H -0.86636 -0.52267 -2.13083

C -0.71689 -2.45467 -1.22808

H 0.35164 -2.48423 -1.27557

H -1.12553 -2.96348 -2.07603

C -0.61547 -2.42209 1.28233

H -0.94244 -2.91128 2.17602

H 0.45402 -2.42786 1.24982

C -1.19071 -3.14143 0.06084

H -0.89325 -4.16811 0.01238

C -2.68548 -3.00313 0.25131

C -2.72471 -0.92451 1.23932

H -3.06625 0.08879 1.20103

H -3.09797 -1.39303 2.12593

C -2.73613 -1.01635 -1.19312

H -3.0999 -0.01061 -1.22577

H -3.10361 -1.55895 -2.03896

N -3.21549 -1.66257 0.04943

O -3.40757 -3.96161 0.63011

1-aza-2-adamantanone

The number of imaginary frequencies: 1 (-15.00 cm^{-1})

Total Energy (M06/6-311G+(d,p))= -480.54649801

ZPE= -480.33593

Enthalpy= -480.327979

C 2.00481 0.00013 -0.04122

H 2.67887 0.00047 0.82639

H 2.63927 -0.00004 -0.93848

C 1.11021 1.25758 -0.02471

H 1.72717 2.16493 -0.06729

C 1.11045 -1.25744 -0.02404

H 1.72752 -2.16474 -0.06615

C 0.2477 -1.26374 1.25372

H 0.88008 -1.26535 2.15186

H -0.37137 -2.16957 1.29419

C 0.24749 1.26438 1.25307

H -0.37169 2.17016 1.29311

H 0.87992 1.26657 2.15117

C -0.65208 0.00028 1.26354

H -1.326 0.00044 2.12563

C -1.4899 -0.00011 -0.01355

C 0.16561 1.21374 -1.24339

H 0.73161 1.20954 -2.18355

H -0.49161 2.09166 -1.26358

C 0.16581 -1.2144 -1.24274

H 0.7318 -1.21063 -2.18291

H -0.49122 -2.09247 -1.26242

N -0.69008 -0.00042 -1.23426

O -2.69679 -0.00007 -0.04028

1-aza-2-adamantanone N-oxide
The number of imaginary frequencies: 0
Total Energy (B3LYP/6-31G*)= -555.89761117
ZPE= -555.680574
Enthalpy= -555.670963

C 1.92654 -0.91079 0.05192

H 2.90664 -0.48151 0.0573

H 2.00627 -1.97736 0.08316

C 1.18071 -0.489 -1.22029

H 1.71273 -0.83763 -2.0807

C 1.13648 -0.41925 1.27139

H 1.63833 -0.71952 2.16743

C 1.02728 1.11812 1.23801

H 2.00369 1.55546 1.25384

H 0.47476 1.45332 2.0908

C 1.07764 1.05448 -1.27331

H 0.56135 1.34726 -2.16361

H 2.05585 1.48808 -1.27262

C 0.29382 1.52986 -0.04413

H 0.17657 2.59319 -0.06599

C -1.05166 0.82736 -0.07366

C -0.24342 -1.11093 -1.19633

H -0.1666 -2.17752 -1.15915

H -0.76856 -0.82057 -2.08222

C -0.28367 -1.03432 1.23486

H -0.21067 -2.10136 1.26666

H -0.83664 -0.68728 2.08261

N -0.98597 -0.62979 -0.0068

O -2.14002 1.45544 -0.00612

O -2.23926 -1.15783 -0.00146

1-aza-2-adamantanone N-oxide
The number of imaginary frequencies: 0
Total Energy (M06/6-311G+(d,p))= -555.68378514
ZPE= -555.468781
Enthalpy= -555.459194

C 1.9256 -0.93904 0.0004

H 2.94464 -0.53161
0.00035

H 2.0157 -2.03288
0.00072

C 1.16406 -0.46823 -
1.25524

H 1.67592 -0.81714 -
2.16103

C 1.16386 -0.4675
1.25566

H 1.6756 -0.81587
2.16173

C 1.07179 1.06953 1.2666

H 2.0725 1.51978
1.27521

H 0.55193 1.41737
2.16774

C 1.07204 1.06882 -
1.26708

H 0.55238 1.41614 -
2.16856

H 2.07277 1.51903 -
1.27579

C 0.3158 1.54057 -
0.00047

H 0.18723 2.62743 -
0.00074

C -1.07154 0.92176 -
0.0004

C -0.25347 -1.05592 -
1.24346

H -0.26869 -2.14847 -
1.21878

H -0.86894 -0.72495 -
2.08433

C -0.25363 -1.05515
1.24399

H -0.26901 -2.1477
1.21991

H -0.86942 -0.72356
2.08437

N -1.04552 -0.64681 -
0.00006

O -2.12403 1.48192 -
0.00011

O -2.2461 -1.1968
0.00006

Acetone
The number of imaginary frequencies: 0
Total Energy (B3LYP/6-31G*)= -
193.15569459
ZPE= -193.071622
Enthalpy= -193.065254

C 0. 0. 0.15196

O 0. 0. 1.41036

C 0. -1.33368 -0.61804

H -0.5044 -1.20574 -
1.55298

H 1.00881 -1.64256 -
0.79637

H -0.5044 -2.07939 -
0.03977

C 0. 1.33368 -0.61804

H 0.5044 1.20574 -
1.55298

H -1.00881 1.64256 -
0.79637

H 0.5044 2.07939 -
0.03977

Acetone
The number of imaginary frequencies: 0
Total Energy (M06/6-311G+(d,p))= -
193.07885176
ZPE= -192.996166
Enthalpy= -192.990644

C 0. 0. 0.15196

O 0. 0. 1.41036

C 0. -1.33368 -0.61804

H -0.5044 -1.20574 -1.55298

H 1.00881 -1.64256 -0.79637

H -0.5044 -2.07939 -0.03977

C 0. 1.33368 -0.61804

H 0.5044 1.20574 -1.55298

H -1.00881 1.64256 -0.79637

H 0.5044 2.07939 -0.03977

Hydrogen Peroxide
The number of imaginary frequencies: 1 (-261.15 cm^{-1})
Total Energy (B3LYP/6-31G*)= -151.53208497
ZPE= -151.506399
Enthalpy= -151.502562

O -0.64299 -0.14886 0.

H -1.15889 0.66074 0.

O 0.64299 0.14886 0.

H 1.15889 -0.66074 0.

Hydrogen Peroxide
The number of imaginary frequencies: 1 (-377.89 cm^{-1})
Total Energy (M06/6-311G+(d,p))= -151.52110268
ZPE= -151.494886
Enthalpy= -151.491055

O -0.64299 -0.14886 0.

H -1.15889 0.66074 0.

O 0.64299 0.14886 0.

H 1.15889 -0.66074 0.

Methylacetate
The number of imaginary frequencies: 0
Total Energy (B3LYP/6-31G*)= -268.38848966
ZPE= -268.298165
Enthalpy= -268.291067

C 0.27318 0.83923 0.15226

O 1.49767 0.86066 0.44164

C -0.45023 -0.50727 -0.03539

H -1.47835 -0.40142 0.24149

H -0.38598 -0.80684 -1.06059

H 0.011 -1.24911 0.58254

O -0.44654 2.0652 -0.00234

C -0.37666 2.49027 -1.36591

H -0.9152 3.4076 -1.48159

H 0.6465 2.64024 -1.64077

H -0.80901 1.74102 -1.9956

Methylacetate
The number of imaginary frequencies: 0
Total Energy (M06/6-311G+(d,p))= -268.30586312
ZPE= -268.216855
Enthalpy= -268.209704

C -0.46052 0.17822 -0.00005

O -0.28541 1.37656 -
0.00001

C -1.80139 -0.51814
0.00003

H -1.89229 -1.16153 -
0.88128

H -1.89317 -1.15942
0.88279

H -2.5971 0.2274 -
0.00121

O 0.5495 -0.72391 -
0.00009

C 1.87184 -0.16319
0.00006

H 2.55177 -1.01579
0.00065

H 2.02949 0.45296 -
0.88939

H 2.02897 0.45383
0.88898

Water
The number of imaginary frequencies: 0
Total Energy (B3LYP/6-31G*)= -
76.40895332
ZPE= -76.387785
Enthalpy= -76.384006

O	-0.00008	-0.8136	0.
H	0.95992	-0.81336	0.
H	-0.3203	0.09142	0.

Water
The number of imaginary frequencies: 0
Total Energy (M06/6-311G+(d,p))= -
76.41784139
ZPE= -76.396247
Enthalpy= -76.392466

O	0.	0.	0.11972
H	0.	0.76156	-0.47888
H	0.	-0.76156	-0.47888

Table 3 Structures

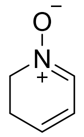

1-Aza-1,3-cyclohexadiene N-oxide

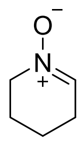

1-Azacyclohexene N-oxide

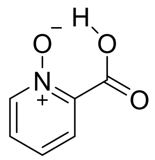

2-Carboxylpyridine N-oxide

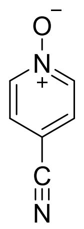

4-Cyanopyridine N-oxide

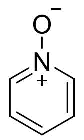

Pyridine N-oxide (PNO)

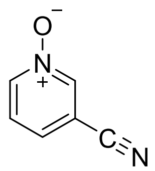

3-Cyanopyridine N-oxide

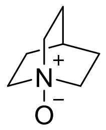

1-Azabicyclo[2.2.2]octane N-oxide

(Quinuclidine-N-oxide)

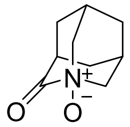

1-Azaadamantane-2-one N-oxide

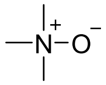

Trimethylamine N-oxide (TMAO)

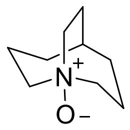

1-Azabicyclo[3.3.2]decane N-oxide

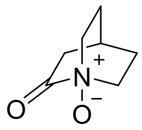

1-Azabicyclo[2.2.2]octan-2-one N-oxide

(2-Quinuclidinone N-oxide)

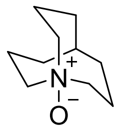

1-Azabicyclo[3.3.3]undecane N-oxide

(Manxine N-oxide)

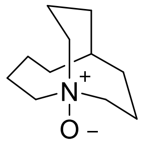

1-Azabicyclo[4.3.3]dodecane N-oxide

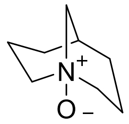

1-Azabicyclo[3.3.1]nonan-2-one N-oxide

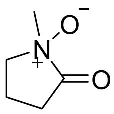

N-Methyl-2-pyrrolidinone N-oxide

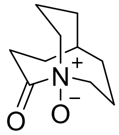

1-Azabicyclo[3.3.3]undecane-2-one N-oxide

(2-Manxinone N-oxide)
